# Dissecting the conformational complexity and mechanism of a bacterial heme transporter

**DOI:** 10.1038/s41589-023-01314-5

**Published:** 2023-04-24

**Authors:** Di Wu, Ahmad R. Mehdipour, Franziska Finke, Hojjat G. Goojani, Roan R. Groh, Tamara N. Grund, Thomas M. B. Reichhart, Rita Zimmermann, Sonja Welsch, Dirk Bald, Mark Shepherd, Gerhard Hummer, Schara Safarian

**Affiliations:** 1 Department of Molecular Membrane Biology, Max Planck Institute of Biophysics, Frankfurt/Main, Germany; 2 Department of Theoretical Biophysics, Max Planck Institute of Biophysics, Frankfurt/Main, Germany; 3 Center for Molecular Modeling (CMM), Ghent University, Zwijnaarde, Belgium; 4 Amsterdam Institute for Life and Environment (A-LIFE), AIMMS, Faculty of Science, Vrije University of Amsterdam, Amsterdam, the Netherlands; 5 Central Electron Microscopy Facility, Max Planck Institute of Biophysics, Frankfurt am Main, Germany; 6 School of Biosciences, RAPID Group, University of Kent, Canterbury, UK; 7 Institute of Biophysics, Goethe University Frankfurt, Frankfurt/Main, Germany; 8 Department of Microbiology and Immunology, School of Biomedical Sciences, University of Otago, Dunedin, New Zealand; 9 Fraunhofer Institute for Translational Medicine and Pharmacology ITMP, Frankfurt/Main, Germany

**Keywords:** Structural biology, Bacteria, Computational chemistry, Transporters

## Abstract

Iron-bound cyclic tetrapyrroles (hemes) are redox-active cofactors in bioenergetic enzymes. However, the mechanisms of heme transport and insertion into respiratory chain complexes remain unclear. Here, we used cellular, biochemical, structural and computational methods to characterize the structure and function of the heterodimeric bacterial ABC transporter CydDC. We provide multi-level evidence that CydDC is a heme transporter required for functional maturation of cytochrome *bd*, a pharmaceutically relevant drug target. Our systematic single-particle cryogenic-electron microscopy approach combined with atomistic molecular dynamics simulations provides detailed insight into the conformational landscape of CydDC during substrate binding and occlusion. Our simulations reveal that heme binds laterally from the membrane space to the transmembrane region of CydDC, enabled by a highly asymmetrical inward-facing CydDC conformation. During the binding process, heme propionates interact with positively charged residues on the surface and later in the substrate-binding pocket of the transporter, causing the heme orientation to rotate 180°.

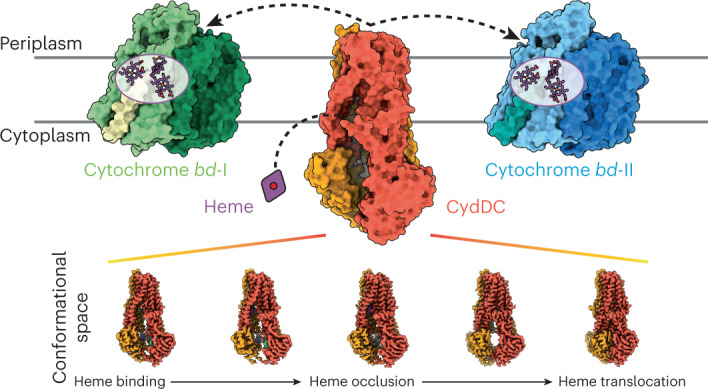

## Abstract (web summary)

Characterization of the conformation landscape of the bacterial ABC transporter CydDC reveals that this heterodimeric protein complex is a long-sought heme transporter essential for the functional maturation of *bd*-type cytochromes.

## Main

Iron is the second most abundant metal on our planet and an essential trace element for all domains of life^1–6^. It is involved in a multitude of physiological processes such as photosynthesis, protein biosynthesis and respiration. Cellular iron is found in the form of iron-sulfur clusters (Fe-S), iron-bound cyclic tetrapyrroles (hemes) or in its free ionic forms. In biological systems, iron mediates electron transfer by acting as electron acceptor or donor in various biochemical reactions^7^. Terminal respiratory oxidases are metalloproteins that rely on their redox-active heme cofactors for reduction of molecular oxygen to water^8–15^. Knowledge about the mechanism of maturation and heme insertion of cytochrome *bd*-type oxidases remains unknown but is urgently required for the development of antimicrobial drugs that can specifically target the energy metabolism and respiratory rewiring of human pathogenic bacteria upon infection and proliferation^16,17^.

The ABC transporter CydDC plays a central role in the biogenesis of membrane-integrated and soluble cytochromes^18–22^. Its precise function and molecular mode of action have remained enigmatic for decades^23,24^. CydDC was initially thought to translocate heme across the bacterial cytoplasmic membrane. Follow-up studies, however, concluded that CydDC might be involved in the regulation of the periplasmic redox poise by transporting the reductants glutathione (GSH) and l-cysteine (l-Cys) to the periplasm^19,21,25–28^. Despite this widely accepted substrate specificity of CydDC, it was later found that the CydDC complex copurifies with bound heme^29^. This observation motivated the assumption that heme represents a prosthetic group of a periplasmic redox sensor domain for regulation of transporter activity^18,23^. A recent work challenged the transporter function of CydDC and proposed that it operates as a cystine reductase^30^.

To resolve these points of controversy, we used *Escherichia coli* (*E. coli*) as a model system amenable to our integrative approach including growth-complementation studies, biochemical activity assays, systematic single-particle cryogenic-electron microscopy (cryo-EM) and atomistic molecular dynamics (MD) simulations to characterize the structure and function of CydDC down to the molecular level.

## Results

### CydDC function is linked to biogenesis of cytochrome *bd*

**Fig. 1:**
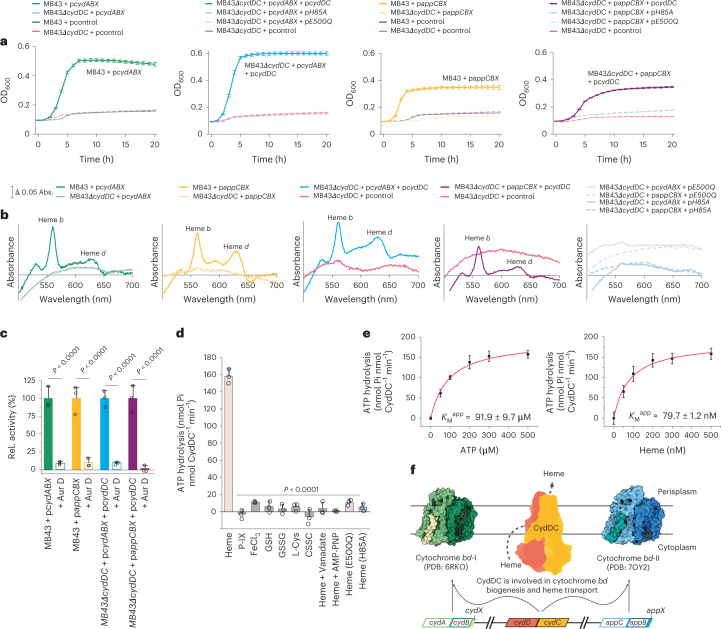
Physiological and biochemical links between CydDC activity and cytochrome *bd* biogenesis. **a**, *E. coli* growth-complementation studies. Plasmid encoded cytochrome *bd* variants were p*cydABX*, cyt. *bd*-I; p*appCBX*, cyt. *bd*-II. Plasmid encoded CydDC variants were p*cydDC*, wild-type; p*E500Q*, E->Q exchange at position 500 of CydC; p*H85A*, H->A exchange at position 85 of CydC. Control plasmid was pcontrol, empty pET17 vector. Data are presented as mean values ±s.d. (*n* = 3 biological replicates). **b**, Reduced-minus-oxidized UV-vis spectra of membrane fractions from strains with restored and/or impaired growth phenotypes. **c**, Oxygen reductase activity of membranes from strains with growth-active phenotypes. Aurachin D (Aur. D) induced inhibition of oxygen consumption indicates for cytochrome *bd* specific oxygen reductase activity. Data are presented as mean values ±s.d. (*n* = 3 biological replicates). Significance was assessed based on a paired two-tailed Student’s *t*-test. Rel., relative. **d**, Stimulation of ATP hydrolysis activity (malachite green phosphate assay) of CydDC. Significance was assessed based on a paired two-tailed Student’s *t*-test. All presented ATP hydrolysis data are corrected for background activity in the absence of substrate candidates. Data are presented as mean values ± s.d. (*n* = 3 assay technical replicates). CSSC, cystine; GSSG, glutathione disulfide; P-IX, protoporphyrin IX. **e**, Titration experiments for the determination of Michaelis constants (*K*_M_^app^) for ATP (91.9 ± 9.7 µM) and heme (79.7 ± 1.2 nM). All presented ATP hydrolysis data are corrected for background activity in the absence of substrate candidates. Data are presented as mean values ± s.d. (*n* = 3 assay technical replicates). **f**, Working model of the physiological role of CydDC based on independent physiological and biochemical data generated in this study. Source data

We performed growth-complementation studies using the *E. coli* mutant strain MB43, which lacks all terminal oxidases and thus shows impaired respiratory activity (Fig. 1)^31^. The growth of this strain is poor compared to wild-type controls, but was improved following the introduction of genes encoding cytochromes *bd*-I (p*cydABX*) or *bd*-II (p*appCBX*) (Fig. 1a and Supplementary Fig. 1). Next, we constructed an isogenic deletion mutant of the *cydDC* operon in the genetic background of MB43. This strain (MB43Δ*cydDC*) shows an indistinguishable phenotype to MB43 (Fig. 1a). Unlike MB43, the growth of MB43Δ*cydDC* was not restored by introducing p*cydABX* or p*appCBX*. A growth-active phenotype of MB43Δ*cydDC* was only achieved by double complementation with p*cydDC* and either p*cydABX* or p*appCBX* (Fig. 1a). Accordingly, ultraviolet-visible light (UV-vis) spectroscopy revealed that the characteristic fingerprints of cytochrome *bd* cofactors (hemes *b* and *d*) were absent in membrane fractions of MB43 and MB43Δ*cydDC* but clearly detected in the growth-restored complementation strains (Fig. 1b)^31–34^.

Further, we confirmed oxygen reductase activity of membrane fractions from growth-restored MB43 and MB43Δ*cydDC* variants (Fig. 1c and Supplementary Fig. 2a). Finally, we used the highly specific inhibitor aurachin D to demonstrate that restored oxygen consumption was due to the activity of cytochrome *bd* variants (Fig. 1c). In summary, growth-complementation studies, UV-vis spectroscopy and oxygen consumption assays confirmed that CydDC is essential for the functional maturation of both known variants of cytochrome *bd* of *E. coli* (Fig. 1f).

### ATPase activity of CydDC is stimulated by heme

**Extended Data Fig. 1:**
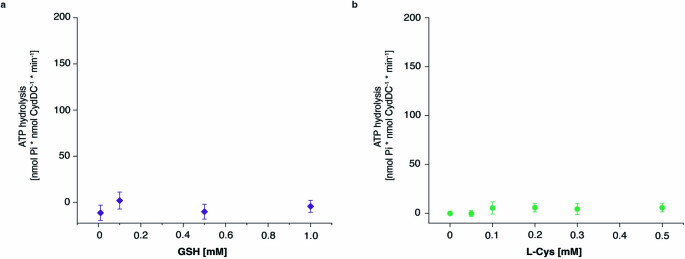
GSH and L-Cys dependent ATPase activity of CydDC. Effects of **(a)** GSH (0.01–1 mM) and **(b)** L-Cys (0.05–0.5 mM) on the ATPase activity of CydDC determined by a malachite green phosphate assay. All presented ATP hydrolysis data are corrected for background activity in the absence of substrate candidates. Data are presented as mean values ±s.d. (n = 3 assay technical replicates). Source data

**Extended Data Fig. 2:**
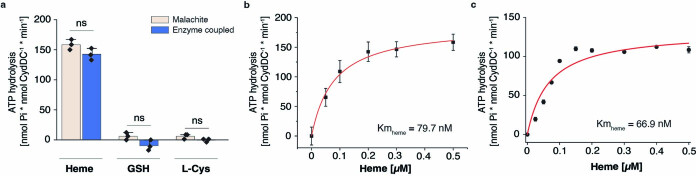
Influence of heme vs. thiols on the ATPase activity of CydDC. **(a)** ATP hydrolysis activity of CydDC was determined in the presence of heme (500 nM), GSH (1 mM), and L-Cys (0.5 mM) using the malachite green phosphate and the PK/LDH coupled assays, respectively. Significance was assessed based on a paired two-tailed Student’s t-test. Data are presented as mean values ±s.d. (n = 3 assay technical replicates)**. (b, c)** Michaelis constants (*K*_M_^app^) of CydDC for heme determined by malachite green phosphate (79.7 nM) and PK/LDH coupled assays (66.9 nM), respectively. All presented ATP hydrolysis data are corrected for background activity in the absence of substrate candidates. Data are presented as mean values ±s.d. (n = 3 assay technical replicates). Ns, not significant. Source data

We next tested affinity-purified CydDC for ATPase activity in the presence of putative substrates, primarily using the malachite green phosphate assay (Fig. 1d and Supplementary Fig. 3)^26,29,30,35^. In contrast to previous reports, we found that GSH and l-Cys failed to induce hydrolysis, whereas heme stimulated the ATPase activity of CydDC in a concentration-dependent manner (Fig. 1d,e and Extended Data Fig. 1). This effect was only observed for heme itself, but not for its macrocycle scaffold protoporphyrin IX or free iron, suggesting that the complexed iron molecule might play a critical role in binding and coordination (Fig. 1d). For verification of our results, we used a second conceptually different enzyme-coupled ATP hydrolysis assay (Extended Data Fig. 2a). Both assays confirm the ATPase stimulating effect of heme while showing that the tested reductants do not show typical effects of ABC transporter substrates. To bolster our finding of heme as a primary candidate for a substrate molecule of CydDC, we determined kinetic parameters for heme and ATP. We obtained *K*_M_^app^ values of 79.7 nM for heme, and 91.9 µM for ATP (Fig. 1e and Extended Data Fig. 2b,c). These values are reasonable regarding the physiological occurrence of both substrates^36,37^.

**Extended Data Fig. 3:**
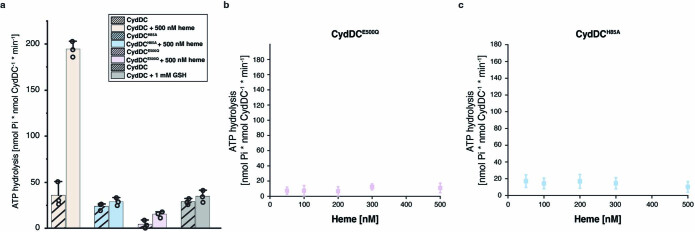
Mutant variants influence the ATPase activity of CydDC. **(a)** Total ATPase activities of wild-type, H85A^C^, and E500Q^C^ variants of CydDC show that unspecific background hydrolysis activity is slightly reduced by the histidine to alanine exchange of the characterized heme binding site, and strongly by the Walker B glutamate to glutamine exchange in NBS^C^. Total ATPase activity of wild-type CydDC in the presence and absence of GSH exemplifies a non-stimulatory effect. Data are presented as mean values ±s.d. (n = 3 assay technical replicates)**. (b)** Heme (50–500 nM) dependent ATP hydrolysis activity of CydDC^E500Q^ determined by malachite green phosphate assay. **(c)** Heme (50–500 nM) dependent ATP hydrolysis activity of CydDC^H85A^ determined by malachite green phosphatase assay. All presented ATP hydrolysis data are corrected for background activity in the absence of heme. Data are presented as mean values ±s.d. (n = 3 assay technical replicates). Source data

Next, we generated glutamate to glutamine mutants of the Walker B domains of CydD and CydC to determine whether CydDC possesses a functionally degenerate nucleotide-binding domain (NBD) as found in other ABC transporters^38^. Of the two constructed Walker B variants, only CydDC^E500Q^ could be isolated in sufficient yield, purity and stability (Supplementary Fig. 3). Using heme as substrate, we showed that the conserved Walker B glutamate of CydC is essential for ATPase activity of CydDC (Fig. 1d and Extended Data Fig. 3a,b). Complementation of MB43Δ*cydDC* with the gene product of p*E500Q* (CydDC^E500Q^) in combination with either p*cydABX* or p*appCBX* did not restore bacterial growth (Fig. 1a,b and Supplementary Fig. 2b,c). We thus conclude that NBD^C^ represents the canonical hydrolysis site and that CydD most presumably contains a degenerate NBD.

To verify our findings on the role of heme as a potential substrate molecule and to dissect the molecular basis of CydDC function, we designed a matrix of sample conditions based on a combination of putative substrate molecules, nucleotides that are either susceptible or resistant to hydrolysis and rationally designed mutant variants of CydDC. In total, we determined single-particle cryo-EM structures of CydDC under 23 different conditions at resolutions of 2.7 to 3.9 Å (Supplementary Table 1 and Supplementary Figs. 3–9).

### Overall architecture and insights into substrate specificity

**Fig. 2:**
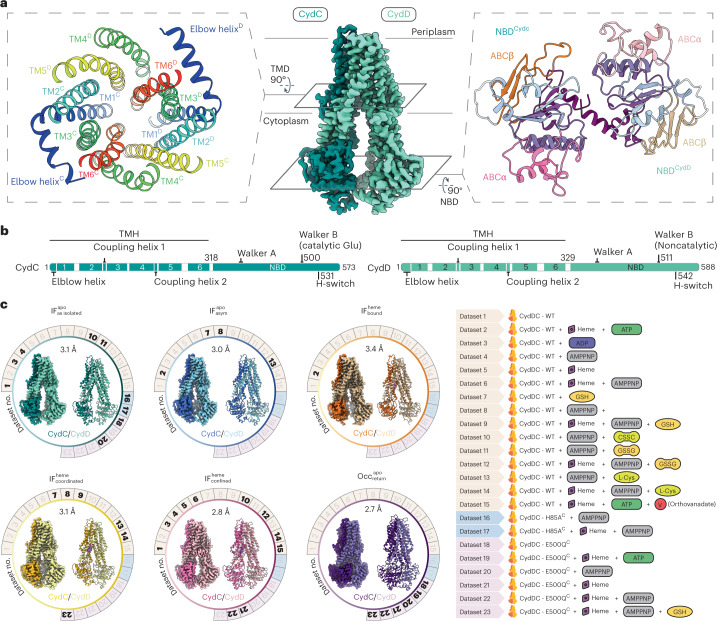
Systematic cryo-EM approach. **a**, Volume map representation of the ${{{\mathrm{IF}}}}_{{{{\mathrm{as}}}}\;{{{\mathrm{isolated}}}}}^{{{{\mathrm{heme}}}}}$ structure representing the general architecture of CydDC. Dark green, CydC; light green, CydD. The left shows a cross-section of the membrane domain composed of 12 TMs displaying arrangement and geometry typical for type IV ABC transporters. The right shows a cross-section of NBDs. The subdomains ABCα and ABCβ of each NBD are highlighted. **b**, Schematic organization of CydC and CydD subunits. TMs are indicated by numbers. Residues important for ATP hydrolysis and signal transduction between TMH and NB domains are highlighted. **c**, Summary of systematic single-particle cryo-EM studies. The left shows volume maps and corresponding ribbon models. Each circle represents a distinct conformation of CydDC. Numbers around circles refer to sample condition of CydDC analyzed by electron microscopy. Bold numbers indicate that the given conformation was present under that condition. The right shows a summarized information panel about the analyzed CydDC variants, presence and absence of heme, nucleotide and additional putative substrate molecules. CydDC^wt^, beige; CydDC^H85A^, light blue; CydDC^E500Q^, purple. IF, inward-facing; Occ, occluded; GSSG, glutathione disulfide; CSSC, cystine.

The nucleotide and substrate-free, inward-facing conformation of CydDC $\left( {{{{\mathrm{IF}}}}_{{{{\mathrm{as}}}}\;{{{\mathrm{isolated}}}}}^{{{{\mathrm{apo}}}}}} \right)$, obtained in as isolated conditions, exhibits canonical features of type IV ABC transporters (Fig. 2a,b)^39–41^. Each subunit is composed of a membrane domain formed by six transmembrane α-helices (TMH), an N-terminal cytoplasmic elbow helix oriented parallel to the membrane plane and a cytoplasmic NBD (Fig. 2a,b)^42^. In this conformation the heterodimer adopts an asymmetrical structure (Cα r.m.s.d. of 4.17 Å) (Supplementary Fig. 10).

**Extended Data Fig. 4:**
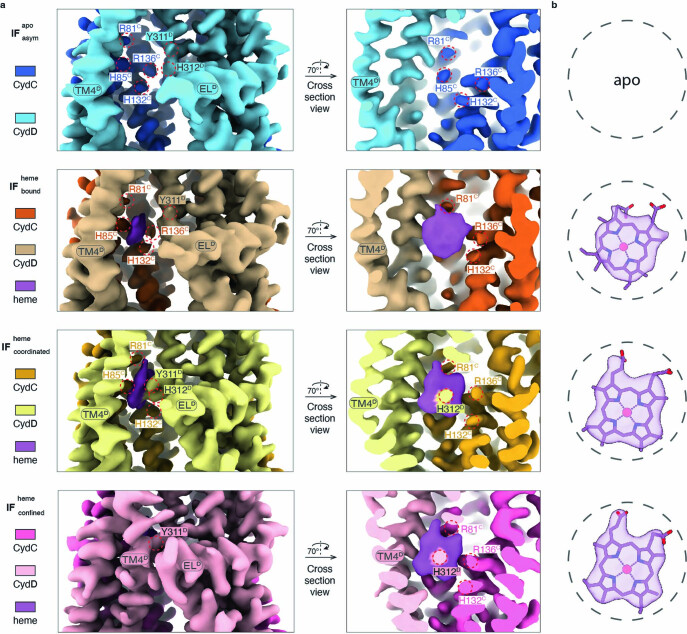
Location and binding mode of heme in different CydDC conformations. **(a)** Local density map of ${{{\mathbf{IF}}}}_{{{{\mathrm{asym}}}}}^{{{{\mathrm{apo}}}}}$, ${{{\mathbf{IF}}}}_{{{{\mathrm{bound}}}}}^{{{{\mathrm{heme}}}}}$, ${{{\mathbf{IF}}}}_{{{{\mathrm{coordinated}}}}}^{{{{\mathrm{heme}}}}}$, and ${{{\mathbf{IF}}}}_{{{{\mathrm{confined}}}}}^{{{{\mathrm{heme}}}}}$ at the heme binding site shown in side and cross-section views. **(b)** Heme models fitted into corresponding densities in maps of each conformational state. Density features of heme highlight that the heme molecule is rigidified during the process of binding and confinement.

The successful determination of the ${{{\mathrm{IF}}}}_{{{{\mathrm{as}}}}\;{{{\mathrm{isolated}}}}}^{{{{\mathrm{apo}}}}}$ conformation was followed by a screen for substrate molecules binding to CydDC (Fig. 2c). Of all tested compounds, we were only able to identify ligand density of heme bound within the TMH domain (Extended Data Fig. 4). We obtained heme bound CydDC conformations not only through exogenous heme addition, but consistently also from subsets of CydDC particles that natively copurified with heme in sample compositions free of heme (Fig. 2c).

In addition, we determined structures of CydDC in the presence of a combination of heme and thiols (GSH, glutathione disulfide (GSSG), l-Cys) to assess whether these molecules may be heme-dependent cosubstrates. However, we could identify ligand density only for heme but did not observe signal for any other added molecule.

### Heme binding induces sequential conformational transitions

**Fig. 3:**
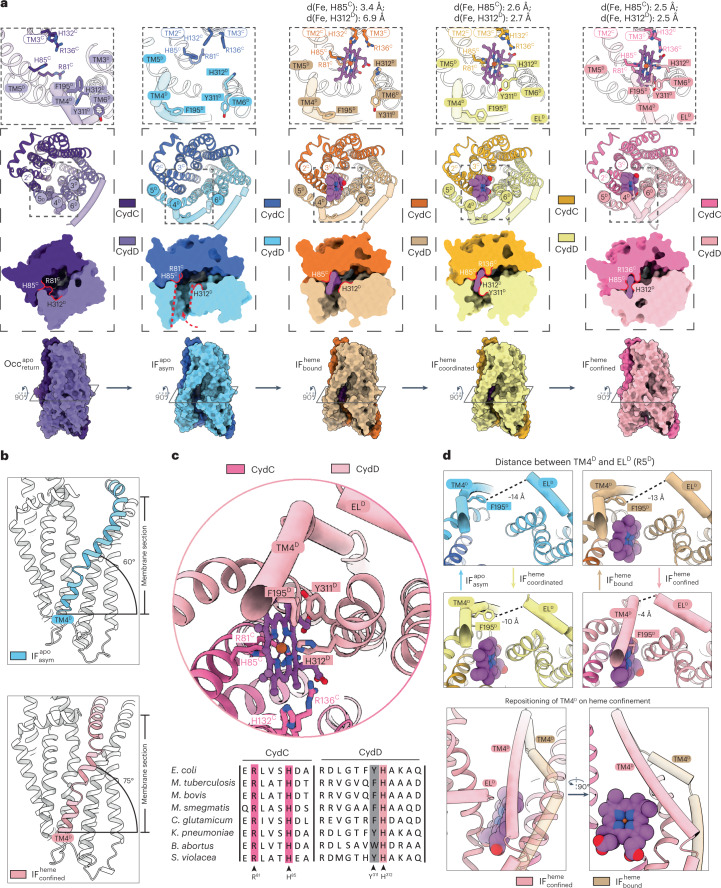
Conformational landscape of heme binding and translocation. **a**, Close-up views of residues involved in heme binding and occlusion. Surface model cross sections and side views illustrate the changing shape and size of the internal cavity and the TMH region in different conformational states. Heme is shown as purple ball-and-stick model. EL^D^ and TM4^D^ are shown as tubes. **b**, On heme entry and coordination, the membrane-embedded segment of TM4^D^ moves toward EL^D^ and thus closes the lateral gate of CydDC. **c**, The top shows the close-up view of the heme-binding site in the ${{{\mathrm{IF}}}}_{{{{\mathrm{confined}}}}}^{{{{\mathrm{heme}}}}}$ state. The bottom shows the sequence alignments of CydD and CydC heme binding-site forming regions of representative and disease related bacteria. Conserved residues are highlighted in magenta. Conserved amino acid groups are highlighted in gray. **d**, The top shows the changing EL^D^–TM4^D^ distance during the transition from ${{{\mathrm{IF}}}}_{{{{\mathrm{asym}}}}}^{{{{\mathrm{apo}}}}}$ to ${{{\mathrm{IF}}}}_{{{{\mathrm{confined}}}}}^{{{{\mathrm{heme}}}}}$ states. The bottom shows a close-up view of the heme entry site in ${{{\mathrm{IF}}}}_{{{{\mathrm{bound}}}}}^{{{{\mathrm{heme}}}}}$ (beige) and ${{{\mathrm{IF}}}}_{{{{\mathrm{confined}}}}}^{{{{\mathrm{heme}}}}}$ (magenta) states, respectively. EL, elbow helix; IF, inward-facing; Occ, occluded; asym, asymmetrical.

From the sum of analyzed sample conditions, we obtained three heme-bound states that provide comprehensive insight into substrate binding and occlusion (Figs. 2c and 3, Extended Data Fig. 4 and Supplementary Fig. 11). Heme initially binds within a cavity formed by transmembrane helices TM2^C^, TM3^C^, TM5^D^ and TM6^D^ that is in the vicinity of the lateral membrane plane (Fig. 3a, Extended Data Fig. 4a and Supplementary Fig. 11). In this ${{{\mathrm{IF}}}}_{{{{\mathrm{bound}}}}}^{{{{\mathrm{heme}}}}}$ conformation, which was obtained exclusively under active turnover, the heme molecule primarily interacts with the invariant H85^C^ residue that functions as an axial ligand for the heme iron at a distance of 3.4 Å (Figs. 2c and 3a). Additionally, the two heme propionate groups form electrostatic interactions with R81^C^ of TM2^C^ and R136^C^ of TM3^C^, respectively (Fig. 3a, Extended Data Fig. 4a, Supplementary Fig. 11 and Supplementary Video 1). We assign the ${{{\mathrm{IF}}}}_{{{{\mathrm{coordinated}}}}}^{{{{\mathrm{heme}}}}}$ state as the second step in the binding cascade. Here, the interaction between CydDC and heme is strengthened by movement of the C-terminal segment of TM6^D^ toward the heme and concomitant side chain rearrangements of H312^D^, which acts as a second axial ligand of the heme iron, and Y311^D^, which moves in front of the porphyrin plane and thereby prevents heme from escaping to the membrane space (Fig. 3a, Extended Data Fig. 4a, Supplementary Fig. 11 and Supplementary Videos 2 and 3). The interaction between TM6^D^ and EL^D^ (elbow helix) further causes positioning of the elbow helix closer toward TM4^D^, which narrows the membrane-accessible gate to the heme-binding site (Fig. 3d, Extended Data Fig. 4a, Supplementary Fig. 12 and Supplementary Videos 2 and 3).

The most abundant heme-bound conformation observed in our study is termed ${{{\mathrm{IF}}}}_{{{{\mathrm{confined}}}}}^{{{{\mathrm{heme}}}}}$ and represents the confined inward-facing TMH conformation (Figs. 2c and 3a). On binding and tight coordination of heme, a crucial conformational change of TM4^D^ is induced (Supplementary Video 4). Movement of this 7.2 nm long helix occurs on two levels (Supplementary Videos 5 and 6). The membrane-embedded segment of TM4^D^ (segments 164–195) changes its angle from roughly 60° to 75° relative to the membrane plane and thus moves in front of the heme, blocking the lateral membrane gate (Fig. 3b,c). The kinked C-terminal half of TM4^D^ that is exposed to the cytoplasm concomitantly moves toward EL^D^ and adopts a conformation that runs diagonally above the bound heme molecule (Fig. 3d). Accordingly, the side chain of F195^D^ moves in front of the heme and aligns in parallel with the porphyrin plane (Fig. 3a,c and Supplementary Fig. 11). Together with Y311 of TM6^D^, these aromatic residues generate π-π stacking interactions with the porphyrin plane of the heme. An electrostatic interaction network formed between EL^D^ and TM4 further contributes to the formation of a tightly sealed substrate access gate (Fig. 3a,c, Supplementary Figs. 11 and 12 and Supplementary Video 5). The formation of a distinct heme-binding pocket narrows the unoccupied space within the TMH domain and establishes a lateral pathway perpendicular to the membrane plane that ends at the tightly interlocked periplasmic substrate exit gate (Fig. 3a). Furthermore, we observed that binding and confinement of heme occurs independent of nucleotide occupancy of the NBDs (Fig. 2c).

**Extended Data Fig. 5:**
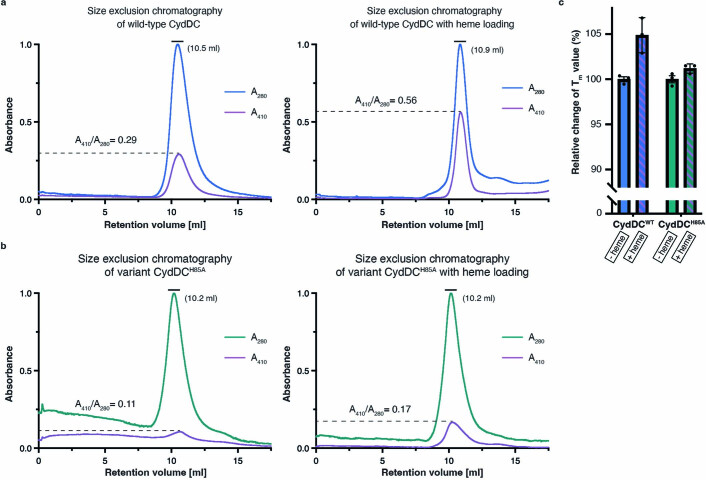
Heme binding properties of CydDC^wt^ and CydDC^H85A^. **(a)** Size exclusion chromatograms (SEC) of CydDC (wt) in the absence and presence of exogenously added heme (applied before the experiment). Wild-type CydDC contains bound heme from native source, which co-migrates with the peak fraction of the protein. Addition of extra heme increases the intensity of the A_410_ signal indicating greater heme occupancy. **(b)** SEC of CydDC^H85A^ in the absence and presence of exogenous heme (applied before the experiment). The chromatograms show that native heme does not copurify with the mutant variant CydDC^H85A^. Furthermore, adding exogenous heme does not have the same effect observed with the wild-type CydDC. The slight increase in the A_410_ signal is attributed to heme molecules migrating into hydrophobic regions of detergent micelles. Relative ratios of the peak intensities between A_410_ (heme component) and A_280_ (protein component) are given. **(c)** Relative change of T_M_ in the absence and presence of exogenously added heme molecules to purified samples of CydDC^wt^ and CydDC^H85A^. Data are presented as mean values ±s.d. (n = 3 assay technical replicates). Source data

Based on the deduced sequence of binding events and the distinct interaction patterns between heme and CydDC, we reasoned that the interaction between H85^C^ and the heme iron marks the most crucial step of substrate recognition. To test this hypothesis, we generated a CydDC H85A^C^ mutant and performed growth-restoring complementation studies and oxygen consumption measurements (Fig. 1a,b and Supplementary Fig. 2b,c). In addition, we investigated the effect of the histidine to alanine replacement on ATP hydrolysis activity (Fig. 1d). A lack of this highly conserved histidine residue abrogates the functionality of CydDC (Fig. 1a,b,d, Extended Data Fig. 3c and Supplementary Fig. 2b,c). Furthermore, CydDC^H85A^ could not be copurified with bound heme and could not be loaded by the exogenous addition of heme (Extended Data Fig. 5). These observations were confirmed by cryo-EM structures of CydDC^H85A^, exclusively adopting the ${{{\mathrm{IF}}}}_{{{{\mathrm{as}}}}\;{{{\mathrm{isolated}}}}}^{{{{\mathrm{apo}}}}}$ conformation, irrespective of presence or absence of exogenously added heme to the samples (Fig. 2c).

By analyzing the relationship between sample conditions and obtained CydDC conformations we recognized that the ${{{\mathrm{IF}}}}_{{{{\mathrm{coordinated}}}}}^{{{{\mathrm{heme}}}}}$ conformation was obtained only in the presence of the reductants GSH and l-Cys. We reason that the systematic absence of ${{{\mathrm{IF}}}}_{{{{\mathrm{confined}}}}}^{{{{\mathrm{heme}}}}}$ state in the presence of GSH and l-Cys together with the consistent observation of ${{{\mathrm{IF}}}}_{{{{\mathrm{coordinated}}}}}^{{{{\mathrm{heme}}}}}$ as the major heme-bound class indicates that these reductants cause opening of the closed lateral heme entry gate. A lack of clearly distinguishable ligand density in the cryo-EM maps of the respective datasets leaves open the question about the precise interaction sites of these thiols and the underlying molecular mechanisms that result in the stabilization of the ${{{\mathrm{IF}}}}_{{{{\mathrm{coordinated}}}}}^{{{{\mathrm{heme}}}}}$ conformation.

### Heme enters CydDC via the membrane and rotates by 180°

**Fig. 4:**
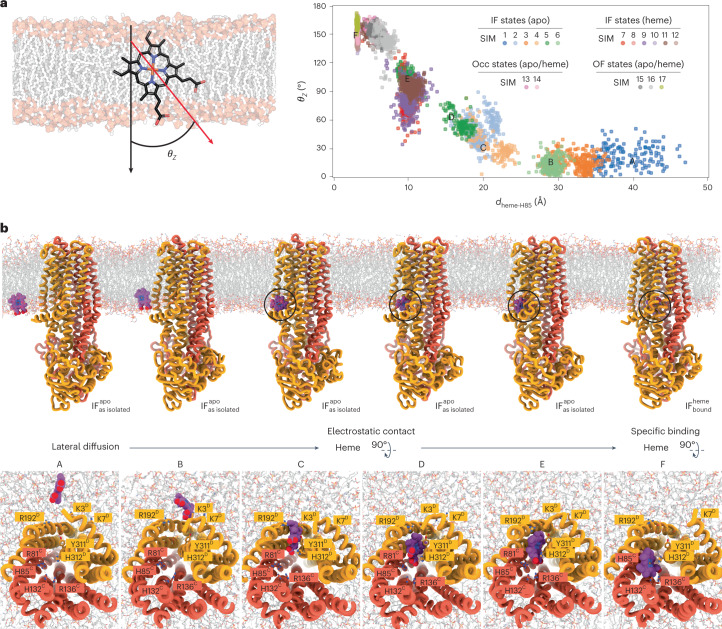
Structural dynamics of the heme binding and flipping mechanism. **a**, Distribution of heme rotation angle (*θ*_*Z*_) versus heme distance from the binding site at H85^C^ (*d*_heme-H85_) in different MD simulations of CydDC. *d*_heme-H85_ is defined as the distance between the centers of mass of heme molecules and the side chain of H85^C^. *θ*_*Z*_ is defined as the angle between two vectors: vector 1 (black) is axis normal to the membrane and vector 2 (red) is the vector connecting Cha and CHC atoms in heme. Representative snapshots of MD simulations showing the lateral diffusion and entry of heme to the membrane-accessible binding cavity of CydDC. **b**, Close-up views show that heme interacts with the positively charged electrostatic surface and the interior of the binding cavity of CydDC. The capital letters (A–F) represent the approximate positions of each snapshot in the cluster regions plotted in panel a.

**Extended Data Fig. 6:**
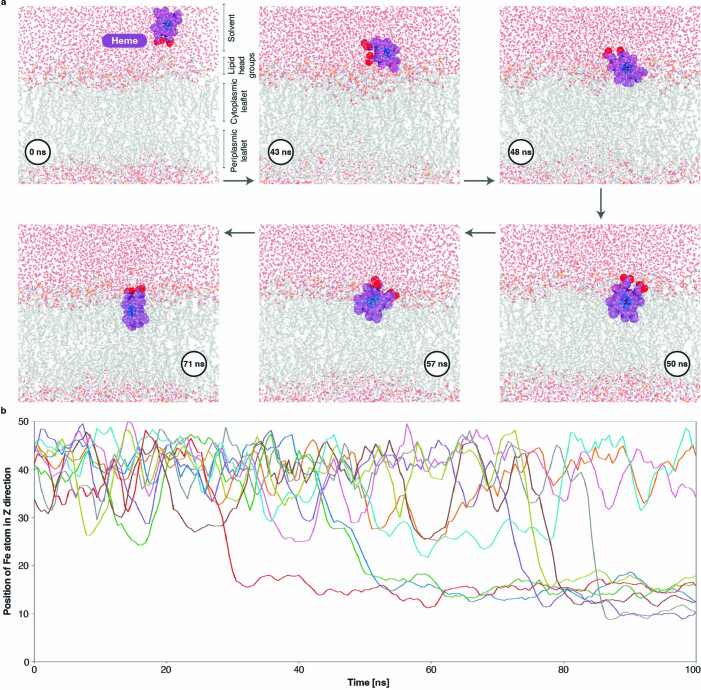
Heme partitions spontaneously into lipid bilayers. **(a)** Molecular dynamics simulations show spontaneous binding and insertion of heme into a lipid bilayer composed of 70% POPE, 25% POPG, and 5% CL lipids. Heme establishes a stable position in the membrane during 100-ns simulation runs. **(b)** During MD runs, position of the heme was tracked by the distance of the Fe atom to the center of the membrane in the Z-axis direction. Graph colors represent different simulation runs.

To map possible routes of heme entry, we carried out multiple atomistic MD simulations, focusing on the behavior of heme near lipid bilayer surfaces (70% palmitoyloleoyl phosphatidylethanolamine (POPE), 25% palmitoyloleoyl phosphatidylglycerol (POPG) and 5% cardiolipin) and the substrate-binding pocket (Fig. 4 and Extended Data Fig. 6). Our starting simulations showed that the porphyrin scaffold immersed deeply into the membrane core and interacted with lipid acyl chains, while the two propionate groups oriented toward the lipid head groups and interacted mostly with the ethanolamine groups of POPE molecules (Extended Data Fig. 6a). These observations are consistent with earlier experimental reports^43,44^.

Next, we analyzed the dynamics of heme placed near the lateral entry site of CydDC in the ${{{\mathrm{IF}}}}_{{{{\mathrm{as}}}}\;{{{\mathrm{isolated}}}}}^{{{{\mathrm{apo}}}}}$ state. Analysis of nonbonded interaction energies between heme and ${{{\mathrm{IF}}}}_{{{{\mathrm{as}}}}\;{{{\mathrm{isolated}}}}}^{{{{\mathrm{apo}}}}}$ shows that electrostatic interactions between K3^D^, K7^D^, K314^D^ and R192^D^ and the heme propionates play a key role in attracting heme toward the lateral entry site. These interactions keep heme in the original membrane-embedded orientation (*θ*_*Z*_ $\succcurlyeq$ 30°) (Fig. 4 and Supplementary Fig. 13). When we mutate the K3^D^, K7^D^ and K314^D^ to alanines, both the rotation angle (*θ*_*Z*_) increases and the distribution of the distance to the binding site (d_heme-H85_) is broadened (Supplementary Fig. 14). We further observe that once heme moves inside the cavity toward the defined binding pocket, its interactions with R77^C^, R81^C^ and R136^C^ become stronger while its interactions with the surface lysine groups diminish. Moreover, the interactions with R77^C^, R81^C^ and R136^C^ appear to be a key factor for driving a 90° rotation (*θ*_*Z*_ $\simeq$ 90°) of the heme. The mutation of R136^C^ to alanine in the ${{{\mathrm{IF}}}}_{{{{\mathrm{confined}}}}}^{{{{\mathrm{heme}}}}}$ state in the absence of axial ligation of heme narrows the conformational distribution (stronger clustering) at higher *θ*_*Z*_ angles showing the importance of this residue in keeping heme in the 90° rotation conformation. In this mutation (R136^C^A), the higher rotation angle is also linked to interactions with R77^C^ and R81^C^. The conformation of heme at *θ*_*Z*_ $\simeq$ 90° has the lowest interaction energy. Thus, the subsequently observed second 90° rotation (*θ*_*Z*_ $\simeq$ 180°) might deem unfavorable if we solely consider the nonbonded interaction energy (Supplementary Fig. 13), suggesting that covalent axial ligation with two coordinating histidines may be required for a full 180° rotation. When heme is axially ligated to H85^C^ in ${{{\mathrm{IF}}}}_{{{{\mathrm{bound}}}}}^{{{{\mathrm{heme}}}}}$, or both H85^C^ and H312^D^ in ${{{\mathrm{IF}}}}_{{{{\mathrm{coordinated}}}}}^{{{{\mathrm{heme}}}}}$ and ${{{\mathrm{IF}}}}_{{{{\mathrm{confined}}}}}^{{{{\mathrm{heme}}}}}$, it maintains the conformation we observed in our cryo-EM structures (*θ*_*Z*_ $\simeq$ 180°). This is reinforced by the fact that the alanine mutations of R77^C^ and R81^C^ in the ${{{\mathrm{IF}}}}_{{{{\mathrm{confined}}}}}^{{{{\mathrm{heme}}}}}$ conformation with heme axially coordinated by the two neighboring histidines do not change *θ*_*Z*_ values (*θ*_*Z*_ $\simeq$ 180°) (Supplementary Fig. 14).

In summary, the heme reorientation mechanism of CydDC may prime heme for directed release toward the periplasmic space or the outer membrane leaflet once the transporter adopts its outward-facing release conformation (Fig. 4b). These results indicate that CydDC operates via a trap-and-flip mechanism similar to that described for primary and secondary active lipid transporters^45–49^.

### Substrate release depends on ATP but not hydrolysis

Next, we aimed to characterize the mechanistic relationship between substrate transport and ATP hydrolysis. Active turnover conditions of wild-type CydDC promote two catalytic states as captured by cryo-EM: ${{{\mathrm{IF}}}}_{{{{\mathrm{bound}}}}}^{{{{\mathrm{heme}}}}}$ and a highly asymmetrical heme-free inward-facing conformation with nucleotide bound to both NBDs $\left( {{{{\mathrm{IF}}}}_{{{{\mathrm{asym}}}}}^{{{{\mathrm{apo}}}}}} \right)$ (Fig. 2c). A lack of additional conformations suggests that formation of ${{{\mathrm{IF}}}}_{{{{\mathrm{confined}}}}}^{{{{\mathrm{heme}}}}}$ and subsequent translocation of heme to the periplasmic space via outward-facing conformations occurs rapidly and features short-lived intermediate states. To capture further transient conformations during the transport cycle, we performed an additional turnover cryo-EM experiment using CydDC^E500Q^ (Figs. 1d and 2c). Owing to the lack of heme in the characterized binding site despite its presence in the sample, we assign this fully occluded conformation as the prehydrolysis return state of CydDC $\left( {{{{\mathrm{Occ}}}}_{{{{\mathrm{return}}}}}^{{{{\mathrm{apo}}}}}} \right)$ following substrate release (Fig. 3a and Supplementary Fig. 11). In contrast to the turnover dataset of CydDC^E500Q^, biochemically defined CydDC^E500Q^ cryo-EM samples revealed conformational heterogeneity reflected by structures of ${{{\mathrm{IF}}}}_{{{{\mathrm{as}}}}\;{{{\mathrm{isolated}}}}}^{{{{\mathrm{apo}}}}}$, ${{{\mathrm{IF}}}}_{{{{\mathrm{confined}}}}}^{{{{\mathrm{heme}}}}}$ and ${{{\mathrm{Occ}}}}_{{{{\mathrm{return}}}}}^{{{{\mathrm{apo}}}}}$ states (Fig. 2c). In the presence of heme and ATP (turnover conditions) this heterogeneity becomes resolved by fully driving CydDC^E500Q^ into the ${{{\mathrm{Occ}}}}_{{{{\mathrm{return}}}}}^{{{{\mathrm{apo}}}}}$ state and trapping it (Fig. 2c). As this effect is not achievable by the addition of heme alone and given the substantially diminished ATPase activity of the CydDC^E500Q^ mutant, we conclude that ATP binding but not its hydrolysis is required for the transition from ${{{\mathrm{IF}}}}_{{{{\mathrm{confined}}}}}^{{{{\mathrm{heme}}}}}$ to ${{{\mathrm{Occ}}}}_{{{{\mathrm{return}}}}}^{{{{\mathrm{apo}}}}}$ state via outward-facing heme-release states (Fig. 1d).

### Conformational coupling between TMHs and NBDs

**Fig. 5:**
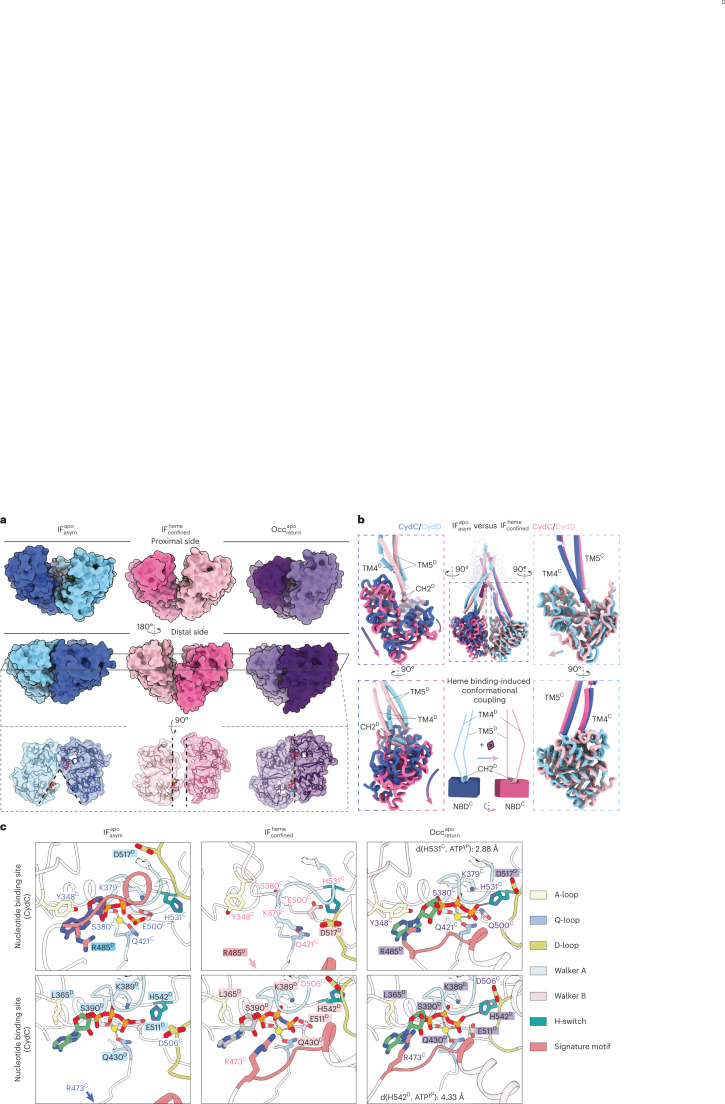
Signal transduction between TMH and NB domains. **a**, Surface representation of NBD–NBD interactions in proximal and distal view. Cross-section top view on the level of the NBS shows distinctly different NBD conformations. ${{{\mathrm{IF}}}}_{{{{\mathrm{asym}}}}}^{{{{\mathrm{apo}}}}}$, blue; ${{{\mathrm{IF}}}}_{{{{\mathrm{confined}}}}}^{{{{\mathrm{heme}}}}}$, magenta; ${{{\mathrm{Occ}}}}_{{{{\mathrm{return}}}}}^{{{{\mathrm{apo}}}}}$, purple. **b**, Mechanism of conformational coupling between TMH and NB domains. Occlusion of the lateral substrate gate induces conformational changes of TMs 4 and 5 of CydD causing a rotational movement of NBD^C^ via coupling helix 2 (CH2^D^). No conformational coupling between TMs of CydC and NBD^D^ is observable. Dark blue, CydC; light blue, CydD; dark pink, CydC; light pink, CydD. **c**, Close-up view of nucleotide-binding pockets in different conformational states and nucleotide occupancies. Conserved structural motifs of the nucleotide-binding and hydrolysis sites are highlighted. ATP, green; ADP, purple; AMP-PNP, gray; phosphate, orange; Mg^2+^, yellow.

The transport of heme involves changes in the inter- and intrasubunit symmetry of CydDC. The ${{{\mathrm{IF}}}}_{{{{\mathrm{asym}}}}}^{{{{\mathrm{apo}}}}}$ conformation features the greatest structural asymmetry (Cα r.m.s.d. 5.4 Å) (Supplementary Fig. 10). Loss of symmetry between CydD and CydC in the ${{{\mathrm{IF}}}}_{{{{\mathrm{asym}}}}}^{{{{\mathrm{apo}}}}}$ state is caused by the rotational movement of each subunit along the transporter’s central axis perpendicular to the membrane plane (Supplementary Video 7). In this wedge-like conformation, the distal side (relative to the heme-binding domain) of CydDC moves closer together than the proximal half whereby ABCα^C^ and ABCβ^D^ form a semi-interlocked NBD dimer (Fig. 5a and Supplementary Video 7). This rearrangement increases the distance between the analogous NBDs of the proximal side, and dilates the heme-accessible membrane cavity (Figs. 3a and 5a). In CydDC, TM4^D^ has a pivotal function during the transition from ${{{\mathrm{IF}}}}_{{{{\mathrm{asym}}}}}^{{{{\mathrm{apo}}}}}$ via ${{{\mathrm{IF}}}}_{{{{\mathrm{bound}}}}}^{{{{\mathrm{heme}}}}}$ to ${{{\mathrm{IF}}}}_{{{{\mathrm{confined}}}}}^{{{{\mathrm{heme}}}}}$ state. As described above, the membrane-integrated segment of TM4^D^ strongly contributes to sealing the bound heme molecule from the membrane environment (Fig. 3, Extended Data Fig. 4 and Supplementary Fig. 11). Beyond this function, we found that movement and rearrangement of the cytoplasmic segment of TM4^D^ is associated with a 25° rotation of the horizontal coupling helix 2 (CH2^D^) connecting TM5^D^ and TM4^D^ (Fig. 5b). The contact of CH2^D^ with NBD^C^ then establishes a conformational coupling that leads to an almost parallel alignment of the two NBDs, which remain separated by roughly 6–8 Å until ATP is bound to the catalytic NBD of CydC (Fig. 5a,b and Supplementary Video 8).

### CydDC features a unique nucleotide exchange mechanism

**Extended Data Fig. 7:**
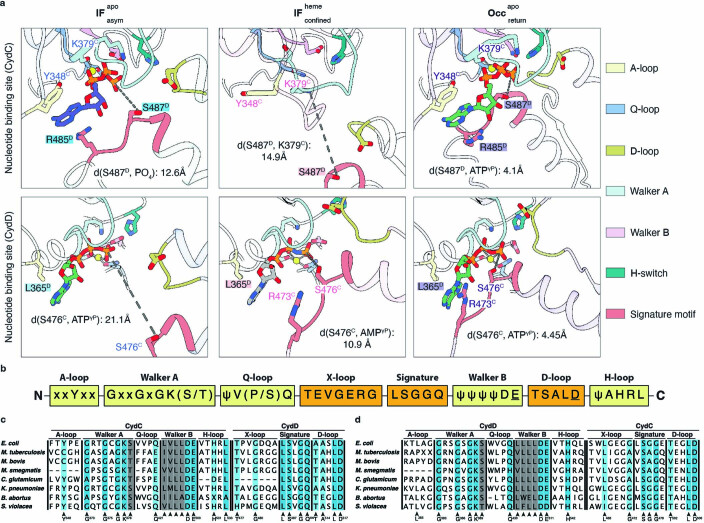
Conformational variety of nucleotide binding sites. **(a)** Top panel: closeup view of NBS^C^ conformations in the ${{{\mathbf{Occ}}}}_{{{{\mathrm{return}}}}}^{{{{\mathrm{apo}}}}}$ (ATP), ${{{\mathbf{IF}}}}_{{{{\mathrm{asym}}}}}^{{{{\mathrm{apo}}}}}$ (ADP + Pi), and ${{{\mathbf{IF}}}}_{{{{\mathrm{confined}}}}}^{{{{\mathrm{heme}}}}}$ (apo) states. Distances between the γ-phosphate and S487^D^ (signature loop), and between K379^C^ of the Walker A domain and S487^D^ are indicated by dashed lines. Bottom panel**:** closeup view of NBS^D^ conformations in the ${{{\mathbf{Occ}}}}_{{{{\mathrm{return}}}}}^{{{{\mathrm{apo}}}}}$ (ATP), ${{{\mathbf{IF}}}}_{{{{\mathrm{asym}}}}}^{{{{\mathrm{apo}}}}}$ (ATP), and ${{{\mathbf{IF}}}}_{{{{\mathrm{confined}}}}}^{{{{\mathrm{heme}}}}}$ (AMP-PNP) states. Distances between the γ-phosphates of ATP/AMP-PNP and S476^C^ of the signature loop are indicated by dashed lines. Conserved structural motifs of nucleotide binding and hydrolysis sites are highlighted. ATP, green; ADP, purple; AMP-PNP, gray; phosphate, orange; Mg^2+^, yellow**. (b-d)** Sequence alignments of the CydD and CydC NBDs from *E. coli*, *M. tb*, *M. smegmatis* and *B. subtilis* revealing the highly conserved motifs for ATP binding and hydrolysis. Highly conserved residues are highlighted in blue. Conserved amino acid groups are highlighted in gray.

In ${{{\mathrm{IF}}}}_{{{{\mathrm{asym}}}}}^{{{{\mathrm{apo}}}}}$, we discovered a previously unknown mode of interaction between the CydD signature loop and nucleotide bound to the canonical nucleotide-binding site (NBS) of CydC (Fig. 5c). During the asymmetrical interaction of the two NBDs on the distal side, the nucleotide is bound to NBS^C^ and its adenine base is coordinated by Y348^C^ of the A-loop via a π–π interaction on one side, and by a π–cation interaction via R485^D^ of the signature loop from CydD on the opposite side. However, in contrast to previously described fully interlocked NBD dimers^47,50^, the signature loop is separated from the γ-phosphate of the nucleotide by 12.6 Å (Fig. 5c and Extended Data Fig. 7a). The degenerate NBS^D^ has a similar geometry in the ${{{\mathrm{IF}}}}_{{{{\mathrm{asym}}}}}^{{{{\mathrm{apo}}}}}$ state, with the exception that adenine does not participate in π–π stacking and π–cation interactions. This is because of the uncharged L365^D^ residue at the analogous position of Y348^C^ in the Walker A motif of CydD, and the fact that the signature loop of CydC is placed at a distance of 20 Å to NBS^D^ (Extended Data Fig. 7a).

A peculiar and thus far unreported NBS conformation was identified in the ${{{\mathrm{IF}}}}_{{{{\mathrm{confined}}}}}^{{{{\mathrm{heme}}}}}$ state. The conformational coupling between substrate occlusion and signal transduction (${{{\mathrm{IF}}}}_{{{{\mathrm{asym}}}}}^{{{{\mathrm{apo}}}}}$ to ${{{\mathrm{IF}}}}_{{{{\mathrm{confined}}}}}^{{{{\mathrm{heme}}}}}$) to the NBDs results in the collapse of the NBS^C^ region and the migration of the Walker A motif into the space originally occupied by the nucleotide phosphate groups (Fig. 5c and Supplementary Video 9). During this process, the conformation of NBS^D^ remains mostly unchanged while the signature loop of CydC moves closer toward the nucleotide, although still separated from it by 10 Å. It is noteworthy that in none of the determined ${{{\mathrm{IF}}}}_{{{{\mathrm{confined}}}}}^{{{{\mathrm{heme}}}}}$ structures, we were able to observe nucleotide bound to NBS^C^, even in conditions where nucleotide had been added. In contrast, in all cases where externally added nucleotides were present in the sample, a clear density was identified at NBS^D^. This observation suggests that the collapsed conformation of NBS^C^ lacks affinity for ATP or any other tested nucleotide.

**Fig. 6:**
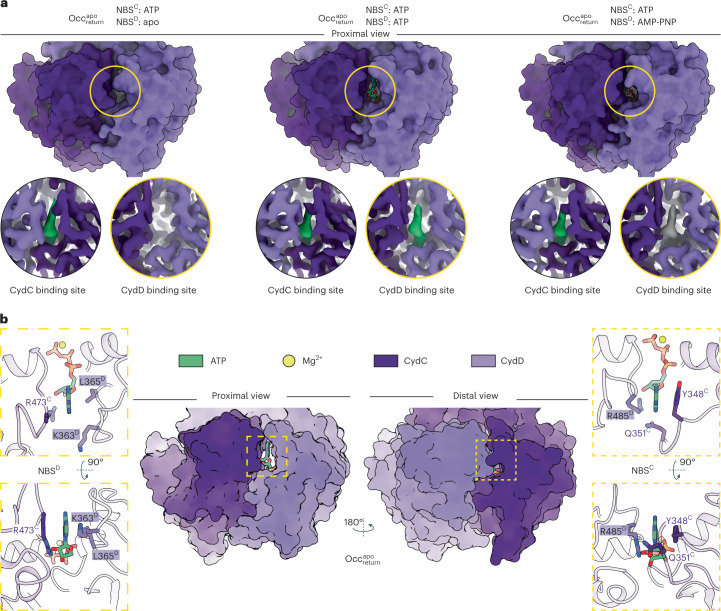
Local environment and accessibility of NBS^D^ and NBS^C^. **a**, The top panel shows a proximal view of tightly interlocked NBDs in the ${{{\mathrm{Occ}}}}_{{{{\mathrm{return}}}}}^{{{{\mathrm{apo}}}}}$ state from CydDC^E500Q^ (datasets 18–20). The bottom panel shows close-up views of the NBS regions of CydD and CydC displaying sample condition specific differences in nucleotide occupancy. ATP densities are shown in green, AMP-PNP densities are shown in gray. Densities of CydC and CydD are shown in dark and light purple, respectively. Bound ATP at NBS^C^ is occluded by tight interactions between the CydC A-loop and the ABCα region of CydD in adjacency of the adenine group. The corresponding loops at NBS^D^ are separated widely and expose ATP bound at this site to the solvent. **b**, The center panel shows a surface model of the interlocked NBD dimer in the ${{{\mathrm{Occ}}}}_{{{{\mathrm{return}}}}}^{{{{\mathrm{apo}}}}}$ state from the turnover dataset of CydDC^E500Q^. The left panel shows a close-up view of ATP bound to NBS^D^ in top and side view orientation. Residues forming the local protein environment close to the adenine head group are shown. The right panel shows a close-up view of ATP bound to NBS^C^ in top and side view orientation. Residues forming the local protein environment close to the adenine head group are shown. ATP, green; Mg^2+^, yellow; CydC, dark purple; CydD, light purple.

Sequence analysis of critical structural motifs required for ATP hydrolysis did not reveal known mutations that would abolish ATP hydrolysis by NBD^D^ (Extended Data Fig. 7b–d). However, the tightly interlocked conformation of ${{{\mathrm{Occ}}}}_{{{{\mathrm{return}}}}}^{{{{\mathrm{apo}}}}}$ provides structural insights into differences between NBDs that may explain hydrolysis deficiency of NBD^D^ (Figs. 5c and 6, Extended Data Fig. 7a and Supplementary Fig. 15). The primary difference between ATP-bound NBD^D^ and NBD^C^ of this prehydrolysis state is the respective distance between the switch histidine and the γ-phosphate of ATP. Whereas H531^C^ is in close distance (2.9 Å) to act as a catalytic acid or base during hydrolysis, H542^D^ is positioned in a less favorable distance of 4.4 Å to participate in polar interactions (Fig. 5c). Here, it is important to mention that when we determined the ${{{\mathrm{Occ}}}}_{{{{\mathrm{return}}}}}^{{{{\mathrm{apo}}}}}$ state structure from a sample that did not include exogenously added ATP or AMP-PNP, we observed lack of bound nucleotide at NBS^D^ but not at NBS^C^ (Figs. 2c and 6a). Loss of the NBS^D^ nucleotide might be possible by the presence of a solvent accessible cavity that remains open toward NBS^D^ even with interlocked NBDs in this conformation, whereas the tight interaction between the A-loop of CydC and ABCα of CydD forms an enclosed inner environment for ATP around NBS^C^ (Fig. 6b and Supplementary Fig. 15). Given the hydrolysis deficiency of CydDC^E500Q^ and the associated conformational differences observed between turnover datasets of the wild-type and CydDC^E500Q^ variants, we assign the consistently copurified nucleotide bound to NBS^C^ as ATP.

### Modeling of outward-facing substrate-release conformations

**Extended Data Fig. 8:**
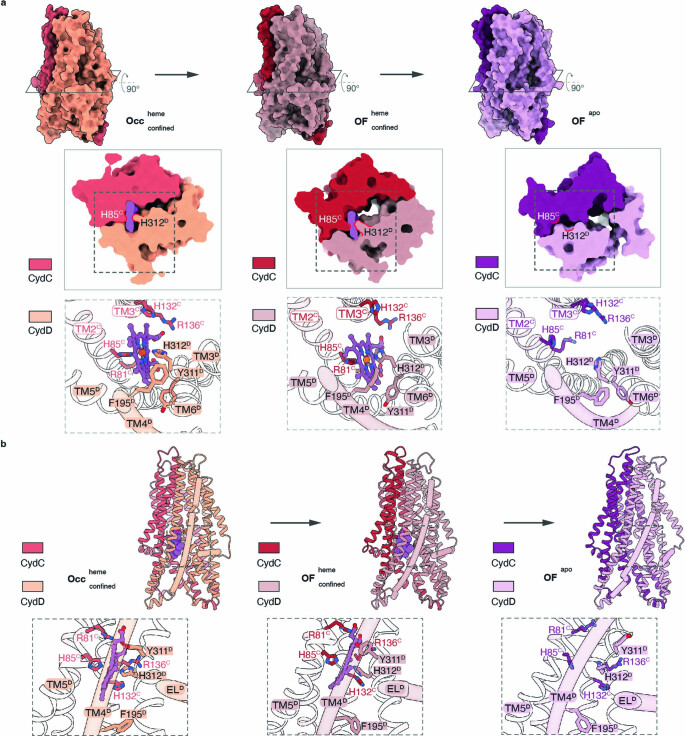
Simulated outward-facing (OF) conformations of CydDC. **(a)** Closeup views show residues involved in heme binding. Surface model cross-sections illustrate the changing shape and size of the internal cavity in different conformational states. Surface model side views highlight the change in overall shape of the TMH region in different conformational states. **(b)** Closeup side views show changing orientations of TMs and heme-binding residues during conformational transitions of the predicted outward-facing states. Heme is shown as purple ball-and-stick model. EL^D^ and TM4^D^ are shown as tubes.

To complement our cryo-EM data, we modeled important but experimentally inaccessible short-lived conformational states during the transition from ${{{\mathrm{IF}}}}_{{{{\mathrm{confined}}}}}^{{{{\mathrm{heme}}}}}$ to ${{{\mathrm{Occ}}}}_{{{{\mathrm{return}}}}}^{{{{\mathrm{apo}}}}}$. Our simulation-based modeling allowed us to construct three transient CydDC conformations representing distinct states of the transport cycle (Extended Data Fig. 8). The ${{{\mathrm{Occ}}}}_{{{{\mathrm{confined}}}}}^{{{{\mathrm{heme}}}}}$ state represents heme-loaded CydDC upon ATP binding with tightly interlocked NBDs. To obtain this conformation, we performed an MD simulation using the slow-growth procedure. After generating the initial ${{{\mathrm{Occ}}}}_{{{{\mathrm{confined}}}}}^{{{{\mathrm{heme}}}}}$ model, we performed 300 ns of simulation and analyzed the structural stability by r.m.s.d. evolution and conformation dynamics. Our final model shows that the transition from ${{{\mathrm{IF}}}}_{{{{\mathrm{confined}}}}}^{{{{\mathrm{heme}}}}}$ to ${{{\mathrm{Occ}}}}_{{{{\mathrm{confined}}}}}^{{{{\mathrm{heme}}}}}$ entails concentered movements of TM3^C^ and TM4^D^. This seals the cytoplasmic gate from solvent access and results in a pseudo-symmetric heterodimer conformation (Extended Data Fig. 8, Supplementary Fig. 16 and Supplementary Video 10).

**Extended Data Fig. 9:**
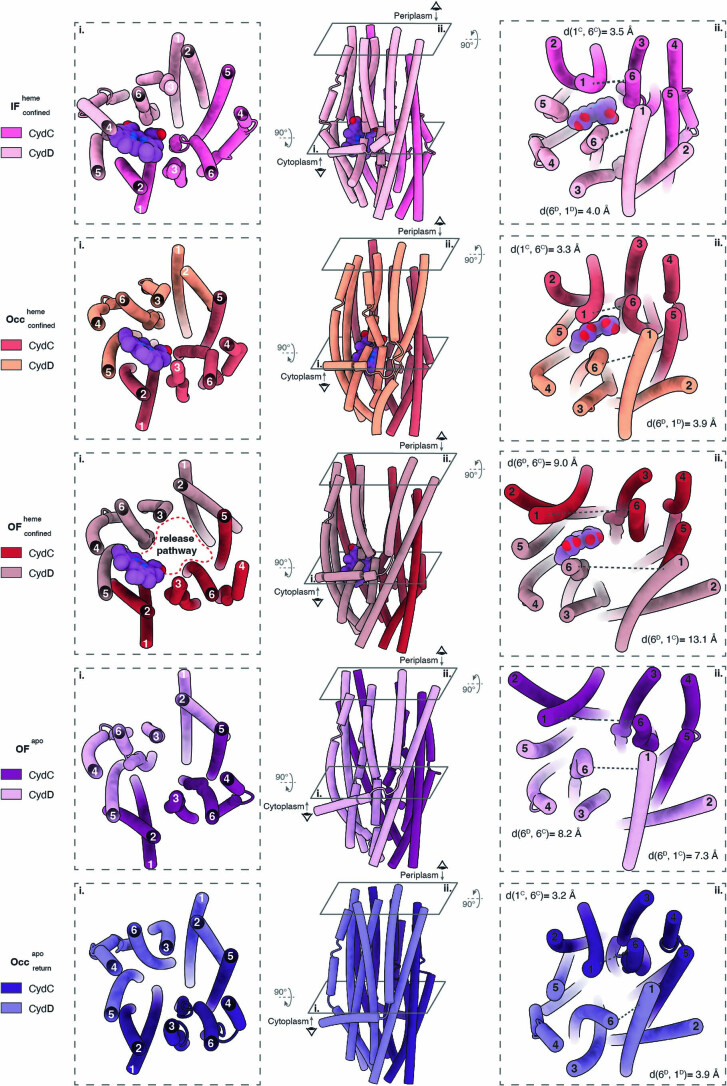
Transmembrane helix rearrangement during the proposed heme release mechanism. Center: side views of CydDC TMH regions during heme confinement and release toward the periplasm. Left: cross-section views of TMH conformations at the level of the heme-binding site. Right: cross-section views of TMH conformations at the level of the periplasmic gate. Distances between TMs 1 and 6 of CydD and CydC (critical for gate opening and closing) are indicated. Periplasmic loop regions are omitted for clarity.

We obtained two outward-open models (${{{\mathrm{OF}}}}_{{{{\mathrm{confined}}}}}^{{{{\mathrm{heme}}}}}$ and ${{{\mathrm{OF}}}}_{}^{{{{\mathrm{apo}}}}}$) by performing steered MD simulations on the two periplasmic halves of CydDC. Having generated initial models, we checked the stability and the degree of opening after 300 ns of unrestrained simulations (Supplementary Fig. 16). Our outward-facing models suggest that heme might be released in a sequential process. First, the periplasmic gate formed by all 12 TMs is opened via separation of two lobes composed of TMs 1–2 of CydC and 4–6 of CydD, and of TMs 1–2 of CydD and 4–6 of CydC (${{{\mathrm{OF}}}}_{{{{\mathrm{confined}}}}}^{{{{\mathrm{heme}}}}}$) (Extended Data Fig. 9 and Supplementary Video 11). During periplasmic gate opening we found that the overall interaction of heme with the residues in the binding site remained unchanged. Even when we performed steered MD simulations on the ${{{\mathrm{Occ}}}}_{{{{\mathrm{confined}}}}}^{{{{\mathrm{heme}}}}}$ conformation with heme not covalently bound to the coordinating histidines, the overall interaction of heme with the residues in the binding site remained unchanged. Accordingly, we conclude that substrate release is initiated by retraction of H312 of TM6^D^ and H85 of TM2^C^ (Extended Data Figs. 8 and 9 and Supplementary Video 12). These events most likely change the local affinity and trigger the release toward the periplasmic side of the membrane. However, the release process time scale is most likely much longer than our submicrosecond simulations, as we do not see any release event in our simulations. The postrelease ${{{\mathrm{OF}}}}_{}^{{{{\mathrm{apo}}}}}$ conformation features a narrower periplasmic gate than ${{{\mathrm{OF}}}}_{{{{\mathrm{confined}}}}}^{{{{\mathrm{heme}}}}}$ during the MD simulations (Supplementary Fig. 16). With the complete closing of the periplasmic gate, the transporter adopts its prehydrolysis ${{{\mathrm{Occ}}}}_{{{{\mathrm{return}}}}}^{{{{\mathrm{apo}}}}}$ conformation as observed in the cryo-EM structures of CydDC^E500Q^ (Fig. 2c and Extended Data Fig. 9). In this state, TM6^D^ and TM2^C^ already adopt the ${{{\mathrm{IF}}}}_{{{{\mathrm{asym}}}}}^{{{{\mathrm{apo}}}}}$ conformation, which would ensure rapid binding of another heme molecule after ATP hydrolysis and lateral opening of the substrate entry gate (Extended Data Figs. 8 and 9 and Supplementary Video 13).

## Discussion

The molecular basis of CydDC function has been contested for 30 years. The results from various in vitro and in vivo studies led to conflicting conclusions about its role in bacterial physiology, and its function as a heme transporter was previously ruled out^21,26,27^. In this study, we provide evidence that translocation of protoheme (heme *b*) from the cytoplasmic space to the periplasm is indeed a primary function of this ABC transporter, which is essential for the functional maturation of cytochrome *bd* (Supplementary Fig. 17).

**Extended Data Fig. 10:**
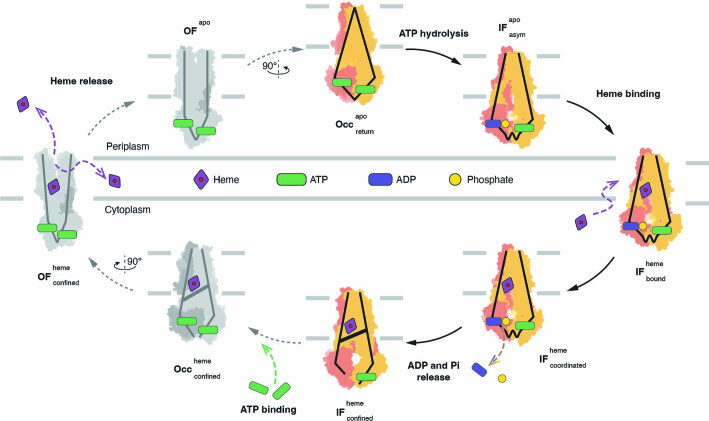
Proposed model of heme transport facilitated by CydDC. Energy of ATP hydrolysis at NBD^C^ converts CydDC from the ${{{\mathbf{Occ}}}}_{{{{\mathrm{return}}}}}^{{{{\mathrm{apo}}}}}$ state to the ${{{\mathbf{IF}}}}_{{{{\mathrm{asym}}}}}^{{{{\mathrm{apo}}}}}$ state. Binding of heme to its dedicated pocket causes closing of the lateral substrate gate and triggers the release of ADP + Pi caused by conformational changes of the nucleotide-binding site of CydC. Release of heme occurs independent of ATP hydrolysis but requires binding of ATP. Separation of TM lobes and rearrangement of residues around the heme-binding pocket cause the release of heme either towards the periplasmic space or to the periplasmic bilayer leaflet of the membrane. Conformations obtained by cryo-EM are shown as yellow and red schematic models. Conformations predicated by MD simulations are shown as light and dark gray models.

Based on our multi-layered approach, we suggest a model for the transport cycle that integrates the results of the complementary methods used in this study (Extended Data Fig. 10 and Supplementary Videos 14 and 15). We assign the ATP-bound ${{{\mathrm{Occ}}}}_{{{{\mathrm{return}}}}}^{{{{\mathrm{apo}}}}}$ state as the starting conformation. We biochemically confirmed that NBD^C^ is essential for the ATPase activity of CydDC (Fig. 1d). In line with this, our structural data indicate that hydrolysis of ATP at this site is required for a mechanochemically induced return to the substrate-free ${{{\mathrm{IF}}}}_{{{{\mathrm{asym}}}}}^{{{{\mathrm{apo}}}}}$ state (Figs. 3a and 5c and Supplementary Fig. 11). By this rationale, we assign the densities at NBS^C^ of this posthydrolysis state obtained from the turnover dataset of CydDC to ADP and Pi (Fig. 5c and Extended Data Fig. 7a). We further conclude that heme binding to the TMH domain and ATP to NBD^C^ occurs sequentially rather than simultaneously, given that the binding and occlusion of heme (${{{\mathrm{IF}}}}_{{{{\mathrm{bound}}}}}^{{{{\mathrm{heme}}}}}$ > ${{{\mathrm{IF}}}}_{{{{\mathrm{coordinated}}}}}^{{{{\mathrm{heme}}}}}$ > ${{{\mathrm{IF}}}}_{{{{\mathrm{confined}}}}}^{{{{\mathrm{heme}}}}}$) is needed to induce dissociation of the distal NBD–NBD interaction, causing NBS^C^ to collapse and release ADP and Pi (Figs. 3–5 and Supplementary Videos 7–9). This latter observation supports our assignment of ADP and Pi in the density maps of ${{{\mathrm{IF}}}}_{{{{\mathrm{bound}}}}}^{{{{\mathrm{heme}}}}}$ and ${{{\mathrm{IF}}}}_{{{{\mathrm{asym}}}}}^{{{{\mathrm{apo}}}}}$ obtained under active turnover conditions of wild-type CydDC with regard to the sequence of conformation changes making up the overall transport cycle. Given the fact that heme binding itself is not sufficient to drive the conformational transition toward the return state conformation but merely results in enrichment of the ${{{\mathrm{IF}}}}_{{{{\mathrm{confined}}}}}^{{{{\mathrm{heme}}}}}$ population, we conclude that binding of ATP to NBS^C^ is essential for the transition to the fully occluded ${{{\mathrm{Occ}}}}_{{{{\mathrm{confined}}}}}^{{{{\mathrm{heme}}}}}$ state. In our MD simulations, this state is followed by the opening of the extracellular gate, the retraction of TM6^C^, including the axial heme ligand H312^D^, to adopt the inward-facing conformation of this helix and the collapsing of the heme-binding site (${{{\mathrm{OF}}}}^{{{{\mathrm{apo}}}}}$) (Extended Data Figs. 8 and 9 and Supplementary Videos 10–13). After release of the heme to the periplasmic space, the extracellular gate closes again, reverting back to the ${{{\mathrm{Occ}}}}_{{{{\mathrm{return}}}}}^{{{{\mathrm{apo}}}}}$ conformation. This is in agreement with the observation that under turnover conditions of CydDC^E500Q^ in which heme and ATP are present, we exclusively captured the occluded return state conformation. Notably, this is the sole analyzed condition, that does not result in a heme-bound conformation of CydDC. A lack of ATP hydrolysis activity of the CydDC^E500Q^ variant traps the transporter in the postsubstrate-release state suggesting that the key role of ATP hydrolysis is to convert CydDC to the ${{{\mathrm{IF}}}}_{{{{\mathrm{asym}}}}}^{{{{\mathrm{apo}}}}}$, as observed in the turnover dataset of CydDC^wt^.

The transitional cycle of asymmetrical, symmetry-approaching and pseudo-symmetrical conformations during heme transport is unique to CydDC and highlights the mechanistic possibilities for the specialized substrate transport facilitated by heterodimeric ABC transporters and their diverse conformational space (Supplementary Fig. 18).

## Methods

### Production of CydDC from *E. coli*

CydDC from *E. coli* was produced in *E. coli* BL21-pLysS (DE3) cells transformed with a pTTQ18 plasmid carrying structural genes of the CydDC heterodimer (*cydD* + *cydC*) and encoding a C-terminal hexahistidine modification at CydC for downstream IMAC purification (p*cydDC*)^29^. Mutant variants CydDC^E500Q^ (p*E500Q*), CydD^E511Q^C (p*E511Q*) and CydDC^H85A^ (p*H85A*) were produced analogously. Further details are found in sequence data provided as Supplementary Information (Supplementary Tables 2 and 3).

For an initial preculture, a volume of 0.1 ml of 50% glycerol stock of each respective strain was added to 200 ml of M9-Carb (50 µg ml^−1^ carbenicillin) growth medium. Cells were incubated at 37 °C while shaking (185 rpm) for 16–20 h. The production culture was started by inoculating 2 l of M9-Carb (50 µg ml^−1^ carbenicillin) growth medium with the preculture adjusted to a starting optical density (OD_600_) of 0.1. Cells were grown while shaking (185 rpm) at 37 °C until an OD_600_ of 0.6 before recombinant production was induced by addition of isopropyl-β-d-thiogalactoside at a final concentration of 0.6 mM. Gene expression was carried out at 30 °C for 16–18 h. After collection, cells were disrupted using a French-press cell disruptor (Thermo Fisher Scientific) via double pass at a pressure of 700–1,000 psi. The cell lysate was centrifuged at 5,000*g* at 4 °C for 30 min. Subsequently, the low-velocity supernatant was centrifuged at 220,000*g* at 4 °C for 90 min. Pelleted membranes were resuspended and stored at a protein concentration of 10 mg ml^−1^ in a storage buffer containing 50 mM Tris-HCl (pH 7.4) and 100 mM KCl.

### Purification of CydDC from *E. coli*

Isolated membranes were solubilized with 1% dodecyl-β-d-maltoside (DDM) in a mass ratio of 1 mg detergent per 5 mg of membrane protein for 60 min at 4 °C. CydDC was purified via His_6_-tag affinity chromatography using a Talon (Co-IMAC) affinity matrix (Takara Bio Inc.). For column conditioning and washing step I (10 column volumes (CV)) a buffer containing 50 mM Tris-HCl (pH 8), 500 mM KCl, 10% (v/v) glycerol, 20 mM imidazole, and 0.02% DDM was used. For samples intended to be used for cryo-EM studies, the resin was additionally washed with 10 CV of washing buffer II containing 50 mM Tris-HCl (pH 8), 500 mM KCl, 10% (v/v) glycerol, 20 mM imidazole and 0.05% lauryl maltose neopentyl glycol (LMNG) to perform detergent exchange from DDM to LMNG. For step elution (5 × 1 CV), buffers I and II were adjusted to a final imidazole concentration of 300 mM, respectively. For sample polishing, size-exclusion chromatography was performed using a Superdex 200 10/300 Increase column. The size-exclusion chromatography running buffer contained 20 mM MES (pH 6), 50 mM KCl and either 0.02% DDM or 0.001% LMNG. All steps of the purification process were carried out at 4 °C.

### Growth-complementation studies

The bacterial growth assays were carried out as described previously^31^. Precultures of *E. coli* MB43 and MB43Δ*cydDC* (transformed with empty pET17 control vector or with plasmids encoding one of the cytochrome *bd* variants and/or CydDC) were incubated in Luria Bertani (LB) medium with 100 μg ml^−1^ ampicillin overnight at 37 °C while shaking at 200 rpm. Bacteria were then diluted to OD_600_ of 0.01 with 200 μl of LB medium containing 100 μg ml^−1^ ampicillin and each sample was subsequently distributed over eight wells (technical replicates) of a 96-well microtiter plate. OD_600_ was measured every 5 min at 37 °C for 20 h (shaking at 200 rpm) in a SpectraMax Plus 384 Microplate reader (Molecular Devices).

### ATP hydrolysis assays

#### Malachite green phosphate assay

ATPase activity of CydDC was determined colorimetrically as described previously^51^. In brief, measurements were performed at a final concentration of 50 nM CydDC (in DDM) in a volume of 25 µl and carried out for 5 min at 37 °C. Reactions were stopped by adding 175 μl of ice cold stopping buffer (20 mM H_2_SO_4_) and stored on ice. For detection, 175 μl of a stopped reaction sample was transferred into a 96-well microtiter plate and incubated for 8 min with 50 μl of malachite green solution (2.7 mM malachite green chloride, 0.17% Tween, 1.5% Na_2_MoO_4_) at room temperature. The absorbance change at 620 nm was measured by a SpectraMax M2 Microplate Reader (Molecular Devices). For heme titration experiments a constant ATP concentration of 1 mM was used. ATP titration experiments were performed at a constant heme concentration of 0.3 µM. Substate screening experiments were performed using following final concentrations of the respective reaction components: ATP (1 mM), ferrous heme (0.5 µM), ferric heme (0.5 µM), protoporphyrin IX (0.5 µM), FeCl_3_ (0.5 µM), GSH (1 mM), glutathione disulfide (1 mM), l-Cys (0.5 mM), cystine (1 µM), orthovanadate (1 mM) and AMP-PNP (1 mM). All measurements were performed in a reaction buffer containing 20 mM Tris-HCl (pH 7.0), 50 mM KCl, 0.02% DDM and 3 mM MgCl_2_. Samples without substrate were used as negative control and subtracted as background. All data are presented as mean ± s.d. (*n* = 3). Statistical significance was analyzed via paired two-tailed Student’s *t*-tests using the GraphPad *t*-Test calculator (https://www.graphpad.com/quickcalcs/ttest1/).

#### Enzyme-coupled ATPase assay

The pyruvate kinase and lactate dehydrogenase coupled ATP hydrolysis assay was performed as described previously^52^. In brief, a volume of 100 μl of activity buffer (20 mM Tris-HCl (pH 7.0), 50 mM KCl, 4 mM MgCl_2_, 350 μM PEP, 350 μM ATP, 350 μM NADH, 0.02% DDM) was mixed with 2.5 μl of pyruvate kinase and lactate dehydrogenase enzyme solution (Sigma Aldrich, P0294) and incubated for 5 min at 37 °C in a 96-well microtiter plate. Subsequently, putative substrate candidates and CydDC (0.6 µM final concentration) were added. Kinetic turnover was monitored at 340 nm for 45 min every 10 s using the SpectraMax M2 Microplate Reader (Molecular Devices). All data are presented as mean ± s.d. (*n* = 3). Statistical significance was analyzed via paired two-tailed Student’s *t*-tests using the GraphPad *t*-Test calculator (https://www.graphpad.com/quickcalcs/ttest1/).

### Determination of thermal stability by microscale thermophoresis

Thermal stabilities of purified CydDC variants were investigated with a Prometheus NT.48 instrument (Nanotemper). Excitation at 280 nm (20 nm bandwidth) was set to a power of 10% yielding emission intensities of 6,000 to 25,000 at 333–380 nm. A temperature ramp of 1 °C min^−1^ between 20 and 95 °C was applied in all experiments. Measurements were performed at 2 µM CydDC concentration. Heme was added in equimolar concentrations to CydDC. Unfolding transitions were monitored from changes in the emission of tryptophan and tyrosine fluorescence at 350 and 330 nm, respectively. Melting temperatures were determined at the inflection points (free energy change Δ*G* is equal to zero) from the raw data (measured in triplicates) after baseline correction and normalization.

### Cryo-EM sample preparation

To collect cryo-EM data of CydDC in different sample conditions, different combinations of CydDC variants, nucleotides, inhibitors and putative substrate molecules were prepared. See Fig. 2c and Supplementary Fig. 7 for details of specific sample conditions. For all nonturnover datasets, the protein concentration was adjusted to approximately 1.5 mg ml^−1^ before other components were added. Heme loading was performed before sample vitrification. Heme was adjusted to a final concentration of 17 µM in all heme containing samples. Nucleotides and inhibitors (ADP, AMP-PNP and ATP-orthovanadate) were used at a final concentration of 1 mM (+2 mM MgCl_2_). Putative substrate molecules were screened at final concentrations of 1 mM. Owing to poor aqueous solubility, cystine was adjusted to the highest possible final concentration of 0.2 mM. Samples were incubated for 2 min at room temperature before plunge freezing.

For preparation of turnover samples, the protein concentration was adjusted to 3 mg ml^−1^. Subsequently, heme was added at a final concentration of 34 µM. The sample was then mixed with a freshly prepared ATPase buffer (20 mM MES (pH 6), 50 mM KCl, 0.001% LMNG, 10 mM ATP and 20 mM MgCl_2_) in a ratio of 1:1, incubated for 30 s at 37 °C and immediately subjected to plunge freezing. Identical plunge freezing conditions were applied for all samples: Quantifoil R1.2/1.3 copper grids (mesh 300) were washed in chloroform and subsequently glow discharged with a PELCO easiGlow device at 15 mA for 90 s. A volume of 4 µl of sample was applied to a grid and blotting was performed for 4 s at 4 °C, 100% humidity with nominal blot force 20 immediately before freezing in liquid ethane, using a Vitrobot Mark IV device (Thermo Scientific).

### Cryo-EM image recording

For each cryo-EM sample, a dataset was recorded in Energy-Filtered Transmission Electron Microscopy (EF-TEM) mode using either a Titan Krios G2 or a Krios G3i microscope (Thermo Scientific), both operated at 300 kV. Electron-optical alignments were adjusted with EPU 2.9–2.11 (Thermo Scientific). Images were recorded using automation strategies of EPU 2.9–2.11 in electron counting mode with either a Gatan K2 (installed on Krios G2) or a Gatan K3 (installed on Krios G3i) direct electron detector at a nominal magnification of 105,000, corresponding to a calibrated pixel size of 0.831 and 0.837 Å, respectively. Dose fractionated movies (40 frames) were recorded at an electron flux of approximately 15 e^−^ pixel^−1^ s^−1^ for 2 s, corresponding to a total dose of roughly 40 e^−^/A^2^. Images were recorded between −1.1 and −2.1 µm nominal defocus. CryoSPARC Live v.3.0 was used for real-time cryo-EM data quality assessment.

### Cryo-EM image processing

For each acquired dataset, the same cryo-EM image processing approach was applied: MotionCor2 was used to correct for beam-induced motion and to generate dose-weighted images^53^. Gctf was used to determine the contrast transfer function parameters and perform correction steps^54^. Images with estimated poor resolution (<4 Å) and severe astigmatism (> 400 Å) were removed at this step. Particles were picked by crYOLO and used for all further processing steps^55^. Two-dimensional classification, initial model generation, three-dimensional classification, contrast transfer function refinement, Bayesian polishing, three-dimensional sorting and final map reconstructions were performed using RELION-3.1 (ref. ^56^). Fourier shell correlation curves were generated in RELION-3.1. Local-resolution estimation was performed in RELION-3.1 for all final maps. A schemtatic overview of our processing workflow, and a summary of map qualities are shown in Supplementary Figs. 4–7 and 9.

### Model building and geometry refinement

The first atomic model of CydDC was built de novo into the EM density map of the ${{{\mathrm{IF}}}}_{{{{\mathrm{as}}}}\;{{{\mathrm{isolated}}}}}^{{{{\mathrm{apo}}}}}$ state in Coot (v.0.8). After manual backbone tracing and docking of side chains in the respective map densities real-space refinement in Phenix was performed (v.1.14 and 1.18)^57^. Refinement results were manually inspected and corrected if required. This model was used as a template to build all subsequent atomic models. In total, 17 models were built, refined, inspected and corrected (Supplementary Fig. 8). The finalized models were validated by MolProbity implemented in Phenix. Map-to-model cross validation was performed in Phenix (v.1.14 and 1.18). Fourier shell correlation_0.5_ was used as cutoff to define resolution. A summary of model parameters and the corresponding cryo-EM map statistics is found in Supplementary Table 1. The finalized models were visualized using ChimeraX. Highest resolution models of ${{{\mathrm{IF}}}}_{{{{\mathrm{as}}}}\;{{{\mathrm{isolated}}}}}^{{{{\mathrm{apo}}}}}$, ${{{\mathrm{IF}}}}_{{{{\mathrm{asym}}}}}^{{{{\mathrm{apo}}}}}$, ${{{\mathrm{IF}}}}_{{{{\mathrm{bound}}}}}^{{{{\mathrm{heme}}}}}$, ${{{\mathrm{IF}}}}_{{{{\mathrm{coordinated}}}}}^{{{{\mathrm{heme}}}}}$, ${{{\mathrm{IF}}}}_{{{{\mathrm{confined}}}}}^{{{{\mathrm{heme}}}}}$ and ${{{\mathrm{Occ}}}}_{{{{\mathrm{return}}}}}^{{{{\mathrm{apo}}}}}$ states were used as starting structures for MD simulations.

### Tunnels and interior cavities

Tunnels and cavities were mapped with MOLE 2.5 with a bottleneck radius of 1.2 Å, bottleneck tolerance 3 Å, origin radius 5 Å, surface radius 10 Å, probe radius 5 Å and an interior threshold of 1.1 Å.

### Structural alignments

The overall folds of the CydDC subunits CydC and CydD were compared with each other using the structural alignment program TM-align. Subunits, individual transmembrane regions and NBDs were aligned and respective Cα r.m.s.d. values were calculated.

### MD simulations

The CydDC structures were placed in a heterogenous bilayer composed of POPE (70%), POPG (25%) and CL (5%) using CHARMM-GUI^58^. All systems were hydrated with 150 mM NaCl electrolyte. The all-atom CHARMM36m force field was used for lipids, ions, cofactors and protein with TIP3P water. MD trajectories were analyzed using Visual Molecular Dynamics and MDAnalysis.

All simulations were performed using GROMACS v.2021.2 (ref. ^59^). Starting systems were energy minimized for 5,000 steepest descent steps and equilibrated initially for 500 ps of MD in a canonical (NVT) ensemble and later for 7.5 ns in an isothermal-isobaric (NPT) ensemble under periodic boundary conditions. During equilibration, the restraints on the positions of nonhydrogen protein atoms of initially 4,000 kJ mol^−1^ nm−^2^ were gradually released. Particle-mesh Ewald summation with cubic interpolation and a 0.12-nm grid spacing was used to treat long-range electrostatic interactions. The time step was initially 1 fs, and was increased to 2 fs during the NPT equilibration. The LINCS algorithm was used to fix all bond lengths. Constant temperature was established with a Berendsen thermostat, combined with a coupling constant of 1.0 ps. A semi-isotropic Berendsen barostat was used to maintain a pressure of 1 bar. During production runs, the Berendsen thermostat and barostat were replaced by a Nosé–Hoover thermostat and a Parrinello–Rahman barostat. All simulations were performed at 310 K. Analysis was carried out on unconstrained simulations. Simulations with liganded and unliganded heme were performed for 450 ns for each of ${{{\mathrm{IF}}}}_{{{{\mathrm{bound}}}}}^{{{{\mathrm{heme}}}}}$, ${{{\mathrm{IF}}}}_{{{{\mathrm{coordinated}}}}}^{{{{\mathrm{heme}}}}}$ and ${{{\mathrm{IF}}}}_{{{{\mathrm{confined}}}}}^{{{{\mathrm{heme}}}}}$ states.

For heme entry simulations a heterogenous bilayer composed of POPE (70%), POPG (25%) and CL(5%) was built using CHARMM-GUI. The system was hydrated with 150 mM NaCl electrolyte. After 7 ns of equilibration, a production run was performed for 100 ns. Then, a heme molecule (ferrous state) was placed 1 nm away from the membrane. Ten separate MD simulations were initiated with independent initial velocities drawn according to the Boltzmann distribution of the targeted temperature. Each replicate was run for additional 100 ns. In seven out of the ten simulations, the heme molecule had partitioned into the membrane bilayer at the end of 100 ns simulation. The ${{{\mathrm{IF}}}}_{{{{\mathrm{as}}}}\;{{{\mathrm{isolated}}}}}^{{{{\mathrm{apo}}}}}$ CydDC structure was placed in a heterogenous bilayer composed of POPE (70%), POPG (25%) and CL (5%) using CHARMM-GUI. Then, a heme molecule was placed near the lateral opening between TM4^D^ and TM6^D^ in five different positions. After equilibration, a simulation of 100 ns duration was performed for each setup.

We modeled the ${{{\mathrm{Occ}}}}_{{{{\mathrm{confined}}}}}^{{{{\mathrm{heme}}}}}$ state, which represents the heme-loaded conformation of CydDC on ATP binding, with tightly interlocked NBDs. To obtain this conformation, we first ran an MD simulation for the fully occluded state of CydDC without heme (${{{\mathrm{Occ}}}}_{{{{\mathrm{return}}}}}^{{{{\mathrm{apo}}}}}$) for 400 ns to get an equilibrated structure in the membrane bilayer. Then, the end structure of this simulation was aligned with the ${{{\mathrm{IF}}}}_{{{{\mathrm{confined}}}}}^{{{{\mathrm{heme}}}}}$ conformation and a virtual heme molecule was placed in the approximate position of the binding site taken from the aligned ${{{\mathrm{IF}}}}_{{{{\mathrm{confined}}}}}^{{{{\mathrm{heme}}}}}$ conformation. In the next step, three independent simulations were carried out to generate the ${{{\mathrm{Occ}}}}_{{{{\mathrm{confined}}}}}^{{{{\mathrm{heme}}}}}$ conformation by gradually turning on the interactions of the virtual heme with the protein and the rest of the system. Increasing a lambda parameter scaling these interactions slowly from 0 to 1, over 1 ns with a 0.5 fs time step, results in the insertion of heme by slow growth. This method allows for smooth adaptation of amino acids in the binding site to the presence of heme. Extra position restraints were added to the C-alpha atoms of the NBDs to stabilize the conformation of protein during the slow-growth process. After the slow-growth heme insertion, we added the axial ligation bonds between heme and H85^C^ and H312^D^ and energy minimized the system. Then, we performed 300 ns of simulation. Structural stability during simulations was checked by r.m.s.d. evolution and conformation dynamics.

To obtain structural models of the two elusive outward-facing (${{{\mathrm{OF}}}}_{{{{\mathrm{confined}}}}}^{{{{\mathrm{heme}}}}}$ and ${{{\mathrm{OF}}}}^{{{{\mathrm{apo}}}}}$) states, we performed steered MD simulations starting from ${{{\mathrm{Occ}}}}_{{{{\mathrm{confined}}}}}^{{{{\mathrm{heme}}}}}$ and ${{{\mathrm{Occ}}}}_{{{{\mathrm{return}}}}}^{{{{\mathrm{apo}}}}}$, respectively. A bias was applied on the distance between the center of mass of the two periplasmic halves of CydDC. The periplasmic halves are defined as (1) TM 1–2 of CydC and TM 3–6 of CydD and (2) TM 1–2 of CydD and TM 3–6 of CydC. The initial value of the distance was 19 Å and the target value was 28 Å (based on the outward-open structure of TmrAB Protein Data Bank ID 6RAJ). The steered MD was performed in PLUMED-patched Gromacs with an approximate velocity of 0.01 nm ns^−1^. The steered MD simulation was performed for 100 ns. After the simulation and full opening of the periplasmic side, we ran a further 50 ns of restrained simulation that allowed the lipid molecules around the periplasmic site to equilibrate around the new conformation. Finally, we checked the stability and the degree of opening after 300 ns of unrestrained simulations (Supplementary Fig. 16).

A list of all the simulations performed in this study is presented in Supplementary Table 4 in the Supplementary Information with their corresponding descriptions and time lengths.

### Preparation of membrane fractions for heme spectra and oxygen consumption assays

Membrane fractions were isolated as described previously^60^. In brief, *E. coli* strains were grown in 800 ml of LB medium (2-l baffled flasks) from a starting OD_600_ of 0.01 until the late exponential phase. After washing with phosphate buffer saline, batches of cells (5 g) were resuspended in a buffer composed of 50 mM MOPS (pH 7), 100 mM NaCl and cOmplete protease inhibitor (Roche) in a ratio of 5:1. Cells were disrupted using a Stansted homogenizer at 1.2 kbar. Cell debris was removed by centrifugation at 9,500*g* for 20 min at 4 °C. Membrane fractions were collected by ultracentrifugation at 250,000*g* for 75 min at 4 °C. Membranes were resuspended in the above buffer containing 0.025% DDM, and used for downstream oxygen consumption activity assays and spectroscopic analysis of heme cofactors.

### UV-vis absorption spectroscopy for heme identification

The heme composition of membrane fractions was analyzed by reduced-minus oxidized UV-vis spectra based on Goojani et al.^31^. Membrane fractions were diluted to a protein concentration of 2.6 mg ml^−1^ in a buffer composed of 10 mM Tris (pH 7.4) and 16 mM sodium cholate. Samples were first oxidized with 100 μM potassium ferricyanide and a spectrum was recorded at room temperature using a Varian Cary 50 UV-vis Spectrophotometer. Subsequently, a few grains of solid sodium hydrosulfite were dissolved in the sample to measure the spectrum in the fully reduced state. The difference spectrum (reduced–oxidized) was calculated using OriginLab Pro v.9.5 (Additive GmbH).

### Oxygen consumption measurements

The oxygen consumption activity of membrane fractions was measured using a Clark-type electrode based on Goojani et al.^31^. The membrane fractions were adjusted to a concentration of 0.01–0.03 mg ml^−1^ using a buffer composed of 50 mM MOPS (pH 7.0), 100 mM NaCl and 0.025% DDM. Subsequently, the membrane fractions were preincubated with either aurachin D (final concentration 400 nM) or with dimethylsulfoxide as control for 3 min in the electrode chamber. Ubiquinone-1 and dithiothreitol (DTT) were preincubated separately for 3 min and then injected into the electrode chamber to start the reaction (final concentration of 200 μM ubiquinone-1 and 10 mM DTT). The reaction rate was determined for the period between 90 and 150 s after addition of the substrate mixture. Statistical significance was analyzed via paired two-tailed Student’s *t*-tests using the GraphPad *t*-test calculator (https://www.graphpad.com/quickcalcs/ttest1/).

### Multiple sequence alignments

Multiple sequence alignments of CydD and CydC from *E. coli* (strain K12), *Mycobacterium tuberculosis* (strain ATCC 25618/H37Rv), *Mycobacterium smegmatis* MC2-155, *Mycobacterium bovis* (strain ATCC BAA-935/AF2122/97), *Corynebacterium glutamicum*, *Brucella abortus biovar*, *Shewanella violacea* (strain JCM 10179/CIP 106290/LMG 19151/DSS12), *Geobacillus stearothermophilus* and *Klebsiella pneumoniae* were performed using Clustal Omega and visualized using Jalview v.2.11.2.0.

### Reporting summary

Further information on research design is available in the Nature Portfolio Reporting Summary linked to this article.

## Online content

Any methods, additional references, Nature Portfolio reporting summaries, source data, extended data, supplementary information, acknowledgements, peer review information; details of author contributions and competing interests; and statements of data and code availability are available at 10.1038/s41589-023-01314-5.

## Supplementary information

Supplementary Information Supplementary Tables 1–4 and Supplementary Figs. 1–18.

Reporting Summary

Supplementary Video 1Conformational transition from ${{{\mathrm{IF}}}}_{{{{\mathrm{asym}}}}}^{{{{\mathrm{apo}}}}}$ to ${{{\mathrm{IF}}}}_{{{{\mathrm{bound}}}}}^{{{{\mathrm{heme}}}}}$ induced by the initial binding of heme.

Supplementary Video 2Conformational transition from ${{{\mathrm{IF}}}}_{{{{\mathrm{bound}}}}}^{{{{\mathrm{heme}}}}}$ to ${{{\mathrm{IF}}}}_{{{{\mathrm{coordinated}}}}}^{{{{\mathrm{heme}}}}}$ by rearrangement of the TMH region.

Supplementary Video 3Close-up view on the bilateral axial coordination of the heme iron during the transition from ${{{\mathrm{IF}}}}_{{{{\mathrm{bound}}}}}^{{{{\mathrm{heme}}}}}$ to ${{{\mathrm{IF}}}}_{{{{\mathrm{coordinated}}}}}^{{{{\mathrm{heme}}}}}$.

Supplementary Video 4Conformational transition from ${{{\mathrm{IF}}}}_{{{{\mathrm{coordinated}}}}}^{{{{\mathrm{heme}}}}}$ to ${{{\mathrm{IF}}}}_{{{{\mathrm{confined}}}}}^{{{{\mathrm{heme}}}}}$: overall structural changes.

Supplementary Video 5Conformational transition from ${{{\mathrm{IF}}}}_{{{{\mathrm{coordinated}}}}}^{{{{\mathrm{heme}}}}}$ to ${{{\mathrm{IF}}}}_{{{{\mathrm{confined}}}}}^{{{{\mathrm{heme}}}}}$: close-up view of the gate-closing dynamics.

Supplementary Video 6Conformational transition from ${{{\mathrm{IF}}}}_{{{{\mathrm{coordinated}}}}}^{{{{\mathrm{heme}}}}}$ to ${{{\mathrm{IF}}}}_{{{{\mathrm{confined}}}}}^{{{{\mathrm{heme}}}}}$: close-up view of changing solvent accessibility to the heme-binding site.

Supplementary Video 7Conformational transition from ${{{\mathrm{Occ}}}}_{{{{\mathrm{return}}}}}^{{{{\mathrm{apo}}}}}$ to ${{{\mathrm{IF}}}}_{{{{\mathrm{asym}}}}}^{{{{\mathrm{apo}}}}}$ induced by the mechanochemical coupling of ATP hydrolysis.

Supplementary Video 8 Signal transduction between transmembrane and NBDs. Conformational coupling causes a rotational movement of NBD^C^ on closing of the lateral substrate entry gate by TM4^D^.

Supplementary Video 9 Conformational changes at the NBSs of CydD and CydC when the lateral membrane gate closes, and conformational coupling to the NBDs.

Supplementary Video 10Predicted conformational transition from ${{{\mathrm{IF}}}}_{{{{\mathrm{confined}}}}}^{{{{\mathrm{heme}}}}}$ to ${{{\mathrm{Occ}}}}_{{{{\mathrm{confined}}}}}^{{{{\mathrm{heme}}}}}$ driven by dimerization of the NBDs.

Supplementary Video 11Predicted conformational transition from ${{{\mathrm{Occ}}}}_{{{{\mathrm{confined}}}}}^{{{{\mathrm{heme}}}}}$ to ${{{\mathrm{OF}}}}_{{{{\mathrm{confined}}}}}^{{{{\mathrm{heme}}}}}$ driven by the separation of the two periplasmic lobes of the TMH domain forming the periplasmic gate.

Supplementary Video 12Predicted conformational transition from ${{{\mathrm{OF}}}}_{{{{\mathrm{confined}}}}}^{{{{\mathrm{heme}}}}}$ to ${{{\mathrm{OF}}}}^{{{{\mathrm{apo}}}}}$ following the release of heme.

Supplementary Video 13Predicted conformational transition from ${{{\mathrm{OF}}}}^{{{{\mathrm{apo}}}}}$ to the prehydrolysis ${{{\mathrm{Occ}}}}_{{{{\mathrm{return}}}}}^{{{{\mathrm{apo}}}}}$ state driven by the closing of the periplasmic membrane gate.

Supplementary Video 14 Overview of key events during heme binding and occlusion.

Supplementary Video 15 Overview of predicted heme-release process.

## Data Availability

Cryo-EM maps are deposited at the Electron Microscopy Data Bank under accession numbers EMD-14636, EMD-14638, EMD-14639, EMD-14640, EMD-14641, EMD-14642, EMD-14643, EMD-14644, EMD-14645, EMD-14646, EMD-14647, EMD-14649, EMD-14652, EMD-14653, EMD-14654, EMD-14655, EMD-14656, EMD-14657, EMD-14659, EMD-14660, EMD-14662, EMD-14663, EMD-14665, EMD-14667, EMD-14668, EMD-14669, EMD-14670, EMD-14671, EMD-14672, EMD-14673, EMD-14674, EMD-14675, EMD-14676, EMD-14684, EMD-14689, EMD-15264 and EMD-15265. Atomic models of CydDC have been deposited to the Protein Data Bank under accession numbers: 7ZD5, 7ZDA, 7ZDB, 7ZDC, 7ZDE, 7ZDF, 7ZDG, 7ZDK, 7ZDL, 7ZDR, 7ZDS, 7ZDT, 7ZDU, 7ZDV, 7ZDW, 7ZE5 and 7ZEC. All other data are presented in the main text or Supplementary Information. Source data are provided with this paper.

## Source data

Source Data Fig. 1 Cell growth data, UV-vis absorbance data, malachite-based ATPase screening and titration data, oxygen consumption rates and statistical source data.
Source Data Extended Data Fig. 1 ATPase activity screening data with l-Cys and GSH used as putative substrates.
Source Data Extended Data Fig. 2 ATPase activity data using malachite-based and enzyme-coupled methodological approaches.
Source Data Extended Data Fig. 3 Malachite-based ATPase data showing turnover rates of wild-type and mutant variants of CydDC.
Source Data Extended Data Fig. 5 Size-exclusion chromatography profiles at 280 and 410 nm to track heme binding to CydDC. Thermal stability values deduced from nanoDSF experiments of wild-type CydDC and the H85A variant.
