# Supplementary material for: Dissecting the conformational complexity and mechanism of a bacterial heme transporter
Source: Nat Chem Biol. 2023 Apr 24;19(8):992–1003. doi: 10.1038/s41589-023-01314-5 (PMC10374445; doi:10.1038/s41589-023-01314-5)
Supplement: Supplementary file 1 — Supplementary Tables 1–4 and Supplementary Figs. 1–18. [file 41589_2023_1314_MOESM1_ESM.pdf]

nature chemical biology

Article

https://doi.org/10.1038/s41589-023-01314-5

Dissecting the conformational complexity and mechanism of a bacterial heme transporter

In the format provided by the authors and unedited

This file includes:

Supplementary Tables 1 - 4

Supplementary Figures 1 - 18

Other Supplementary Material for this manuscript includes the following:

Supplementary Videos 1 - 15

Supplementary Table 1 – Cryo-EM and model data statistics.

	**Dataset 1** IF(apo/as isolated)	**Dataset 1** IF(heme/confined)	**Dataset 2** IF(apo/asym)	**Dataset 2** IF(heme/bound)	**Dataset 3** IF(apo/as isolated)	**Dataset 3** IF(heme/confined)	**Dataset 4** IF(apo/as isolated)	**Dataset 4** IF(heme/confined)	**Dataset 5** IF(heme/confined)
**Data collection**									
Accession number	EMDB-14636	EMDB-14638	EMDB-14639	EMDB-14640	EMDB-14641	EMDB-14642	EMDB-14643	EMDB-14644	EMDB-14645
Magnification	105,000	105,000	105,000	105,000	105,000	105,000	105,000	105,000	105,000
Voltage / kV	300	300	300	300	300	300	300	300	300
Dose / $e^{-}Å^{-2}$	42	42	43	43	42	42	41	41	41
Pixel size / Å	0.837	0.837	0.837	0.837	0.837	0.837	0.837	0.837	0.837
Defocus range / µm	-1.1 to -2.1	-1.1 to -2.1	-1.1 to -2.1	-1.1 to -2.1	-1.1 to -2.1	-1.1 to -2.1	-1.1 to -2.1	-1.1 to -2.1	-1.1 to -2.1
Recorded movies	7,952	7,952	24,110	24,110	7,508	7,508	6,072	6,072	7,814
Final particle images	98,317	86,512	39592	87,939	71,865	89,336	57,308	89,870	644,226
Camera	Gatan K3	Gatan K3	Gatan K3	Gatan K3	Gatan K3	Gatan K3	Gatan K3	Gatan K3	Gatan K3
Energy filter	BioQuantum K3	BioQuantum K3	BioQuantum K3	BioQuantum K3	BioQuantum K3	BioQuantum K3	BioQuantum K3	BioQuantum K3	BioQuantum K3
Microscope	Titan Krios G3i	Titan Krios G3i	Titan Krios G3i	Titan Krios G3i	Titan Krios G3i	Titan Krios G3i	Titan Krios G3i	Titan Krios G3i	Titan Krios G3i
**Image processing**									
Initial model	De novo generated with RELION 3.1								
Resolution ($FSC_{0.143}$) / Å	3.17	3.05	3.17	3.35	3.65	3.13	3.17	2.94	2.77
Applied B-factor / $Å^2$	-73	-64	-57	-75	-102	-80	-59	-58	-70
**Model refinement**									
PDB accession	7ZD5	-	7ZDA	7ZDB	-	7ZDC	7ZDE	7ZDF	7ZDG
Validation									
$FSC^{map\text{-}to\text{-}model}_{(0.5)}$ / Å	3.1	-	3.2	3.3	-	3.1	3.0	2.9	2.7
MolProbity score	1.49	-	1.47	1.58	-	1.45	1.70	1.46	1.54
Composition									
Atoms	8,968	-	9,052	8,804	-	8,961	9,015	9,039	9,007
Protein residues	1,157	-	1,159	1,122	-	1,147	1,159	1,159	1,157
Ligands	-	-	MG: 2, ADP: 1, PO3: 1, ATP: 1	HEB: 1, MG: 2, ADP:1, PO3: 1, ATP: 1	-	HEB: 1, MG: 1, ADP: 1	MG: 1, ANP: 1	HEB: 1, MG: 1, ANP: 1	HEB: 1
Bonds (R.M.S.D.)									
Length (Å)	0.006	-	0.006	0.004	-	0.005	0.007	0.004	0.008
Angles (°)	0.709	-	0.706	0.740	-	0.727	0.770	0.651	0.811
B-factors (min/max/mean)									
Protein	13.98/105.03/46.35	-	3.35/79.70/25.61	14.56/96.07/47.96	-	8.65/79.13/33.20	15.71/126.14/51.59	13.99/81.56/38.33	12.90/93.23/48.08
Ligand	-	-	23.80/42.74/34.93	46.02/82.62/62.76	-	17.11/32.25/22.99	20.00/72.06/21.63	28.94/36.65/34.86	41.36/41.36/41.36
Clash score	9.26	-	8.71	11.21	-	7.57	10.71	7.61	9.39
Ramachandran plot (%)									
Favored	98.26	-	98.27	97.94	-	97.90	97.14	97.84	97.83
Allowed	1.74	-	1.73	2.06	-	2.10	2.86	2.16	2.17
Outliers	0	-	0	0	-	0	0	0	0
Rotamer outliers (%)	0.32	-	0.11	0	-	0	0	0	0

Supplementary Table 1 – Continued

	**Dataset 6** IF(heme/confined)	**Dataset 7** IF(apo/asym)	**Dataset 7** IF(heme/coordinated)	**Dataset 8** IF(apo/asym)	**Dataset8** IF(heme/coordinated)	**Dataset 9** IF(heme/confined)	**Dataset 10** IF(apo/as isolated)	**Dataset 10** IF(heme/confined)	**Dataset 11** IF(apo/as isolated)
**Data collection**									
Accession number	EMDB-14646	EMDB-14647	EMDB-14649	EMDB-14652	EMDB-14653	EMDB-14654	EMDB-14655	EMDB-14656	EMDB-14657
Magnification	105,000	105,000	105,000	105,000	105,000	105,000	105,000	105,000	105,000
Voltage / kV	300	300	300	300	300	300	300	300	300
Dose / $e^{-}Å^{-2}$	41	41	41	41	41	48	41	41	41
Pixel size / Å	0.837	0.837	0.837	0.837	0.837	0.831	0.837	0.837	0.837
Defocus range / µm	-1.1 to -2.1	-1.1 to -2.1	-1.1 to -2.1	-1.1 to -2.1	-1.1 to -2.1	-1.1 to -2.1	-1.1 to -2.1	-1.1 to -2.1	-1.1 to -2.1
Recorded movies	8,538	9,920	9,920	7,552	7,552	13,299	7,436	7,436	8,392
Final particle images	132,480	73,885	47,521	96,066	120,630	152,983	81,388	72,194	99,304
Camera	Gatan K3	Gatan K3	Gatan K3	Gatan K3	Gatan K3	Gatan K2 Summit	Gatan K3	Gatan K3	Gatan K3
Energy filter	BioQuantum K3	BioQuantum K3	BioQuantum K3	BioQuantum K3	BioQuantum K3	Quantum K2	BioQuantum K3	BioQuantum K3	BioQuantum K3
Microscope	Titan Krios G3i	Titan Krios G3i	Titan Krios G3i	Titan Krios G3i	Titan Krios G3i	Titan Krios G2	Titan Krios G3i	Titan Krios G3i	Titan Krios G3i
**Image processing**									
Initial model	De novo generated with RELION 3.1								
Resolution ($FSC_{0.143}$) / Å	3.09	3.49	3.49	3.01	3.35	3.42	3.26	3.35	3.44
Applied B-factor / $Å^2$	-72	-73	-88	-68	-89	-98	-88	-70	-95
**Model refinement**									
PDB accession	-	-	-	7ZDK	7ZDL	-	-	-	-
Validation									
$FSC^{map\text{-}to\text{-}model}_{(0.5)}$ / Å	-	-	-	3.0	3.3	-	-	-	-
MolProbity score	-	-	-	1.45	1.80	-	-	-	-
Composition									
Atoms	-	-	-	9,089	9,026	-	-	-	-
Protein residues	-	-	-	1,161	1,152	-	-	-	-
Ligands	-	-	-	MG: 2, ANP:2	HEB: 1, MG: 2, ANP: 2	-	-	-	-
Bonds (R.M.S.D.)									
Length (Å)	-	-	-	0.006	0.007	-	-	-	-
Angles (°)	-	-	-	0.775	0.844	-	-	-	-
B-factors (min/max/mean)									
Protein	-	-	-	8.26/63.36/24.44	19.33/101.69/48.81	-	-	-	-
Ligand	-	-	-	21.12/33.68/29.60	37.77/73.77/54.51	-	-	-	-
Clash score	-	-	-	8.29	11.54	-	-	-	-
Ramachandran plot (%)									
Favored	-	-	-	98.27	96.60	-	-	-	-
Allowed	-	-	-	1.73	3.40	-	-	-	-
Outliers	-	-	-	0	0	-	-	-	-
Rotamer outliers (%)	-	-	-	0	0	-	-	-	-

Supplementary Table 1 – Continued

	**Dataset 12** IF(heme/confined)	**Dataset 13** IF(apo/asym)	**Dataset 13** IF(heme/coordinated)	**Dataset 14** IF(heme/coordinated)	**Dataset 14** IF(heme/confined)	**Dataset 15** IF(heme/confined)	**Dataset 16** IF(apo/as isolated)	**Dataset 17** IF(apo/as isolated)	**Dataset 18** IF(apo/as isolated)
**Data collection**									
Accession number	EMDB-14659	EMDB-14660	EMDB-14662	EMDB-14663	EMDB-14665	EMDB-14689	EMDB-14667	EMDB-15264	EMDB-14668
Magnification	105,000	105,000	105,000	105,000	105,000	105,000	105,000	105,000	105,000
Voltage / kV	300	300	300	300	300	300	300	300	300
Dose / $e^{-}Å^{-2}$	41	41	41	41	41	41	41	41	41
Pixel size / Å	0.837	0.837	0.837	0.837	0.837	0.837	0.837	0.837	0.837
Defocus range / µm	-1.1 to -2.1	-1.1 to -2.1	-1.1 to -2.1	-1.1 to -2.1	-1.1 to -2.1	-1.1 to -2.1	-1.1 to -2.1	-1.1 to -2.1	-1.1 to -2.1
Recorded movies	8,936	10,092	10,092	9,576	9,576	8,009	5,372	9138	7,646
Final particle images	87,143	148,055	81,009	231,551	131,737	83,501	107,130	204256	88,567
Camera	Gatan K3	Gatan K3	Gatan K3	Gatan K3	Gatan K3	Gatan K3	Gatan K3	Gatan K3	Gatan K3
Energy filter	BioQuantum K3	BioQuantum K3	BioQuantum K3	BioQuantum K3	BioQuantum K3	BioQuantum K3	BioQuantum K3	BioQuantum K3	BioQuantum K3
Microscope	Titan Krios G3i	Titan Krios G3i	Titan Krios G3i	Titan Krios G3i	Titan Krios G3i	Titan Krios G3i	Titan Krios G3i	Titan Krios G3i	Titan Krios G3i
**Image processing**									
Initial model	De novo generated with RELION 3.1								
Resolution ($FSC_{0.143}$) / Å	3.44	3.26	3.17	3.05	2.87	3.05	3.05	3.26	3.26
Applied B-factor / $Å^2$	-91	-80	-81	-75	-60	-71	-65	-86	-86
**Model refinement**									
PDB accession	-	-	-	-	-	7ZEC	7ZDR	-	7ZDS
Validation									
$FSC^{map\text{-}to\text{-}model}_{(0.5)}$ / Å	-	-	-	-	-	3.0	3.0	-	3.2
MolProbity score	-	-	-	-	-	1.55	1.44	-	1.56
Composition									
Atoms	-	-	-	-	-	8,997	9,014	-	8,737
Protein residues	-	-	-	-	-	1,149	1,158	-	1,129
Ligands	-	-	-	-	-	HEB:1, MG: 1, ATP: 1	MG: 1, ANP: 1	-	-
Bonds (R.M.S.D.)									
Length (Å)	-	-	-	-	-	0.008	0.006	-	0.005
Angles (°)	-	-	-	-	-	0.800	0.641	-	0.724
B-factors (min/max/mean)									
Protein	-	-	-	-	-	11.45/83.58/39.04	16.22/100.95/42.68	-	7.26/96.17/38.30
Ligand	-	-	-	-	-	14.14/46.22/27.73	54.79/62.82/42.68	-	-
Clash score	-	-	-	-	-	8.15	8.13	-	10.98
Ramachandran plot (%)									
Favored	-	-	-	-	-	97.46	98.09	-	98.13
Allowed	-	-	-	-	-	2.54	1.91	-	1.87
Outliers	-	-	-	-	-	0	0	-	0
Rotamer outliers (%)	-	-	-	-	-	0	0	-	0

Supplementary Table 1 – Continued

	**Dataset 18** Occ(apo/return)	**Dataset 19** Occ(apo/return)	**Dataset 20** IF(apo/as isolated)	**Dataset 20** Occ(apo/return)	**Dataset 21** IF(heme/confined)	**Dataset 21** Occ(apo/return)	**Dataset 22** IF(heme/confined)	**Dataset 22** Occ(apo/return)	**Dataset 23** IF(heme/coordinated)
**Data collection**									
Accession number	EMDB-14669	EMDB-14670	EMDB-14671	EMDB-14672	EMDB-14673	EMDB-14674	EMDB-14675	EMDB-14676	EMDB-15265
Magnification	105,000	105,000	105,000	105,000	105,000	105,000	105,000	105,000	105,000
Voltage / kV	300	300	300	300	300	300	300	300	300
Dose / $e^{-}Å^{-2}$	41	41	41	41	41	41	41	41	41
Pixel size / Å	0.837	0.837	0.837	0.837	0.837	0.837	0.837	0.837	0.837
Defocus range / µm	-1.1 to -2.1	-1.1 to -2.1	-1.1 to -2.1	-1.1 to -2.1	-1.1 to -2.1	-1.1 to -2.1	-1.1 to -2.1	-1.1 to -2.1	-1.1 to -2.1
Recorded movies	7,646	15,860	8,856	8,856	8,090	8,090	2,294	2,294	8,270
Final particle images	96,900	102,009	114,003	132,215	77,061	130,996	44,248	44,418	50,573
Camera	Gatan K3	Gatan K3	Gatan K3	Gatan K3	Gatan K3	Gatan K2 Summit	Gatan K3	Gatan K3	Gatan K3
Energy filter	BioQuantum K3	BioQuantum K3	BioQuantum K3	BioQuantum K3	BioQuantum K3	Quantum K2	BioQuantum K3	BioQuantum K3	BioQuantum K3
Microscope	Titan Krios G3i	Titan Krios G3i	Titan Krios G3i	Titan Krios G3i	Titan Krios G3i	Titan Krios G2	Titan Krios G3i	Titan Krios G3i	Titan Krios G3i
**Image processing**									
Initial model	De novo generated with RELION 3.1								
Resolution ($FSC_{0.143}$) / Å	2.71	2.98	3.05	3.89	3.77	3.17	3.35	3.35	3.65
Applied B-factor / $Å^2$	-48	-46	-74	-114	-128	-70	-55	-61	-92
**Model refinement**									
PDB accession	7ZDT	7ZDU	7ZDV	-	-	-	7ZDW	-	-
Validation				-					-
$FSC^{map\text{-}to\text{-}model}_{(0.5)}$ / Å	2.7	3.0	3.0		-	-	3.3	-	
MolProbity score	1.35	1.45	1.50	-	-	-	1.72	-	-
Composition				-					-
Atoms	9,004	8,975	8,878	-	-	-	9,039	-	-
Protein residues	1,155	1,148	1,141		-	-	1,159	-	
Ligands	MG: 1, ATP: 1	MG: 2, ATP: 2	MG: 1, ANP: 1	-	-	-	HEB: 1, MG: 1, ANP: 1	-	-
Bonds (R.M.S.D.)				-					-
Length (Å)	0.005	0.004	0.005		-	-	0.007	-	
Angles (°)	0.701	0.626	0.659	-	-	-	0.818	-	-
B-factors (min/max/mean)				-					-
Protein	11.59/83.41/34.04	22.88/110.11/51.52	9.90/100.91/44.51	-	-	-	14.73/100.56/48.49	-	-
Ligand	24.76/27.64/27.55	42.80/48.91/45.86	66.48/69.69/69.59		-	-	24.16/61.09/39.79	-	
Clash score	6.21	8.23	8.86	-	-	-	9.91	-	-
Ramachandran plot (%)				-					-
Favored	98.70	98.25	97.88	-	-	-	96.80	-	-
Allowed	1.30	1.75	2.12	-	-	-	3.20	-	-
Outliers	0	0	0	-	-	-	0	-	-
Rotamer outliers (%)	0	0	0.11	-	-	-	0	-	-

Supplementary Table 1 – Continued

	**Dataset 23**<br>Occ(apo/return)
**Data collection**	
Accession number	EMDB-14684
Magnification	105,000
Voltage / kV	300
Dose / e^{-}Å^{-2}	41
Pixel size / Å	0.837
Defocus range / µm	-1.1 to -2.1
Recorded movies	8,270
Final particle images	74,444
Camera	Gatan K3
Energy filter	BioQuantum K3
Microscope	Titan Krios G3i
**Image processing**	
Initial model	De novo generated with RELION 3.1
Resolution ($FSC_{0.143}$) / Å	2.94
Applied B-factor / Å^2	-48
**Model refinement**	
PDB accession	7ZE5
Validation	
$FSC^{map\text{-}to\text{-}model}_{(0.5)}$ / Å	2.9
MolProbity score	1.42
Composition	
Atoms	9,077
Protein residues	1,159
Ligands	MG: 2, ATP:1, ANP: 1
Bonds (R.M.S.D.)	
Length (Å)	0.004
Angles (°)	0.589
B-factors (min/max/mean)	
Protein	26.14/94.30/48.12
Ligand	37.64/42.78/40.93
Clash score	7.65
Ramachandran plot (%)	
Favored	98.27
Allowed	1.73
Outliers	0
Rotamer outliers (%)	0

Supplementary Table 2 – Nucleotide sequences of CydDC variants used in this study.

Variant	Nucleotide sequence
CydDC (wild-type)	atgaattcgaataaatctcgtcaaaaagagttaacccgctggttaaaacagcaaagcgtcatctcccaacgttggctgaatatttctcgtctgctgggctttgtgag<br>cggcatattgatcattgcccaggcctggttcatggcgcgtattctgcaacatatgattatggagaatattccccgtgaagccctgctgcttccctttacgttactggtt<br>ctgacctttgtactgcgcgcatgggtggtctggttacgcgaacgggtgggttatcacgccgggcagcatatccgctttgccatccgccgtcaggttctcgaccgtct<br>gcaacaagcagggccagcgtggattcagggtaaacctgcggggagctgggcgacgctggtactcgagcaaattgacgatatgcatgattactatgcacgctatc<br>tgccgcaaatggcgctggcagtgtcggtgccgttgctgattgtggtggcaatcttcccctctaactgggctgcggcgctcattctgctgggcactgcaccgttaattc<br>cgttgtttatggcgctggttggaatgggggctgccgatgctaaccgacgtaactttctcgctcttgctcgcttaagtgggcatttcctcgatcgcctgcgcggcatgg<br>aaacattgcgtatttttggtcgtggtgaagctgaaattgaaagtattcgttctgcttcggaagatttccgccaacggacaatggaagtgctacggctggcgtttttat<br>cctccggcattctcgaatttttacctcgctgtcaattgctctggtggcggtctactttggtttttcctatctcggcgagctggattttggtcactacgataccggtgtga<br>cgctggctgcgggttttctggccctgatccttgcgccagagtttttccagccattacgcgatctcggtacgttttatcatgctaaagcccaggctgttggcgcagctg<br>acagtctgaaaacgtttatggaaaccccgctcgcccatccgcaacgtggtgaggcggaattagcatcgaccgatccggtgaccattgaggccgaggagctgttta<br>tcacgtcgccggaaggtaaaacgctggccggaccgctgaactttactttgccagcaggccaacgtgcggtgttggttggtcgcagcggttcaggtaaaagctcac<br>tgctgaacgcgctttctggttttctctcatatcagggatcgctacgaatcaacgggatagaattacgcgatttatcaccagaatcatggcgtaaacatctctcctcgg<br>ttgggcaaaacccacaattaccggcagcaacattgcgggataacgtactactggcgcgacctgatgccagcgaacaagaattacaagcagcgctggataacgc<br>ctgggtcagcgagtttctaccgctcctcccacaaggcgttgatacgcctgttggcgaccaggctgcccgcctttccgtggggcaggcgcagcgcgtggcggtggcc<br>cgtgcgttactaaatccctgttcgctattactgttggatgaacccgctgccagccttgatgctcacagtgaacagcgcgtaatggaggcgctgaatgccgcctctct<br>gcgccagacaacgttaatggtcacccaccagttagaagatcttgctgactgggatgtcatttgggttatgcaggatggccggattattgagcaaggacgttacgcg<br>gaattaagtgtggctggtggcccattcgccacattactggcccatcgtcaggaggagatttaaatgcgcgctttgctaccctatctggcactgtataaacgtcataa<br>atggatgttaagtcttggtattgtgctggcaattgtgacgctgctcgccagtatcggtctgttgacactttccggctggttcctctcggcctcagcggttgcggggtt<br>gccggactgtacagcttcaactatatgctacccgctgcgggcgtgcgtggcgcagcaatcacccgtactgccgggcgctattttgaacgtctggtaagtcacgacg<br>cgactttccgcgtgttgcagcatctgcgcatttacaccttcagcaaattgctgcccctctcccctgccggactggcgcgctatcgtcagggcgaattgctcaatcgcg<br>tggtggcggatgttgatacgctcgatcatctttacctgcgcgttatctcgccgctggtgggcgcttttgtggtgattatggtggtgacaatcgggttaagtttccttga<br>tttcaccctcgcctttacgctgggcggcattatgttactgacgcttttcctgatgccaccgctgttttatcgtgcgggaaaaagcaccgggcaaaatctgactcatctt<br>cgcggacagtatcgccaacaactgacggcctggctgcaagggcaagctgagctgaccatttttggtgccagcgatcgttatcgcacgcaactagagaatacaga<br>aattcaatggctggaagcgcaacgccgtcaatctgaactgaccgcattgtcgcaagcgataatgctgctcattggcgcgttagcggtgatcctgatgctgtggatg<br>gcgtctggcggcgttggcggcaatgctcaacccggcgcgttaattgccctgtttgtcttctgcgcgttagccgcgtttgaagcactggcaccagtaacgggtgcatt<br>tcagcatctggggcaagtcattgcctctgccgtacgtatctctgacttaacggatcaaaaaccggaggtcacctttcctgatacccaaactcgtgttgccgatcgcg<br>tttcgctgacgttacgggatgttcagttcacttatccggagcaatctcaacaggcacttaaagggatttctcttcaggtaaacgccggggaacatatagcgattctc<br>gggcgaaccggatgcggcaaatcaacactgttacaacagctgacccgcgcatgggacccgcaacagggcgagattttgcttaacgatagccccatagccagcct<br>gaatgaagcggctctacgacagaccatcagcgttgttcctcagcgagtgcatctgtttagcgccacgctgcgtgataatcttttactcgcctcgcctggcagtagtg<br>atgaggctctgtcggagatcttgcgtcgcgttggcctggaaaagctgctcgaggatgcaggtctcaacagttggttaggtgaaggcggacgccagctctccggtg<br>gtgaactgcgccgtctggctatcgcccgtgcgctgttacatgatgcgccactggtgttgctggatgaacctaccgaaggcttagatgccacaaccgaaagccagat<br>ccttgaattgcttgcagaaatgatgcgtgagaaaacggtgttaatggtcacccatcgacttcgcggactctctcgtttccaacaaataatagtgatggacaacggg<br>caaattattgagcaaggtactcacgcagaactgcttgccagacaggggcgttattaccagttcaagcagggtttgtctgcaggcggtcgtggcagccaccatcac<br>catcaccattaa
$CydD^{E511Q}C$	atgaattcgaataaatctcgtcaaaaagagttaacccgctggttaaaacagcaaagcgtcatctcccaacgttggctgaatatttctcgtctgctgggctttgtgag<br>cggcatattgatcattgcccaggcctggttcatggcgcgtattctgcaacatatgattatggagaatattccccgtgaagccctgctgcttccctttacgttactggtt<br>ctgacctttgtactgcgcgcatgggtggtctggttacgcgaacgggtgggttatcacgccgggcagcatatccgctttgccatccgccgtcaggttctcgaccgtct<br>gcaacaagcagggccagcgtggattcagggtaaacctgcggggagctgggcgacgctggtactcgagcaaattgacgatatgcatgattactatgcacgctatc<br>tgccgcaaatggcgctggcagtgtcggtgccgttgctgattgtggtggcaatcttcccctctaactgggctgcggcgctcattctgctgggcactgcaccgttaattc<br>cgttgtttatggcgctggttggaatgggggctgccgatgctaaccgacgtaactttctcgctcttgctcgcttaagtgggcatttcctcgatcgcctgcgcggcatgg<br>aaacattgcgtatttttggtcgtggtgaagctgaaattgaaagtattcgttctgcttcggaagatttccgccaacggacaatggaagtgctacggctggcgtttttat<br>cctccggcattctcgaatttttacctcgctgtcaattgctctggtggcggtctactttggtttttcctatctcggcgagctggattttggtcactacgataccggtgtga<br>cgctggctgcgggttttctggccctgatccttgcgccagagtttttccagccattacgcgatctcggtacgttttatcatgctaaagcccaggctgttggcgcagctg<br>acagtctgaaaacgtttatggaaaccccgctcgcccatccgcaacgtggtgaggcggaattagcatcgaccgatccggtgaccattgaggccgaggagctgttta<br>tcacgtcgccggaaggtaaaacgctggccggaccgctgaactttactttgccagcaggccaacgtgcggtgttggttggtcgcagcggttcaggtaaaagctcac<br>tgctgaacgcgctttctggttttctctcatatcagggatcgctacgaatcaacgggatagaattacgcgatttatcaccagaatcatggcgtaaacatctctcctcgg<br>ttgggcaaaacccacaattaccggcagcaacattgcgggataacgtactactggcgcgacctgatgccagcgaacaagaattacaagcagcgctggataacgc<br>ctgggtcagcgagtttctaccgctcctcccacaaggcgttgatacgcctgttggcgaccaggctgcccgcctttccgtggggcaggcgcagcgcgtggcggtggcc<br>cgtgcgttactaaatccctgttcgctattactgttggatCAGcccgctgccagccttgatgctcacagtgaacagcgcgtaatggaggcgctgaatgccgcctctc<br>tgcgccagacaacgttaatggtcacccaccagttagaagatcttgctgactgggatgtcatttgggttatgcaggatggccggattattgagcaaggacgttacgc<br>ggaattaagtgtggctggtggcccattcgccacattactggcccatcgtcaggaggagatttaaatgcgcgctttgctaccctatctggcactgtataaacgtcata<br>aatggatgttaagtcttggtattgtgctggcaattgtgacgctgctcgccagtatcggtctgttgacactttccggctggttcctctcggcctcagcggttgcggggt<br>tgccggactgtacagcttcaactatatgctacccgctgcgggcgtgcgtggcgcagcaatcacccgtactgccgggcgctattttgaacgtctggtaagtcacgac<br>gcgactttccgcgtgttgcagcatctgcgcatttacaccttcagcaaattgctgcccctctcccctgccggactggcgcgctatcgtcagggcgaattgctcaatcgc<br>gtggtggcggatgttgatacgctcgatcatctttacctgcgcgttatctcgccgctggtgggcgcttttgtggtgattatggtggtgacaatcgggttaagtttccttg<br>atttcaccctcgcctttacgctgggcggcattatgttactgacgcttttcctgatgccaccgctgttttatcgtgcgggaaaaagcaccgggcaaaatctgactcatc<br>ttcgcggacagtatcgccaacaactgacggcctggctgcaagggcaagctgagctgaccatttttggtgccagcgatcgttatcgcacgcaactagagaatacag<br>aaattcaatggctggaagcgcaacgccgtcaatctgaactgaccgcattgtcgcaagcgataatgctgctcattggcgcgttagcggtgatcctgatgctgtggat<br>ggcgtctggcggcgttggcggcaatgctcaacccggcgcgttaattgccctgtttgtcttctgcgcgttagccgcgtttgaagcactggcaccagtaacgggtgcat<br>ttcagcatctggggcaagtcattgcctctgccgtacgtatctctgacttaacggatcaaaaaccggaggtcacctttcctgatacccaaactcgtgttgccgatcgc<br>gtttcgctgacgttacgggatgttcagttcacttatccggagcaatctcaacaggcacttaaagggatttctcttcaggtaaacgccggggaacatatagcgattct<br>cgggcgaaccggatgcggcaaatcaacactgttacaacagctgacccgcgcatgggacccgcaacagggcgagattttgcttaacgatagccccatagccagc<br>ctgaatgaagcggctctacgacagaccatcagcgttgttcctcagcgagtgcatctgtttagcgccacgctgcgtgataatcttttactcgcctcgcctggcagtag<br>tgatgaggctctgtcggagatcttgcgtcgcgttggcctggaaaagctgctcgaggatgcaggtctcaacagttggttaggtgaaggcggacgccagctctccggt<br>ggtgaactgcgccgtctggctatcgcccgtgcgctgttacatgatgcgccactggtgttgctggatgaacctaccgaaggcttagatgccacaaccgaaagccag<br>atccttgaattgcttgcagaaatgatgcgtgagaaaacggtgttaatggtcacccatcgacttcgcggactctctcgtttccaacaaataatagtgatggacaacg<br>ggcaaattattgagcaaggtactcacgcagaactgcttgccagacaggggcgttattaccagttcaagcagggtttgtctgcaggcggtcgtggcagccaccatc<br>accatcaccattaa

CydDC^{E500Q}	atgaattcgaataaatctcgtcaaaaagagttaacccgctggttaaaacagcaaagcgtcatctcccaacgttggctgaatatttctcgtctgctgggctttgtgag<br>cggcatattgatcattgcccaggcctggttcatggcgcgtattctgcaacatatgattatggagaatattccccgtgaagccctgctgcttccctttacgttactggtt<br>ctgacctttgtactgcgcgcatgggtggtctggttacgcgaacgggtgggttatcacgccgggcagcatatccgctttgccatccgccgtcaggttctcgaccgtct<br>gcaacaagcagggccagcgtggattcagggtaaacctgcggggagctgggcgacgctggtactcgagcaaattgacgatatgcatgattactatgcacgctatc<br>tgccgcaaatggcgctggcagtgtcggtgccgttgctgattgtggtggcaatcttcccctctaactgggctgcggcgctcattctgctgggcactgcaccgttaattc<br>cgttgtttatggcgctggttggaatgggggctgccgatgctaaccgacgtaactttctcgctcttgctcgcttaagtgggcatttcctcgatcgcctgcgcggcatgg<br>aaacattgcgtatttttggtcgtggtgaagctgaaattgaaagtattcgttctgcttcggaagatttccgccaacggacaatggaagtgctacggctggcgtttttat<br>cctccggcattctcgaatttttacctcgctgtcaattgctctggtggcggtctactttggtttttcctatctcggcgagctggattttggtcactacgataccggtgtga<br>cgctggctgcgggttttctggccctgatccttgcgccagagtttttccagccattacgcgatctcggtacgttttatcatgctaaagcccaggctgttggcgcagctg<br>acagtctgaaaacgtttatggaaaccccgctcgcccatccgcaacgtggtgaggcggaattagcatcgaccgatccggtgaccattgaggccgaggagctgttta<br>tcacgtcgccggaaggtaaaacgctggccggaccgctgaactttactttgccagcaggccaacgtgcggtgttggttggtcgcagcggttcaggtaaaagctcac<br>tgctgaacgcgctttctggttttctctcatatcagggatcgctacgaatcaacgggatagaattacgcgatttatcaccagaatcatggcgtaaacatctctcctcgg<br>ttgggcaaaacccacaattaccggcagcaacattgcgggataacgtactactggcgcgacctgatgccagcgaacaagaattacaagcagcgctggataacgc<br>ctgggtcagcgagtttctaccgctcctcccacaaggcgttgatacgcctgttggcgaccaggctgcccgcctttccgtggggcaggcgcagcgcgtggcggtggcc<br>cgtgcgttactaaatccctgttcgctattactgttggatgaacccgctgccagccttgatgctcacagtgaacagcgcgtaatggaggcgctgaatgccgcctctct<br>gcgccagacaacgttaatggtcacccaccagttagaagatcttgctgactgggatgtcatttgggttatgcaggatggccggattattgagcaaggacgttacgcg<br>gaattaagtgtggctggtggcccattcgccacattactggcccatcgtcaggaggagatttaaatgcgcgctttgctaccctatctggcactgtataaacgtcataa<br>atggatgttaagtcttggtattgtgctggcaattgtgacgctgctcgccagtatcggtctgttgacactttccggctggttcctctcggcctcagcggttgcggggtt<br>gccggactgtacagcttcaactatatgctacccgctgcgggcgtgcgtggcgcagcaatcacccgtactgccgggcgctattttgaacgtctggtaagtcacgacg<br>cgactttccgcgtgttgcagcatctgcgcatttacaccttcagcaaattgctgcccctctcccctgccggactggcgcgctatcgtcagggcgaattgctcaatcgcg<br>tggtggcggatgttgatacgctcgatcatctttacctgcgcgttatctcgccgctggtgggcgcttttgtggtgattatggtggtgacaatcgggttaagtttccttga<br>tttcaccctcgcctttacgctgggcggcattatgttactgacgcttttcctgatgccaccgctgttttatcgtgcgggaaaaagcaccgggcaaaatctgactcatctt<br>cgcggacagtatcgccaacaactgacggcctggctgcaagggcaagctgagctgaccatttttggtgccagcgatcgttatcgcacgcaactagagaatacaga<br>aattcaatggctggaagcgcaacgccgtcaatctgaactgaccgcattgtcgcaagcgataatgctgctcattggcgcgttagcggtgatcctgatgctgtggatg<br>gcgtctggcggcgttggcggcaatgctcaacccggcgcgttaattgccctgtttgtcttctgcgcgttagccgcgtttgaagcactggcaccagtaacgggtgcatt<br>tcagcatctggggcaagtcattgcctctgccgtacgtatctctgacttaacggatcaaaaaccggaggtcacctttcctgatacccaaactcgtgttgccgatcgcg<br>tttcgctgacgttacgggatgttcagttcacttatccggagcaatctcaacaggcacttaaagggatttctcttcaggtaaacgccggggaacatatagcgattctc<br>gggcgaaccggatgcggcaaatcaacactgttacaacagctgacccgcgcatgggacccgcaacagggcgagattttgcttaacgatagccccatagccagcct<br>gaatgaagcggctctacgacagaccatcagcgttgttcctcagcgagtgcatctgtttagcgccacgctgcgtgataatcttttactcgcctcgcctggcagtagtg<br>atgaggctctgtcggagatcttgcgtcgcgttggcctggaaaagctgctcgaggatgcaggtctcaacagttggttaggtgaaggcggacgccagctctccggtg<br>gtgaactgcgccgtctggctatcgcccgtgcgctgttacatgatgcgccactggtgttgctggatCAGcctaccgaaggcttagatgccacaaccgaaagccaga<br>tccttgaattgcttgcagaaatgatgcgtgagaaaacggtgttaatggtcacccatcgacttcgcggactctctcgtttccaacaaataatagtgatggacaacgg<br>gcaaattattgagcaaggtactcacgcagaactgcttgccagacaggggcgttattaccagttcaagcagggtttgtctgcaggcggtcgtggcagccaccatca<br>ccatcaccattaa
CydDC^{H85A}	atgaattcgaataaatctcgtcaaaaagagttaacccgctggttaaaacagcaaagcgtcatctcccaacgttggctgaatatttctcgtctgctgggctttgtgag<br>cggcatattgatcattgcccaggcctggttcatggcgcgtattctgcaacatatgattatggagaatattccccgtgaagccctgctgcttccctttacgttactggtt<br>ctgacctttgtactgcgcgcatgggtggtctggttacgcgaacgggtgggttatcacgccgggcagcatatccgctttgccatccgccgtcaggttctcgaccgtct<br>gcaacaagcagggccagcgtggattcagggtaaacctgcggggagctgggcgacgctggtactcgagcaaattgacgatatgcatgattactatgcacgctatc<br>tgccgcaaatggcgctggcagtgtcggtgccgttgctgattgtggtggcaatcttcccctctaactgggctgcggcgctcattctgctgggcactgcaccgttaattc<br>cgttgtttatggcgctggttggaatgggggctgccgatgctaaccgacgtaactttctcgctcttgctcgcttaagtgggcatttcctcgatcgcctgcgcggcatgg<br>aaacattgcgtatttttggtcgtggtgaagctgaaattgaaagtattcgttctgcttcggaagatttccgccaacggacaatggaagtgctacggctggcgtttttat<br>cctccggcattctcgaatttttacctcgctgtcaattgctctggtggcggtctactttggtttttcctatctcggcgagctggattttggtcactacgataccggtgtga<br>cgctggctgcgggttttctggccctgatccttgcgccagagtttttccagccattacgcgatctcggtacgttttatcatgctaaagcccaggctgttggcgcagctg<br>acagtctgaaaacgtttatggaaaccccgctcgcccatccgcaacgtggtgaggcggaattagcatcgaccgatccggtgaccattgaggccgaggagctgttta<br>tcacgtcgccggaaggtaaaacgctggccggaccgctgaactttactttgccagcaggccaacgtgcggtgttggttggtcgcagcggttcaggtaaaagctcac<br>tgctgaacgcgctttctggttttctctcatatcagggatcgctacgaatcaacgggatagaattacgcgatttatcaccagaatcatggcgtaaacatctctcctcgg<br>ttgggcaaaacccacaattaccggcagcaacattgcgggataacgtactactggcgcgacctgatgccagcgaacaagaattacaagcagcgctggataacgc<br>ctgggtcagcgagtttctaccgctcctcccacaaggcgttgatacgcctgttggcgaccaggctgcccgcctttccgtggggcaggcgcagcgcgtggcggtggcc<br>cgtgcgttactaaatccctgttcgctattactgttggatgaacccgctgccagccttgatgctcacagtgaacagcgcgtaatggaggcgctgaatgccgcctctct<br>gcgccagacaacgttaatggtcacccaccagttagaagatcttgctgactgggatgtcatttgggttatgcaggatggccggattattgagcaaggacgttacgcg<br>gaattaagtgtggctggtggcccattcgccacattactggcccatcgtcaggaggagatttaaatgcgcgctttgctaccctatctggcactgtataaacgtcataa<br>atggatgttaagtcttggtattgtgctggcaattgtgacgctgctcgccagtatcggtctgttgacactttccggctggttcctctcggcctcagcggttgcggggtt<br>gccggactgtacagcttcaactatatgctacccgctgcgggcgtgcgtggcgcagcaatcacccgtactgccgggcgctattttgaacgtctggtaagtgcagacg<br>cgactttccgcgtgttgcagcatctgcgcatttacaccttcagcaaattgctgcccctctcccctgccggactggcgcgctatcgtcagggcgaattgctcaatcgcg<br>tggtggcggatgttgatacgctcgatcatctttacctgcgcgttatctcgccgctggtgggcgcttttgtggtgattatggtggtgacaatcgggttaagtttccttga<br>tttcaccctcgcctttacgctgggcggcattatgttactgacgcttttcctgatgccaccgctgttttatcgtgcgggaaaaagcaccgggcaaaatctgactcatctt<br>cgcggacagtatcgccaacaactgacggcctggctgcaagggcaagctgagctgaccatttttggtgccagcgatcgttatcgcacgcaactagagaatacaga<br>aattcaatggctggaagcgcaacgccgtcaatctgaactgaccgcattgtcgcaagcgataatgctgctcattggcgcgttagcggtgatcctgatgctgtggatg<br>gcgtctggcggcgttggcggcaatgctcaacccggcgcgttaattgccctgtttgtcttctgcgcgttagccgcgtttgaagcactggcaccagtaacgggtgcatt<br>tcagcatctggggcaagtcattgcctctgccgtacgtatctctgacttaacggatcaaaaaccggaggtcacctttcctgatacccaaactcgtgttgccgatcgcg<br>tttcgctgacgttacgggatgttcagttcacttatccggagcaatctcaacaggcacttaaagggatttctcttcaggtaaacgccggggaacatatagcgattctc<br>gggcgaaccggatgcggcaaatcaacactgttacaacagctgacccgcgcatgggacccgcaacagggcgagattttgcttaacgatagccccatagccagcct<br>gaatgaagcggctctacgacagaccatcagcgttgttcctcagcgagtgcatctgtttagcgccacgctgcgtgataatcttttactcgcctcgcctggcagtagtg<br>atgaggctctgtcggagatcttgcgtcgcgttggcctggaaaagctgctcgaggatgcaggtctcaacagttggttaggtgaaggcggacgccagctctccggtg<br>gtgaactgcgccgtctggctatcgcccgtgcgctgttacatgatgcgccactggtgttgctggatgaacctaccgaaggcttagatgccacaaccgaaagccagat<br>ccttgaattgcttgcagaaatgatgcgtgagaaaacggtgttaatggtcacccatcgacttcgcggactctctcgtttccaacaaataatagtgatggacaacggg<br>caaattattgagcaaggtactcacgcagaactgcttgccagacaggggcgttattaccagttcaagcagggtttgtctgcaggcggtcgtggcagccaccatcac<br>catcaccattaa

Supplementary Table 3 – Amino acid sequences of CydDC variants used in this study.

Variant	Amino acid sequence – CydD	Amino acid sequence – CydC
CydDC (wild-type)	MNSNKSRQKELTRWLKQQSVISQRWLNISRLLGFVSGILIIA<br>QAWFMARILQHMIMENIPREALLLPFTLLVLTFVLRAWVV<br>WLRERVGYHAGQHIRFAIRRQVLDRLQQAGPAWIQGKPA<br>GSWATLVLEQIDDMHDYYARYLPQMALAVSVPLLIVVAIFP<br>SNWAAALILLGTAPLIPLFMALVGMGAADANRRNFLALAR<br>LSGHFLDRLRGMETLRIFGRGEAEIESIRSASEDFRQRTMEV<br>LRLAFLSSGILEFFTSLSIALVAVYFGFSYLGELDFGHYDTGVT<br>LAAGFLALILAPEFFQPLRDLGTFYHAKAQAVGAADSLKTF<br>METPLAHPQRGEAELASTDPVTIEAEELFITSPEGKTLAGPL<br>NFTLPAGQRAVLVGRSGSGKSSLLNALSGFLSYQGSLRINGI<br>ELRDLSPESWRKHLSSVGQNPQLPAATLRDNVLLARPDASE<br>QELQAALDNAWVSEFLPLLPQGVDTPVGDQAARLSVGQA<br>QRVAVARALLNPCSLLLLDEPAASLDAHSEQRVMEALNAA<br>SLRQTTLMVTHQLEDLADWDVIWVMQDGRIIEQGRYAEL<br>SVAGGPFATLLAHRQEEI	MRALLPYLALYKRHKWMLSLGIVLAIVTLLASIGLLTLSGWF<br>LSASAVAGVAGLYSFNYMLPAAGVRGAAITRTAGRYFERLV<br>SHDATFRVLQHLRIYTFSKLLPLSPAGLARYRQGELLNRVVA<br>DVDTLDHLYLRVISPLVGAFVVIMVVTIGLSFLDFTLAFTLGG<br>IMLLTLFLMPPLFYRAGKSTGQNLTHLRGQYRQQLTAWLQ<br>GQAELTIFGASDRYRTQLENTEIQWLEAQRRQSELTALSQAI<br>MLLIGALAVILMLWMASGGVGGNAQPGALIALFVFCALAA<br>FEALAPVTGAFQHLGQVIASAVRISDLTDQKPEVTFPDTQT<br>RVADRVSLTLRDVQFTYPEQSQQALKGISLQVNAGEHIAIL<br>GRTGCGKSTLLQQLTRAWDPQQGEILLNDSPIASLNEAALR<br>QTISVVPQRVHLFSATLRDNLLLASPGSSDEALSEILRRVGLE<br>KLLEDAGLNSWLGEGGRQLSGGELRRLAIARALLHDAPLVL<br>LDEPTEGLDATTESQILELLAEMMREKTVLMVTHRLRGLSR<br>FQQIIVMDNGQIIEQGTHAELLARQGRYYQFKQGLSAGGR<br>GSHHHHHH
CydD^{E511Q}C	MNSNKSRQKELTRWLKQQSVISQRWLNISRLLGFVSGILIIA<br>QAWFMARILQHMIMENIPREALLLPFTLLVLTFVLRAWVV<br>WLRERVGYHAGQHIRFAIRRQVLDRLQQAGPAWIQGKPA<br>GSWATLVLEQIDDMHDYYARYLPQMALAVSVPLLIVVAIFP<br>SNWAAALILLGTAPLIPLFMALVGMGAADANRRNFLALAR<br>LSGHFLDRLRGMETLRIFGRGEAEIESIRSASEDFRQRTMEV<br>LRLAFLSSGILEFFTSLSIALVAVYFGFSYLGELDFGHYDTGVT<br>LAAGFLALILAPEFFQPLRDLGTFYHAKAQAVGAADSLKTF<br>METPLAHPQRGEAELASTDPVTIEAEELFITSPEGKTLAGPL<br>NFTLPAGQRAVLVGRSGSGKSSLLNALSGFLSYQGSLRINGI<br>ELRDLSPESWRKHLSSVGQNPQLPAATLRDNVLLARPDASE<br>QELQAALDNAWVSEFLPLLPQGVDTPVGDQAARLSVGQA<br>QRVAVARALLNPCSLLLLDQPAASLDAHSEQRVMEALNAA<br>SLRQTTLMVTHQLEDLADWDVIWVMQDGRIIEQGRYAEL<br>SVAGGPFATLLAHRQEEI	MRALLPYLALYKRHKWMLSLGIVLAIVTLLASIGLLTLSGWF<br>LSASAVAGVAGLYSFNYMLPAAGVRGAAITRTAGRYFERLV<br>SHDATFRVLQHLRIYTFSKLLPLSPAGLARYRQGELLNRVVA<br>DVDTLDHLYLRVISPLVGAFVVIMVVTIGLSFLDFTLAFTLGG<br>IMLLTLFLMPPLFYRAGKSTGQNLTHLRGQYRQQLTAWLQ<br>GQAELTIFGASDRYRTQLENTEIQWLEAQRRQSELTALSQAI<br>MLLIGALAVILMLWMASGGVGGNAQPGALIALFVFCALAA<br>FEALAPVTGAFQHLGQVIASAVRISDLTDQKPEVTFPDTQT<br>RVADRVSLTLRDVQFTYPEQSQQALKGISLQVNAGEHIAIL<br>GRTGCGKSTLLQQLTRAWDPQQGEILLNDSPIASLNEAALR<br>QTISVVPQRVHLFSATLRDNLLLASPGSSDEALSEILRRVGLE<br>KLLEDAGLNSWLGEGGRQLSGGELRRLAIARALLHDAPLVL<br>LDEPTEGLDATTESQILELLAEMMREKTVLMVTHRLRGLSR<br>FQQIIVMDNGQIIEQGTHAELLARQGRYYQFKQGLSAGGR<br>GSHHHHHH
CydDC^{E500Q}	MNSNKSRQKELTRWLKQQSVISQRWLNISRLLGFVSGILIIA<br>QAWFMARILQHMIMENIPREALLLPFTLLVLTFVLRAWVV<br>WLRERVGYHAGQHIRFAIRRQVLDRLQQAGPAWIQGKPA<br>GSWATLVLEQIDDMHDYYARYLPQMALAVSVPLLIVVAIFP<br>SNWAAALILLGTAPLIPLFMALVGMGAADANRRNFLALAR<br>LSGHFLDRLRGMETLRIFGRGEAEIESIRSASEDFRQRTMEV<br>LRLAFLSSGILEFFTSLSIALVAVYFGFSYLGELDFGHYDTGVT<br>LAAGFLALILAPEFFQPLRDLGTFYHAKAQAVGAADSLKTF<br>METPLAHPQRGEAELASTDPVTIEAEELFITSPEGKTLAGPL<br>NFTLPAGQRAVLVGRSGSGKSSLLNALSGFLSYQGSLRINGI<br>ELRDLSPESWRKHLSSVGQNPQLPAATLRDNVLLARPDASE<br>QELQAALDNAWVSEFLPLLPQGVDTPVGDQAARLSVGQA<br>QRVAVARALLNPCSLLLLDEPAASLDAHSEQRVMEALNAA<br>SLRQTTLMVTHQLEDLADWDVIWVMQDGRIIEQGRYAEL<br>SVAGGPFATLLAHRQEEI	MRALLPYLALYKRHKWMLSLGIVLAIVTLLASIGLLTLSGWF<br>LSASAVAGVAGLYSFNYMLPAAGVRGAAITRTAGRYFERLV<br>SHDATFRVLQHLRIYTFSKLLPLSPAGLARYRQGELLNRVVA<br>DVDTLDHLYLRVISPLVGAFVVIMVVTIGLSFLDFTLAFTLGG<br>IMLLTLFLMPPLFYRAGKSTGQNLTHLRGQYRQQLTAWLQ<br>GQAELTIFGASDRYRTQLENTEIQWLEAQRRQSELTALSQAI<br>MLLIGALAVILMLWMASGGVGGNAQPGALIALFVFCALAA<br>FEALAPVTGAFQHLGQVIASAVRISDLTDQKPEVTFPDTQT<br>RVADRVSLTLRDVQFTYPEQSQQALKGISLQVNAGEHIAIL<br>GRTGCGKSTLLQQLTRAWDPQQGEILLNDSPIASLNEAALR<br>QTISVVPQRVHLFSATLRDNLLLASPGSSDEALSEILRRVGLE<br>KLLEDAGLNSWLGEGGRQLSGGELRRLAIARALLHDAPLVL<br>LDQPTEGLDATTESQILELLAEMMREKTVLMVTHRLRGLSR<br>FQQIIVMDNGQIIEQGTHAELLARQGRYYQFKQGLSAGGR<br>GSHHHHHH
CydDC^{H85A}	MNSNKSRQKELTRWLKQQSVISQRWLNISRLLGFVSGILIIA<br>QAWFMARILQHMIMENIPREALLLPFTLLVLTFVLRAWVV<br>WLRERVGYHAGQHIRFAIRRQVLDRLQQAGPAWIQGKPA<br>GSWATLVLEQIDDMHDYYARYLPQMALAVSVPLLIVVAIFP<br>SNWAAALILLGTAPLIPLFMALVGMGAADANRRNFLALAR<br>LSGHFLDRLRGMETLRIFGRGEAEIESIRSASEDFRQRTMEV<br>LRLAFLSSGILEFFTSLSIALVAVYFGFSYLGELDFGHYDTGVT<br>LAAGFLALILAPEFFQPLRDLGTFYHAKAQAVGAADSLKTF<br>METPLAHPQRGEAELASTDPVTIEAEELFITSPEGKTLAGPL<br>NFTLPAGQRAVLVGRSGSGKSSLLNALSGFLSYQGSLRINGI<br>ELRDLSPESWRKHLSSVGQNPQLPAATLRDNVLLARPDASE<br>QELQAALDNAWVSEFLPLLPQGVDTPVGDQAARLSVGQA<br>QRVAVARALLNPCSLLLLDEPAASLDAHSEQRVMEALNAA<br>SLRQTTLMVTHQLEDLADWDVIWVMQDGRIIEQGRYAEL<br>SVAGGPFATLLAHRQEEI	MRALLPYLALYKRHKWMLSLGIVLAIVTLLASIGLLTLSGWF<br>LSASAVAGVAGLYSFNYMLPAAGVRGAAITRTAGRYFERLV<br>SADATFRVLQHLRIYTFSKLLPLSPAGLARYRQGELLNRVVA<br>DVDTLDHLYLRVISPLVGAFVVIMVVTIGLSFLDFTLAFTLGG<br>IMLLTLFLMPPLFYRAGKSTGQNLTHLRGQYRQQLTAWLQ<br>GQAELTIFGASDRYRTQLENTEIQWLEAQRRQSELTALSQAI<br>MLLIGALAVILMLWMASGGVGGNAQPGALIALFVFCALAA<br>FEALAPVTGAFQHLGQVIASAVRISDLTDQKPEVTFPDTQT<br>RVADRVSLTLRDVQFTYPEQSQQALKGISLQVNAGEHIAIL<br>GRTGCGKSTLLQQLTRAWDPQQGEILLNDSPIASLNEAALR<br>QTISVVPQRVHLFSATLRDNLLLASPGSSDEALSEILRRVGLE<br>KLLEDAGLNSWLGEGGRQLSGGELRRLAIARALLHDAPLVL<br>LDEPTEGLDATTESQILELLAEMMREKTVLMVTHRLRGLSR<br>FQQIIVMDNGQIIEQGTHAELLARQGRYYQFKQGLSAGGR<br>GSHHHHHH

Supplementary Table 4 – Description of the different simulations performed in this study.

Simulation reference	System name (CydDC conformation and heme state)	Description	Simulation time
**SIM 1**	IF_{apo_M1}	Simulation of $\mathbf{IF}^{apo}_{as\ isolated}$ in the presence of heme in the membrane	240 ns
**SIM 2**	IF_{apo_M2}	Simulation of $\mathbf{IF}^{apo}_{as\ isolated}$ in the presence of heme in the membrane (near the arm helix)	300 ns
**SIM 2.1**	$IF_{apo\_M2\_+\ Lysine\ mutants}$	Simulation of mutated $\mathbf{IF}^{apo}_{as\ isolated}$ ($K3^{D}$, $K7^{D}$, and $K314^{D}$ to alanine) in the presence of heme in the membrane (near the arm helix)	300 ns
**SIM 3**	IF_{apo_M3}	Simulation of $\mathbf{IF}^{apo}_{as\ isolated}$ in the presence of heme in the membrane (near the arm helix)	260 ns
**SIM 4**	IF_{apo_M4}	Simulation of $\mathbf{IF}^{apo}_{as\ isolated}$ in the presence of heme in the membrane (near the lateral entry site)	140 ns
**SIM 5**	IF_{apo_M5}	Simulation of $\mathbf{IF}^{apo}_{as\ isolated}$in the presence of heme in the membrane (near the lateral entry site)	300 ns
**SIM 6**	IF_{apo_M6}	Simulation of $\mathbf{IF}^{apo}_{as\ isolated}$ in the presence of heme in the membrane (near the lateral entry site)	220 ns
**SIM 7**	$IF_{Bound\_\ no\ axial\ bonds}$	Simulation of $\mathbf{IF}^{heme}_{bound}$ with heme in the binding site but without the covalent bond with $H85^{C}$	450 ns
**SIM 8**	$IF_{Bound\_\ H85^{C}\_axial\ bond}$	Simulation of $\mathbf{IF}^{heme}_{bound}$ with heme in the binding site and covalently bound to $H85^{C}$	450 ns
**SIM 9**	$IF_{Coordinated\_\ no\ axial\ bonds}$	Simulation of $\mathbf{IF}^{heme}_{bound}$ with heme in the binding site but without the covalent bonds with $H85^{C}$ and $H312^{D}$	450 ns
**SIM 10**	$IF_{Coordinated\_\ H85^{C}/\ His312^{C}\ axial\ bonds}$	Simulation of $\mathbf{IF}^{heme}_{coordinated}$ with heme in the binding site and covalently bound to $H85^{C}$and $H312^{D}$	450 ns
**SIM 11**	$IF_{Confined\_no\ axial\ bonds}$	Simulation of $\mathbf{IF}^{heme}_{confined}$ with heme in the binding site but without the covalent bonds with $H85^{C}$ and $H312^{D}$	450 ns
**SIM 11.1**	$IF_{Confined\_no\ axial\ bonds\ +\ R136A^{C}\ mutant}$	Simulation of mutated $\mathbf{IF}^{heme}_{confined}$ ($R136^{C}$ to alanine) with heme in the binding site but without the covalent bonds with $H85^{C}$ and $H312^{D}$	300 ns
**SIM 12**	$IF_{Confined\_\_\ H85^{C}/\ His312^{C}\ axial\ bonds}$	Simulation of $\mathbf{IF}^{heme}_{confined}$ with heme in the binding site and covalently bound to $H85^{C}$and $H312^{D}$	450 ns
**SIM 12.1**	$IF_{Confined\_H85^{C}/\ His312^{C}\ axial\ bonds\ +\ Arginine\ mutants}$	Simulation of mutated $\mathbf{IF}^{heme}_{confined}$ ($R77^{C}$ and $R81^{C}$ to alanine) with heme in the binding site and covalently bound to $H85^{C}$and $H312^{D}$	300 ns
**SIM 13**	$Occ_{Confined\_no\ axial\ bonds}$	Simulation of $\mathbf{Occ}^{heme}_{confined}$ model obtained from the slow growth method but without the covalent bonds with $H85^{C}$ and $H312^{D}$	200 ns
**SIM 14**	$Occ_{Confined\_H85^{C}/\ His312^{C}\ axial\ bonds}$	Simulation of $\mathbf{Occ}^{heme}_{confined}$ model obtained from the slow growth method with heme in the binding site and covalently bound to $H85^{C}$and $H312^{D}$	300 ns
**SIM 15**	$OF_{Confined\_no\ axial\ bonds\_M1}$	Simulation of $\mathbf{OF}^{heme}_{confined}$ model without the covalent bonds with $H85^{C}$ and $H312^{D}$ obtained from the steered MD simulations of $Occ_{Confined\_H85^{C}/\ His312^{C}\ axial\ bonds}$	300 ns
**SIM 16**	$OF_{Confined\_no\ axial\ bonds\ \_M2}$	Simulation of $\mathbf{OF}^{heme}_{confined}$ model without the covalent bonds with $H85^{C}$ and $H312^{D}$ obtained from the steered MD simulations of $Occ_{Confined\_no\ axial\ bonds}$	300 ns
**SIM 17**	$OF_{Confined\_H85^{C}/\ His312^{C}\ axial\ bonds}$	Simulation of $\mathbf{OF}^{heme}_{confined}$ model obtained from the steered MD simulations with heme in the binding site and covalently bound to $H85^{C}$and $H312^{D}$	300 ns
**SIM 18**	Occ_{Return}	Simulation of $\mathbf{Occ}^{apo}_{return}$	450 ns
**SIM 19**	OF_{Apo}	Simulation of $\mathbf{OF}^{apo}$ model obtained from the steered MD simulations	2 × 300 ns
**SIM 20**	Heme in membranes	Simulation of heme near a lipid bilayer composed of 70 % POPE, 25% POPG, and 5% CL lipids	10 × 100 ns

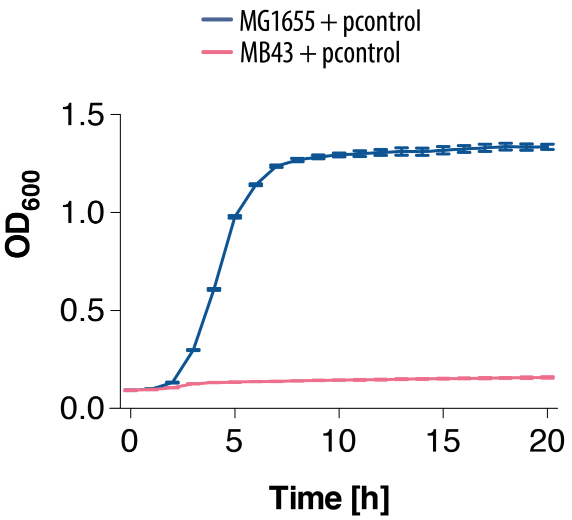

Supplementary Fig. 1 – Comparative growth of Escherichia coli strains MG1655 and MB43. MG1655 is the wild-type strain and MB43 is the mutant strain with impaired respiration. Both strains were transformed with the empty pET control vector (pcontrol). Data are presented as mean values +/- SD (n= 3 biological replicates).

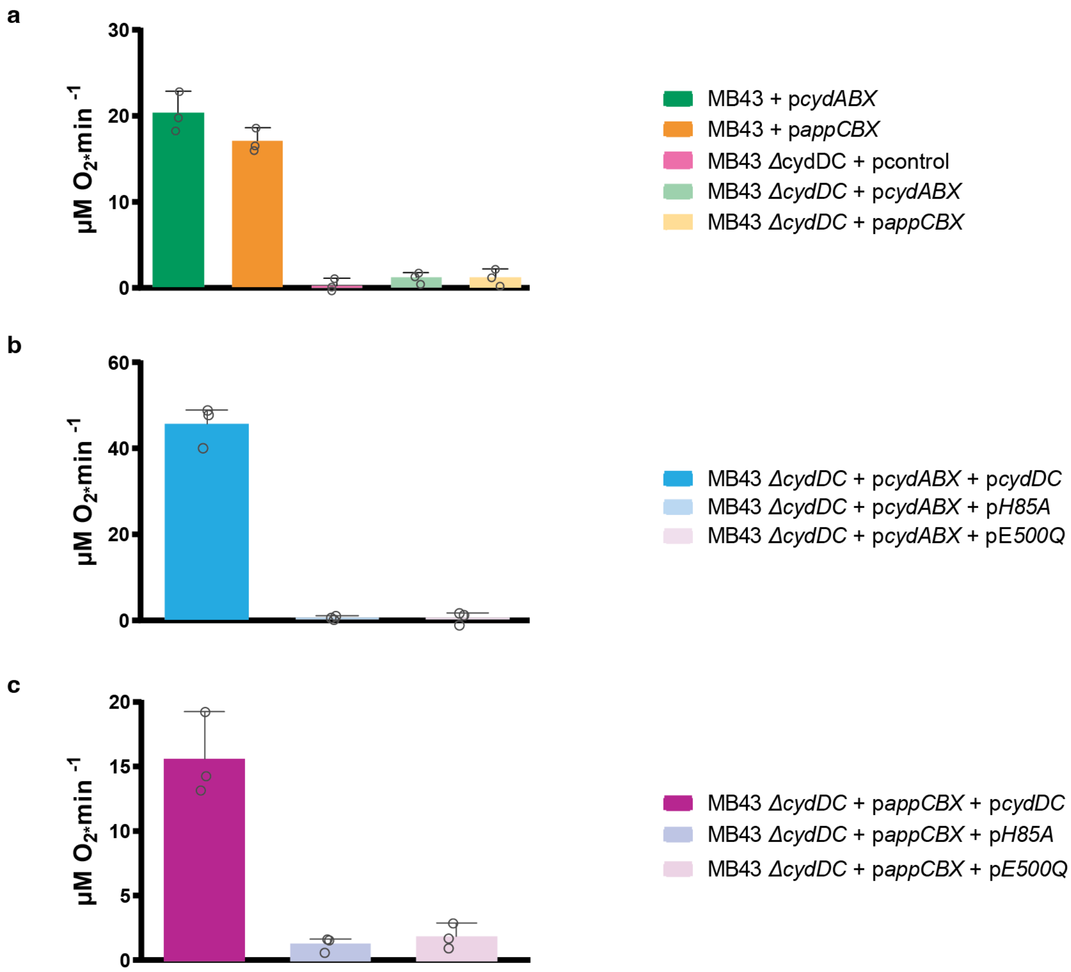

Supplementary Fig. 2 – Oxygen reductase activity of membranes isolated from bacterial strains. (a) Activity of MB43 and MB43Δ*cydDC* strains complemented with structural genes for cytochrome *bd*-I (*cydABX*) or cytochrome *bd*-II (*appCBX*), **(b)** activity of MB43Δ*cydDC* complemented with structural genes for cytochrome *bd*-I (*cydABX*) and for wild-type resp. mutant CydDC, **(c)** activity of MB43Δ*cydDC* complemented with structural genes for cytochrome *bd*-II (*appCBX*) and for wild-type resp. mutant CydDC. Oxygen concentrations were determined with a Clark-type electrode using membranes (5 µg for panels a and b, 15 µg for panel c). Samples were preincubated in buffer and subsequently the reaction was started by addition of the DTT/ubiquinol-1 substrate mixture. All presented data are given as mean values +/- SD (n= 3 biological replicates).

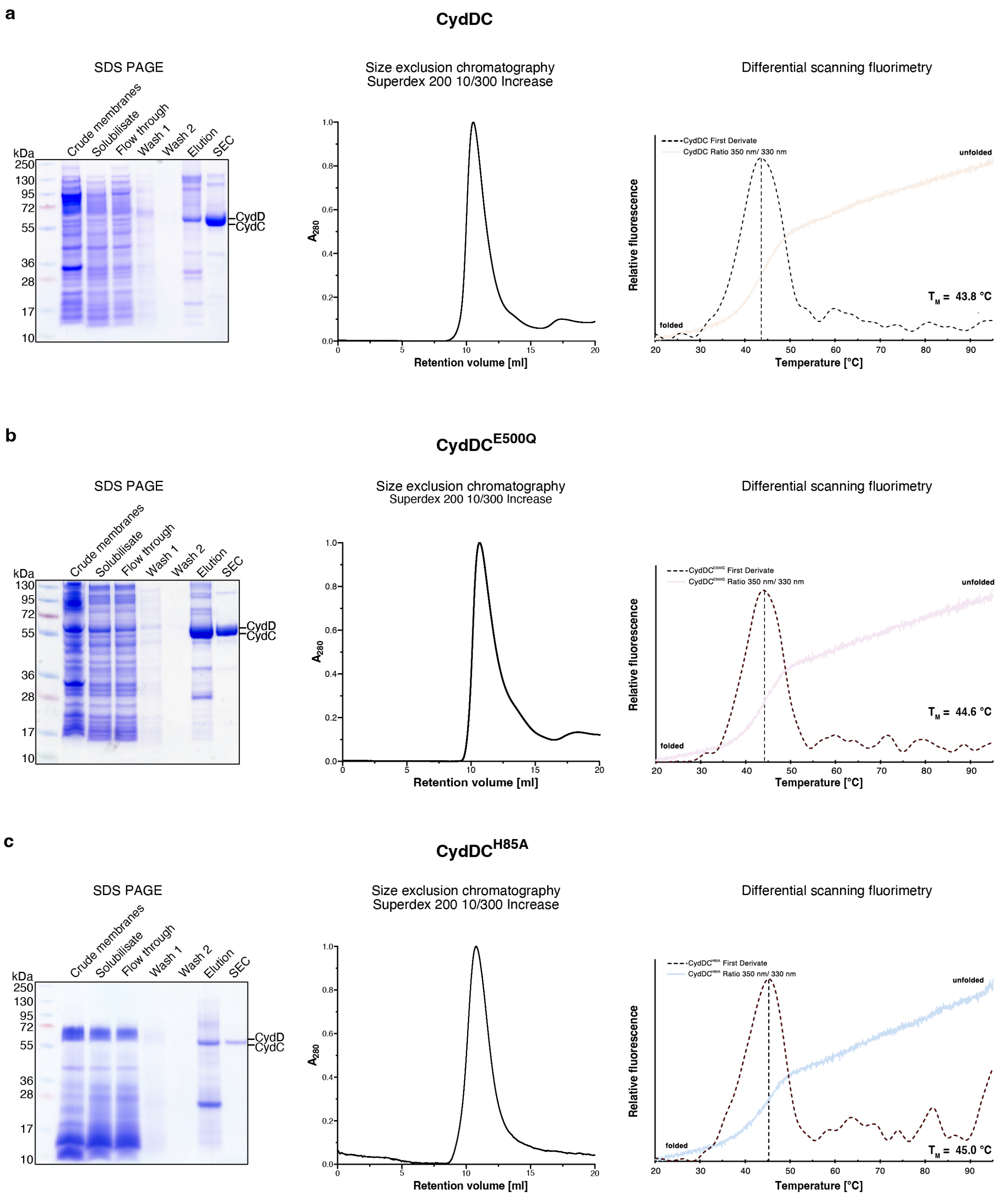

Supplementary Fig. 3 – Purification and characterization of CydDC variants. (a) Wild-type, **(b)** E500Q^{C}, and **(c)** H85A^{C} variants of CydDC were produced in *Escherichia coli* BL21 (DE3) cells and purified by immobilized metal ion affinity chromatography (Co-IMAC). Peak fractions were collected and analyzed by SDS-PAGE, size exclusion chromatography (SEC), nanoscale differential scanning fluorimetry (nanoDSF) and used for downstream activity assays and cryo-EM specimen preparation.

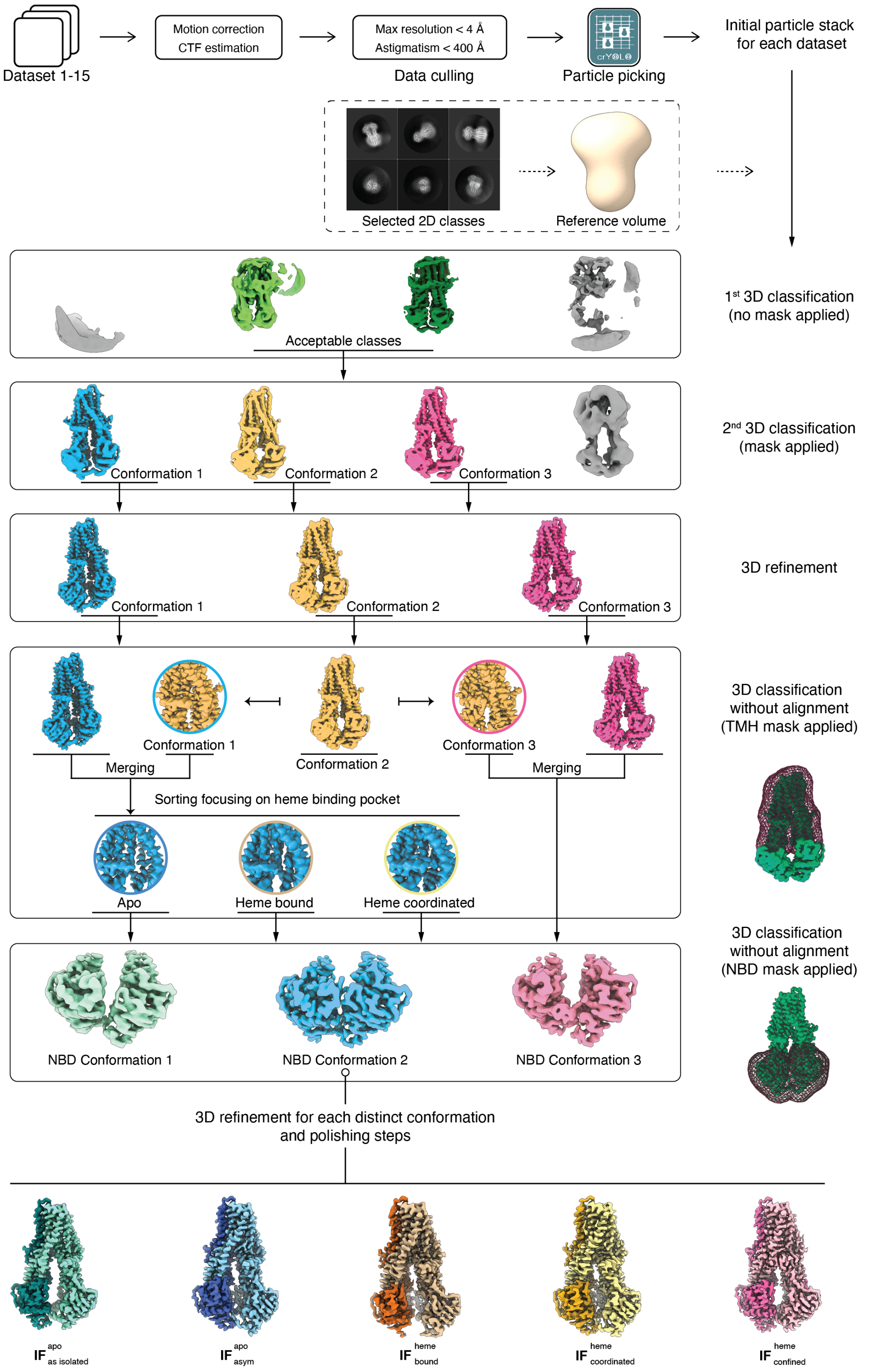

Supplementary Fig. 4 – Exemplary cryo-EM data processing workflow of wild-type CydDC datasets. Datasets 1 - 15 were processed according to the depicted workflow scheme. Statistical values of datasets 1 - 15 are summarized in supplementary table 1. Initial full-frame motion correction was performed with MotionCorr2 (RELION-3.1). CTF estimation was performed using Gctf (version 1.06). Particles were picked using crYOLO and subsequently extracted in the RELION-3.1 suite. Particles contributing to 2D classes with distinct features were selected for further processing. A subset of these particles was used for initial model generation. Three-dimensional classification, CTF refinement, Bayesian polishing, and consensus 3D refinement were performed in RELION-3.1.

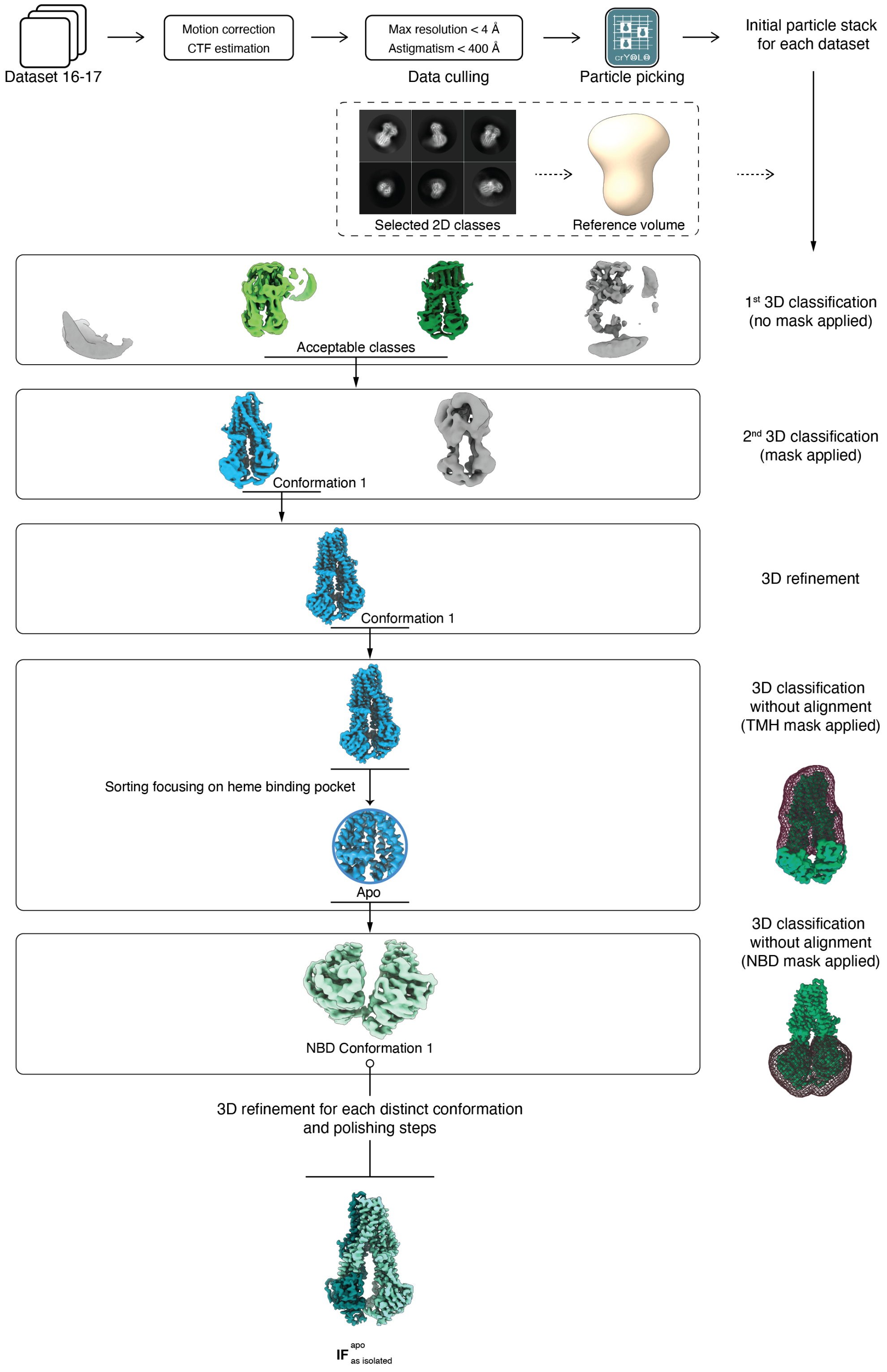

Supplementary Fig. 5 – Exemplary cryo-EM data processing workflow of H85A^{C} CydDC datasets. Datasets 16 and 17 were processed according to the depicted workflow scheme. Statistical values of datasets 16 and 17 are summarized in supplementary table 1. Initial full-frame motion correction was performed with MotionCorr2 (RELION-3.1). CTF estimation was performed using Gctf (version 1.06). Particles were picked using crYOLO and subsequently extracted in the RELION-3.1 suite. Particles contributing to 2D classes with distinct features were selected for further processing. A subset of these particles was used for initial model generation. Three-dimensional classification, CTF refinement, Bayesian polishing, and consensus 3D refinement were performed in RELION-3.1.

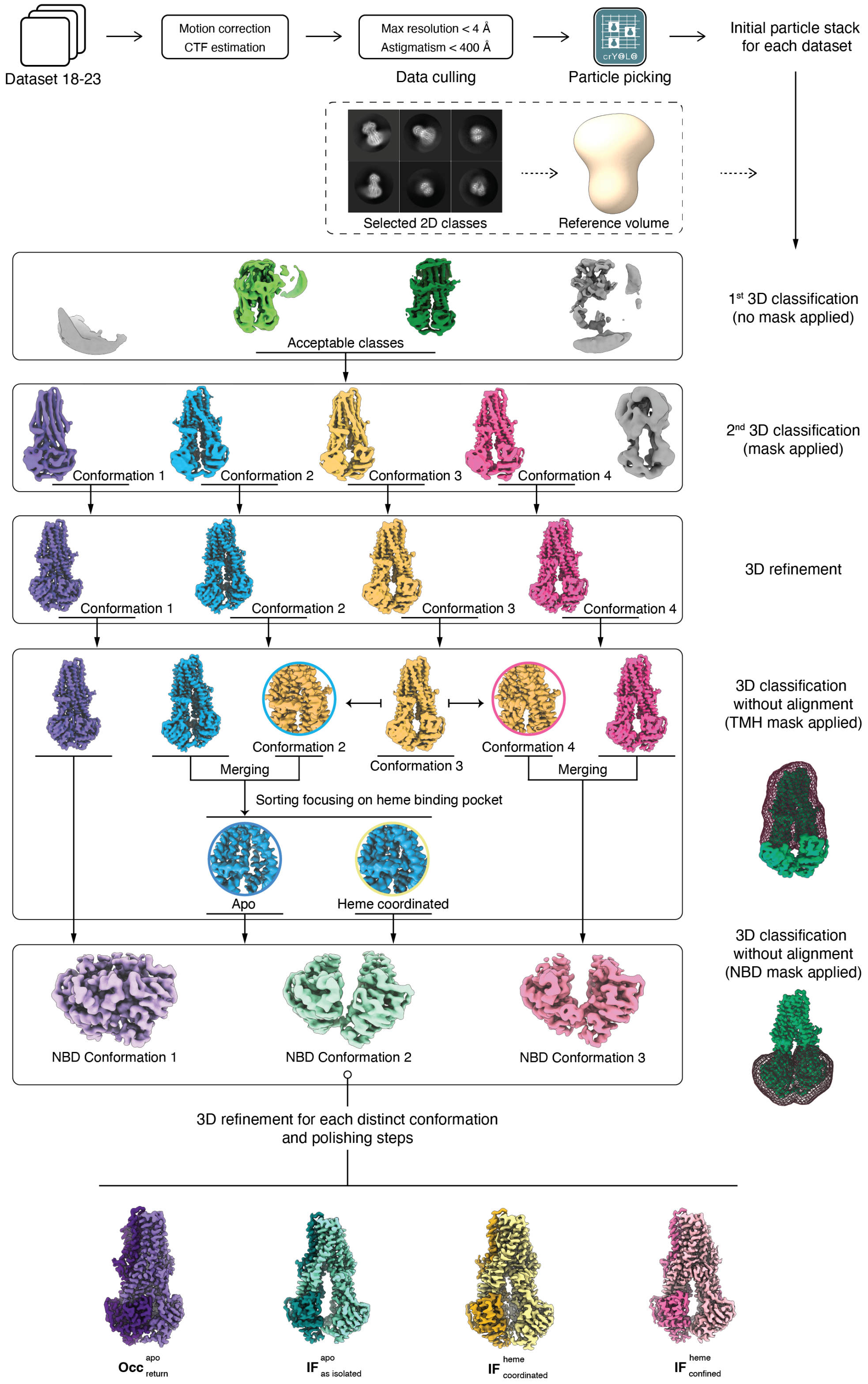

Supplementary Fig. 6 – Exemplary cryo-EM data processing workflow of E500Q^{C} CydDC datasets. Datasets 18 - 23 were processed according to the depicted workflow scheme. Statistical values of datasets 18 - 23 are summarized in supplementary table 1. Initial full-frame motion correction was performed with MotionCorr2 (RELION-3.1). CTF estimation was performed using Gctf (version 1.06). Particles were picked using crYOLO and subsequently extracted in the RELION-3.1 suite. Particles contributing to 2D classes with distinct features were selected for further processing. A subset of these particles was used for initial model generation. Three-dimensional classification, CTF refinement, Bayesian polishing, and consensus 3D refinement were performed in RELION-3.1.

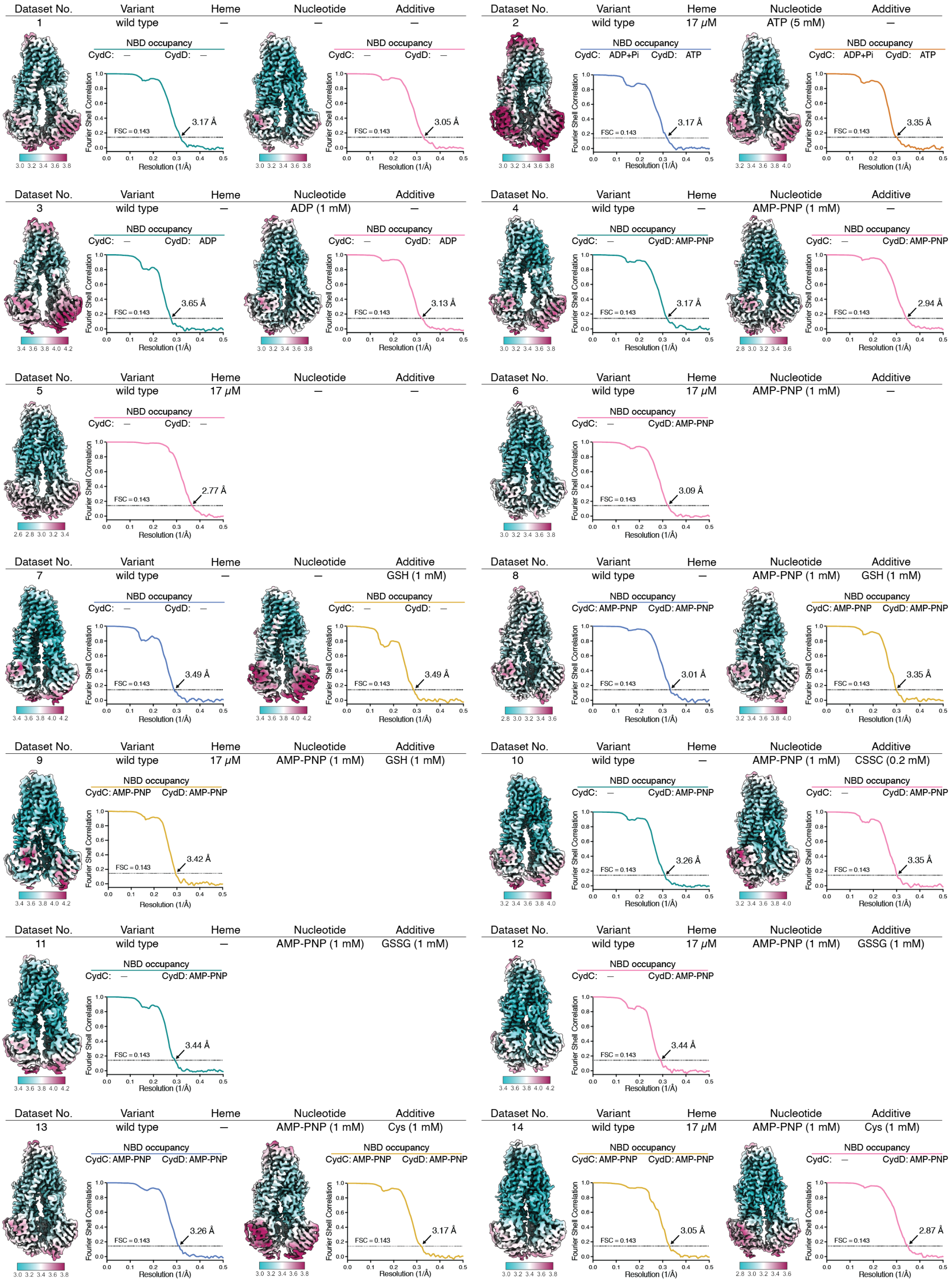
Dataset No. Variant Heme Nucleotide Additive
1 wild type — — —
NBD occupancy CydC: — CydD: —
3.17 Å
NBD occupancy CydC: — CydD: —
3.05 Å
2 wild type 17 μM ATP (5 mM) —
NBD occupancy CydC: ADP+Pi CydD: ATP
3.17 Å
NBD occupancy CydC: ADP+Pi CydD: ATP
3.35 Å
3 wild type — ADP (1 mM) —
NBD occupancy CydC: — CydD: ADP
3.65 Å
NBD occupancy CydC: — CydD: ADP
3.13 Å
4 wild type — AMP-PNP (1 mM) —
NBD occupancy CydC: — CydD: AMP-PNP
3.17 Å
NBD occupancy CydC: — CydD: AMP-PNP
2.94 Å
5 wild type 17 μM — —
NBD occupancy CydC: — CydD: —
2.77 Å
6 wild type 17 μM AMP-PNP (1 mM) —
NBD occupancy CydC: — CydD: AMP-PNP
3.09 Å
7 wild type — — GSH (1 mM)
NBD occupancy CydC: — CydD: —
3.49 Å
NBD occupancy CydC: — CydD: —
3.49 Å
8 wild type — AMP-PNP (1 mM) GSH (1 mM)
NBD occupancy CydC: AMP-PNP CydD: AMP-PNP
3.01 Å
NBD occupancy CydC: AMP-PNP CydD: AMP-PNP
3.35 Å
9 wild type 17 μM AMP-PNP (1 mM) GSH (1 mM)
NBD occupancy CydC: AMP-PNP CydD: AMP-PNP
3.42 Å
10 wild type — AMP-PNP (1 mM) CSSC (0.2 mM)
NBD occupancy CydC: — CydD: AMP-PNP
3.26 Å
NBD occupancy CydC: — CydD: AMP-PNP
3.35 Å
11 wild type — AMP-PNP (1 mM) GSSG (1 mM)
NBD occupancy CydC: — CydD: AMP-PNP
3.44 Å
12 wild type 17 μM AMP-PNP (1 mM) GSSG (1 mM)
NBD occupancy CydC: — CydD: AMP-PNP
3.44 Å
13 wild type — AMP-PNP (1 mM) Cys (1 mM)
NBD occupancy CydC: AMP-PNP CydD: AMP-PNP
3.26 Å
NBD occupancy CydC: AMP-PNP CydD: AMP-PNP
3.17 Å
14 wild type 17 μM AMP-PNP (1 mM) Cys (1 mM)
NBD occupancy CydC: AMP-PNP CydD: AMP-PNP
3.05 Å
NBD occupancy CydC: — CydD: AMP-PNP
2.87 Å
Fourier Shell Correlation
Resolution (1/Å)
FSC = 0.143

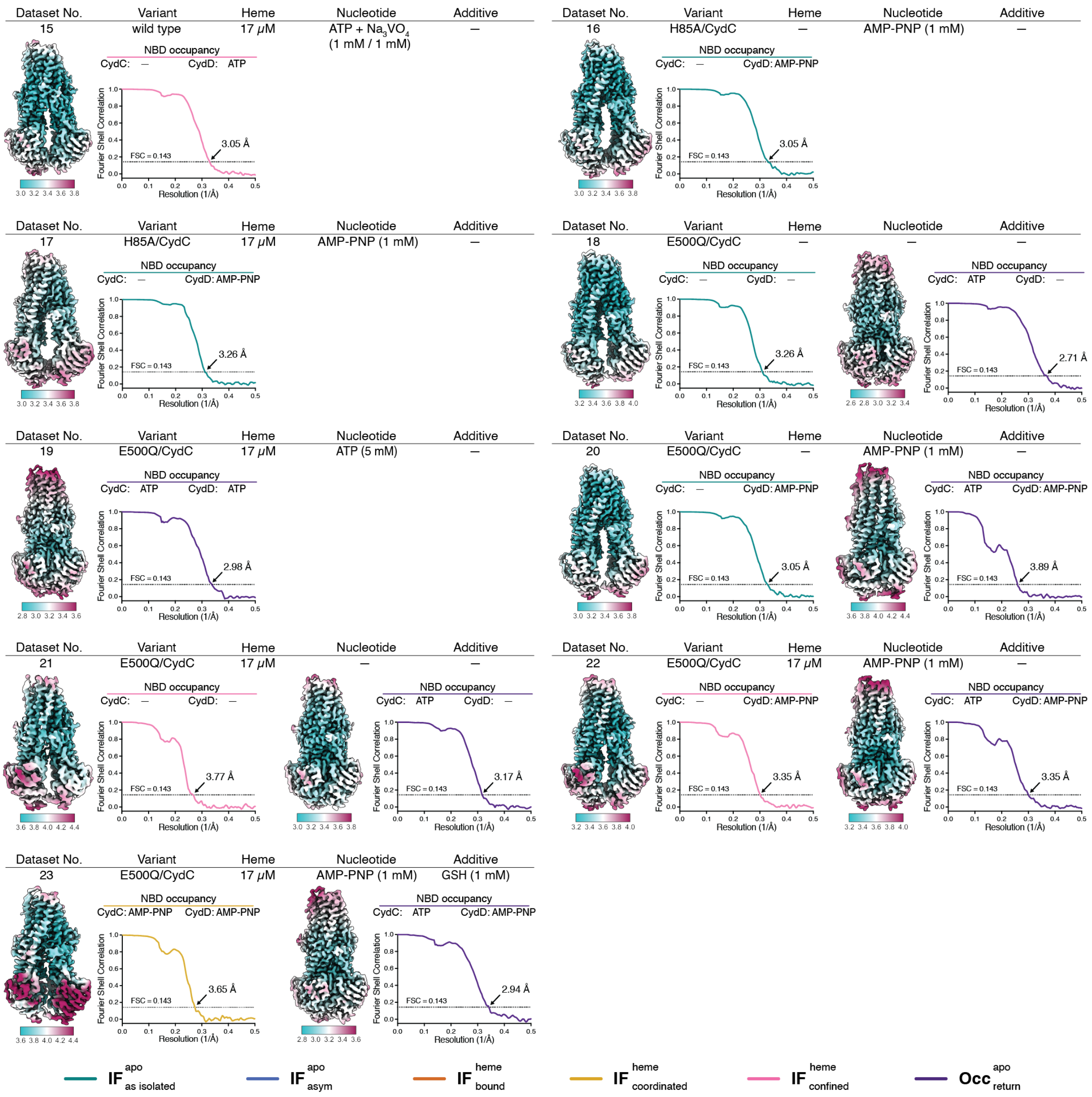

Supplementary Fig. 7 – Local resolution distributions, NBD occupancy, and Fourier shell correlations of CydDC structures. Color-coded density maps indicate local differences in resolution. The NBD occupancy of each individual map is indicated. Fourier shell correlation plots show average resolutions at $FSC_{0.143}$. Colors of FSC curves correspond to CydDC conformations according to the key at the bottom of the figure. Sample components and their respective concentrations are given for each dataset.

Model No.	Dataset No.	Variant	Heme	Nucleotide	Additive
1	1	wild type	—	—	—

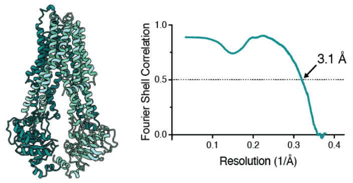

$\mathrm{IF}^{\mathrm{apo}}_{\mathrm{as\ isolated}}$

	Ligand assignment
Substrate binding site	–
NBS^{C}	–
NBS^{D}	–

Model No.	Dataset No.	Variant	Heme	Nucleotide	Additive
2	2	wild type	17 μM	ATP (5 mM)	—

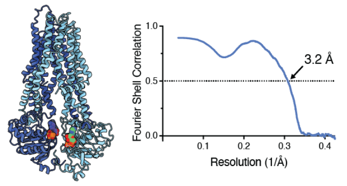

$\mathrm{IF}^{\mathrm{apo}}_{\mathrm{asym}}$

	Ligand assignment
Substrate binding site	–
NBS^{C}	ADP, Pi
NBS^{D}	ATP

Model No.	Dataset No.	Variant	Heme	Nucleotide	Additive
3	2	wild type	17 μM	ATP (5 mM)	—

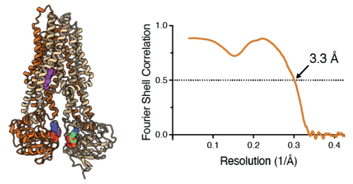

$\mathrm{IF}^{\mathrm{heme}}_{\mathrm{bound}}$

	Ligand assignment
Substrate binding site	heme
NBS^{C}	ADP, Pi
NBS^{D}	ATP

Model No.	Dataset No.	Variant	Heme	Nucleotide	Additive
4	3	wild type	—	ADP (1 mM)	—

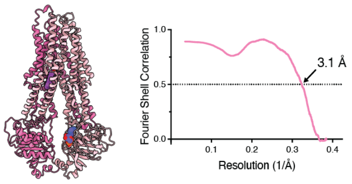

$\mathrm{IF}^{\mathrm{heme}}_{\mathrm{confined}}$

	Ligand assignment
Substrate binding site	heme
NBS^{C}	–
NBS^{D}	ADP

Model No.	Dataset No.	Variant	Heme	Nucleotide	Additive
5	4	wild type	—	AMP-PNP (1 mM)	—

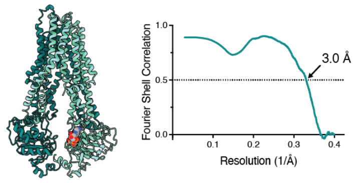

$\mathrm{IF}^{\mathrm{apo}}_{\mathrm{as\ isolated}}$

	Ligand assignment
Substrate binding site	–
NBS^{C}	–
NBS^{D}	AMP-PNP

Model No.	Dataset No.	Variant	Heme	Nucleotide	Additive
6	4	wild type	—	AMP-PNP (1 mM)	—

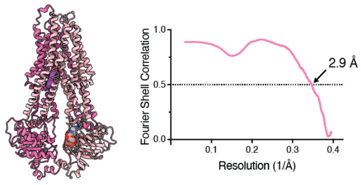

$\mathrm{IF}^{\mathrm{heme}}_{\mathrm{confined}}$

	Ligand assignment
Substrate binding site	heme
NBS^{C}	–
NBS^{D}	AMP-PNP

Model No.	Dataset No.	Variant	Heme	Nucleotide	Additive
7	5	wild type	17 μM	—	—

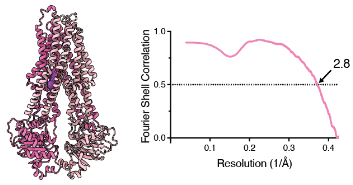

$\mathrm{IF}^{\mathrm{heme}}_{\mathrm{confined}}$

	Ligand assignment
Substrate binding site	heme
NBS^{C}	–
NBS^{D}	–

Model No.	Dataset No.	Variant	Heme	Nucleotide	Additive
8	8	wild type	—	AMP-PNP (1 mM)	GSH (1 mM)

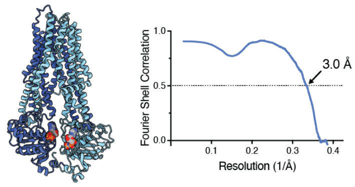

$\mathrm{IF}^{\mathrm{apo}}_{\mathrm{asym}}$

	Ligand assignment
Substrate binding site	–
NBS^{C}	AMP-PNP
NBS^{D}	AMP-PNP

Model No.	Dataset No.	Variant	Heme	Nucleotide	Additive
9	8	wild type	—	AMP-PNP (1 mM)	GSH (1 mM)

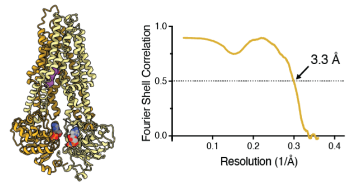

$\mathrm{IF}^{\mathrm{heme}}_{\mathrm{coordinated}}$

	Ligand assignment
Substrate binding site	heme
NBS^{C}	AMP-PNP
NBS^{D}	AMP-PNP

Model No.	Dataset No.	Variant	Heme	Nucleotide	Additive
10	15	wild type	—	ATP+Na_3VO_4 (1 mM/1 mM)	—

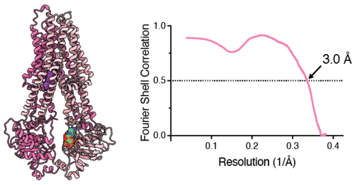

$\mathrm{IF}^{\mathrm{heme}}_{\mathrm{confined}}$

	Ligand assignment
Substrate binding site	heme
NBS^{C}	–
NBS^{D}	ATP

Model No.	Dataset No.	Variant	Heme	Nucleotide	Additive
11	16	H85A/CydC	—	AMP-PNP (1 mM)	—

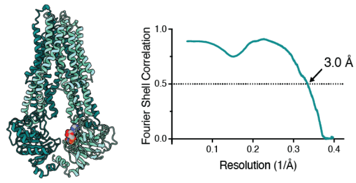

$\mathrm{IF}^{\mathrm{apo}}_{\mathrm{as\ isolated}}$

	Ligand assignment
Substrate binding site	–
NBS^{C}	–
NBS^{D}	AMP-PNP

Model No.	Dataset No.	Variant	Heme	Nucleotide	Additive
12	18	E500Q/CydC	—	—	—

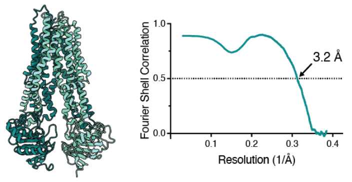

$\mathrm{IF}^{\mathrm{apo}}_{\mathrm{as\ isolated}}$

	Ligand assignment
Substrate binding site	–
NBS^{C}	–
NBS^{D}	–

Model No.	Dataset No.	Variant	Heme	Nucleotide	Additive
13	18	E500Q/CydC	—	—	—

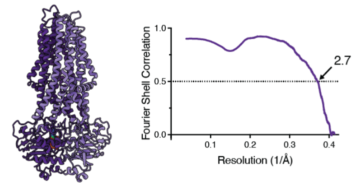

$\mathrm{Occ}^{\mathrm{apo}}_{\mathrm{return}}$

	Ligand assignment
Substrate binding site	–
NBS^{C}	ATP
NBS^{D}	–

Model No.	Dataset No.	Variant	Heme	Nucleotide	Additive
14	19	E500Q/CydC	17 μM	ATP (5 mM)	—

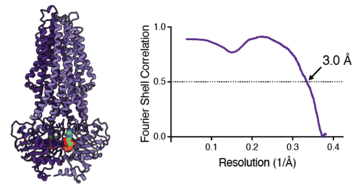

$\mathrm{Occ}^{\mathrm{apo}}_{\mathrm{return}}$

	Ligand assignment
Substrate binding site	–
NBS^{C}	ATP
NBS^{D}	ATP

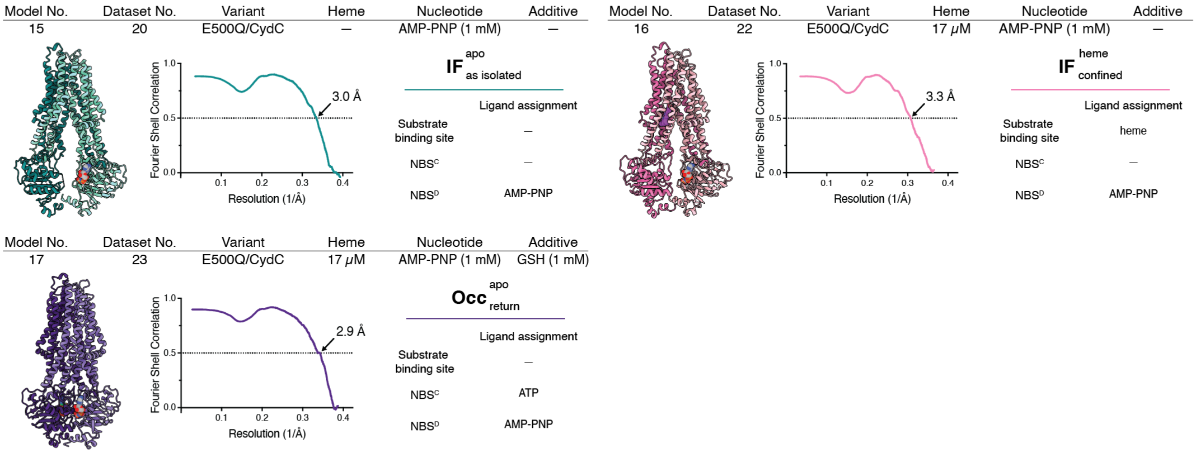

Supplementary Fig. 8 – Map-to-model correlations. Ribbon models corresponding to conformations identified in respective cryo-EM datasets are shown. Fourier shell plots show map-to-model correlations. Resolution estimates are based on $FSC_{0.5}$ level. Ligand assignments are listed for each model and dataset. Colors of models and FSC curves correspond to CydDC conformations: $\mathbf{IF}^{\mathrm{apo}}_{\mathrm{asym}}$, blue; $\mathbf{IF}^{\mathrm{apo}}_{\mathrm{as\ isolated}}$, green; $\mathbf{IF}^{\mathrm{heme}}_{\mathrm{bound}}$, orange; $\mathbf{IF}^{\mathrm{heme}}_{\mathrm{coordinated}}$, yellow; $\mathbf{IF}^{\mathrm{heme}}_{\mathrm{confined}}$, magenta; $\mathbf{Occ}^{\mathrm{apo}}_{\mathrm{return}}$, purple. Ligand colors: ATP, green; ADP, purple; AMP-PNP, grey; heme, violet.

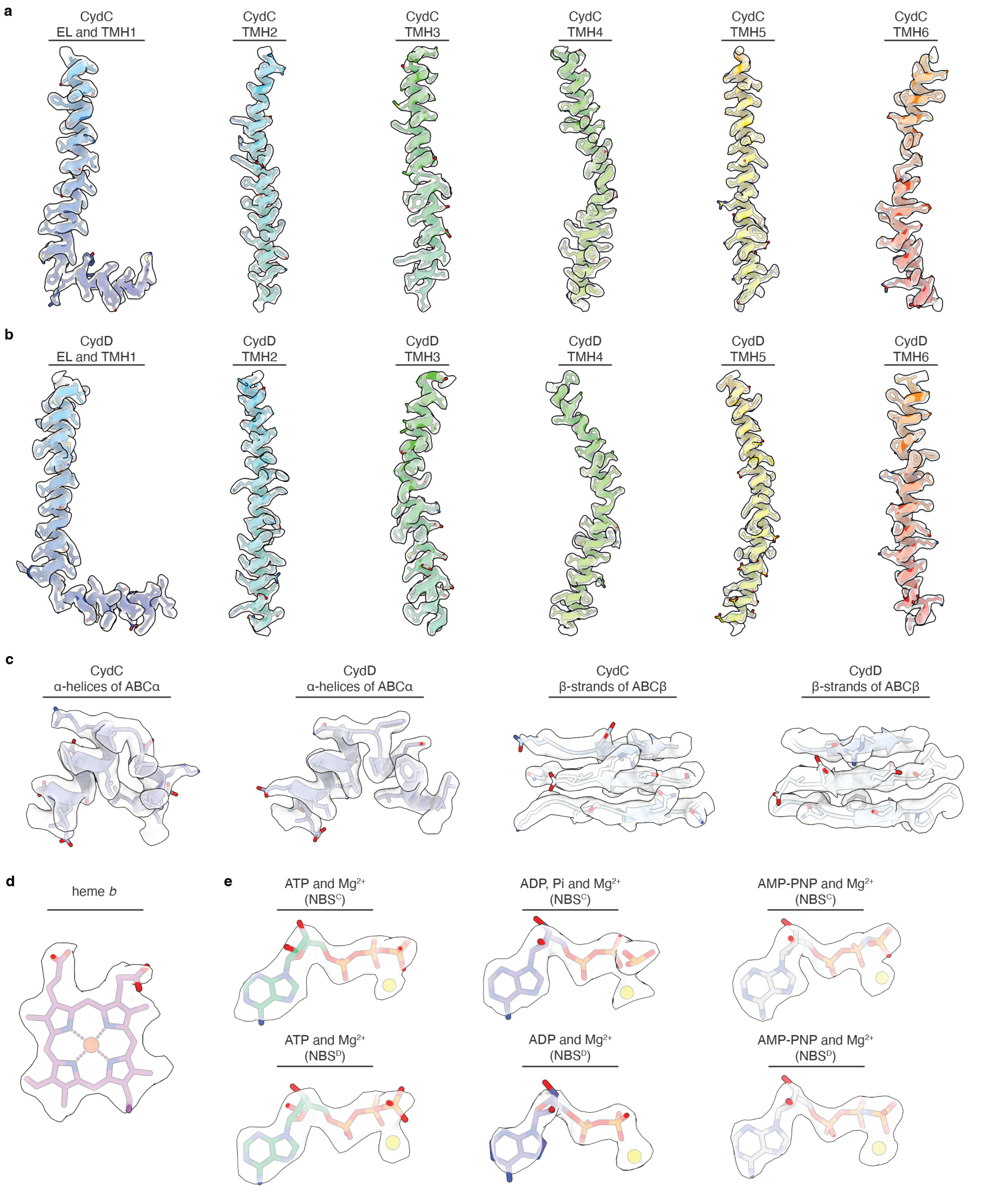

Supplementary Fig. 9 – Representative density features of CydDC. Visual inspection of density features of **(a-b)** transmembrane helices, **(c)** nucleotide-binding domain motifs, and **(d-e)** substrates/inhibitors (heme, ATP, ADP + Pi, and AMP-PNP). Symmetry related structural elements are presented in matching color codes. Presented densities were sharpened by b-factors of between -80 and -48.

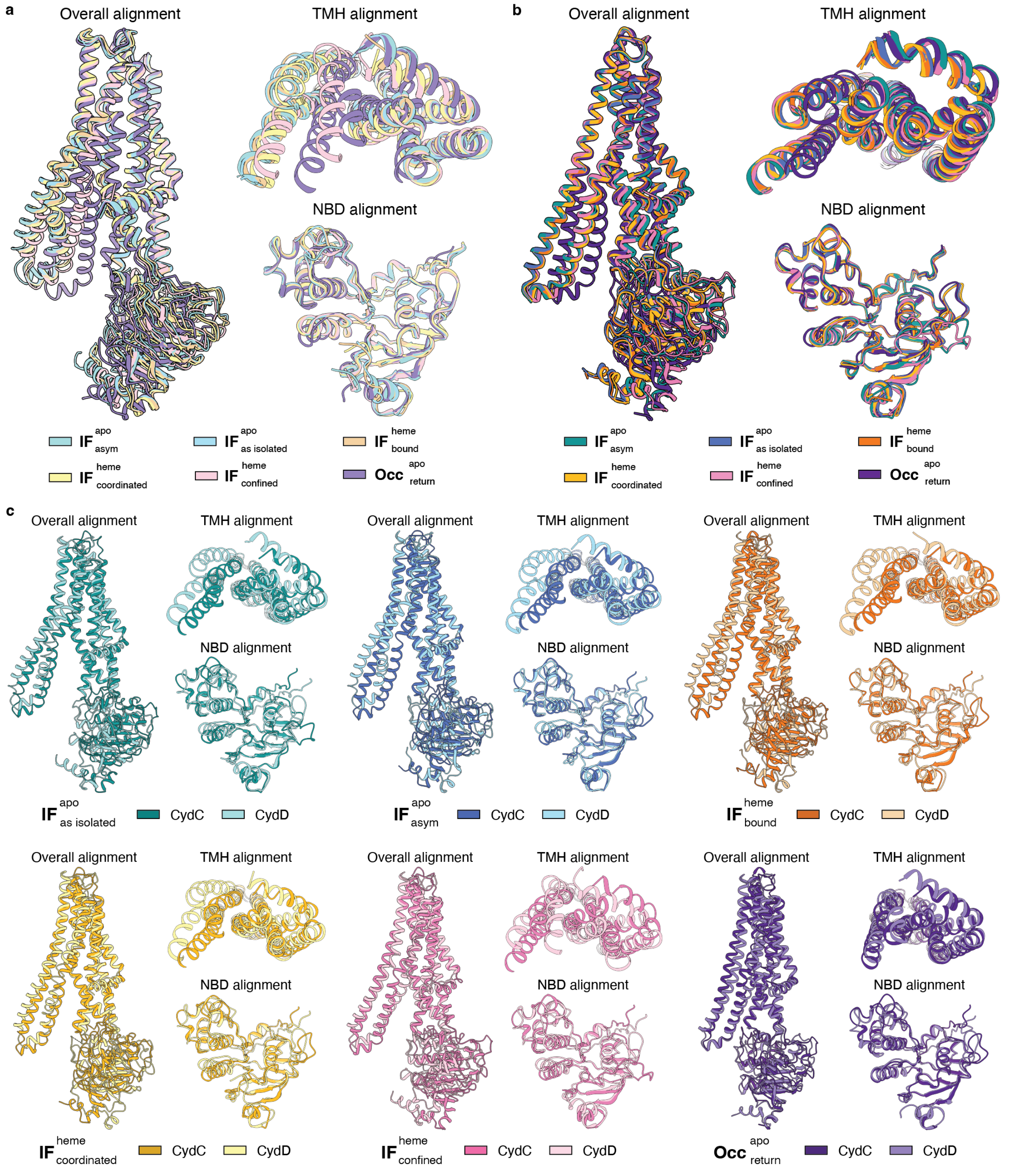

Supplementary Fig. 10 – Changing structural symmetry during the transport cycle states of CydDC. **(a)** Structural superimposition of CydD conformations obtained by cryo-EM. **(b)** Structural superimposition of CydC conformations obtained by cryo-EM. **(c)** Structural superimposition of subunits representing each conformational state. Structural alignments are shown between subunits, TMH domains, and NB domains, respectively.

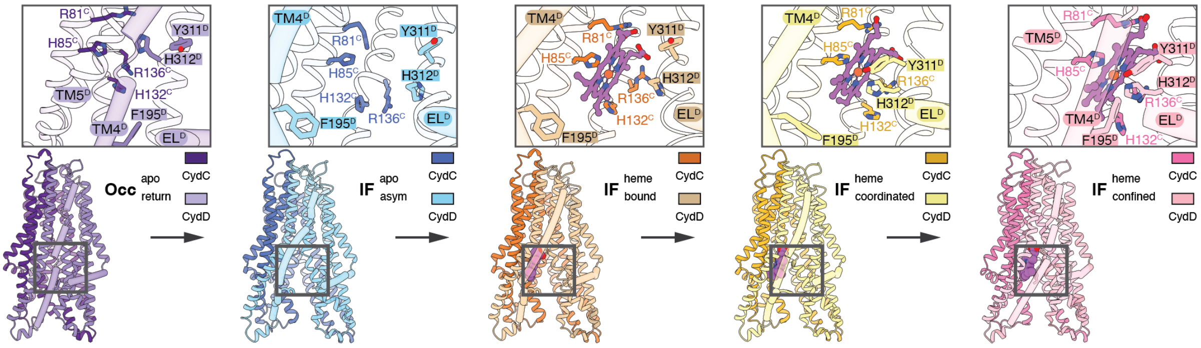

Supplementary Fig. 11 – Mechanism of heme binding and translocation. Overview of TMH conformations during heme binding, coordination, confinement and translocation. Closeup side views show TMs and residues that form the heme binding site and the lateral entry gate. Heme is shown as purple ball-and-stick model. EL1^{D} and TM4^{D} are shown as tubes.

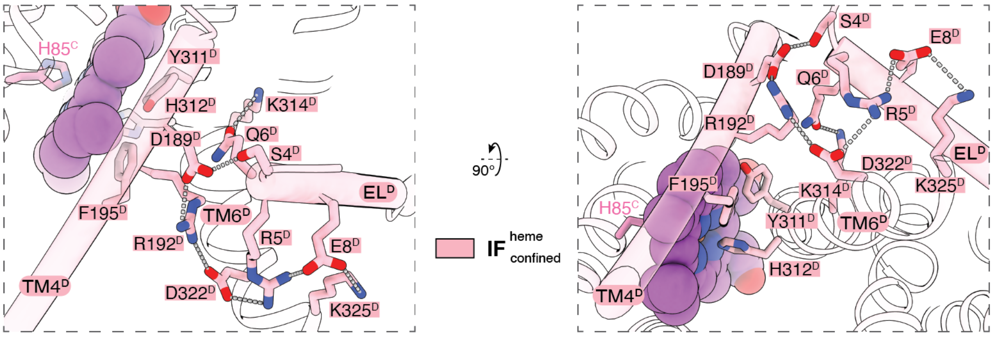

Supplementary Fig. 12 – Molecular details of the heme binding site and the membrane exposed entry gate. Closing of the lateral substrate entry site is facilitated by the formation of an electrostatic interaction network between residues of EL^D and TM4^D. Electrostatic interactions are indicated by dashed lines.

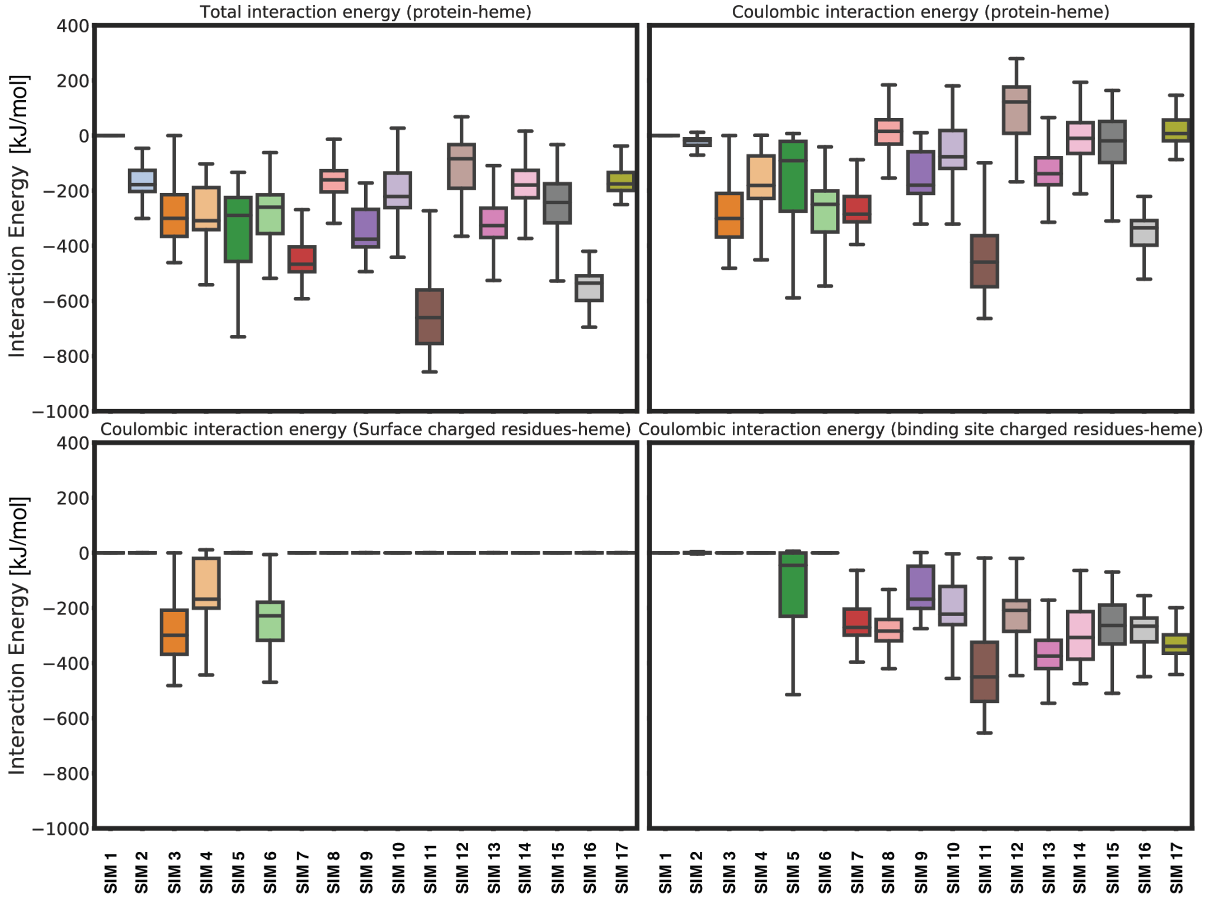

Supplementary Fig. 13 – Interaction analysis of heme with CydDC during MD simulations. The plots show non-bonded interaction energies between heme and the protein. The nonbonded interaction energies between the two parts were calculated using the g_energy module of GROMACS. The sum of the short-range Coulombic and Lennard-Jones energies and the short-range Coulombic energy between heme and the CydDC protein are shown in the upper left and upper right panels, respectively. The short-range Coulombic energy between heme and the positively charged residues on the protein surface ($K3^{D}$, $K7^{D}$, $K192^{D}$, and $R192^{D}$), heme and the positively charged residues in the binding site ($R73^{C}$, $R77^{C}$, $R81^{C}$, $R136^{C}$, and $R305^{D}$) are shown in the lower left and lower right panels, respectively. The box for each simulation shows the lower to upper quartile values of the data. The whiskers show the range of the data. The black line inside the box is the median of the data. Please see Supplementary Table 4 for details of the simulation setups (SIM 1-17).

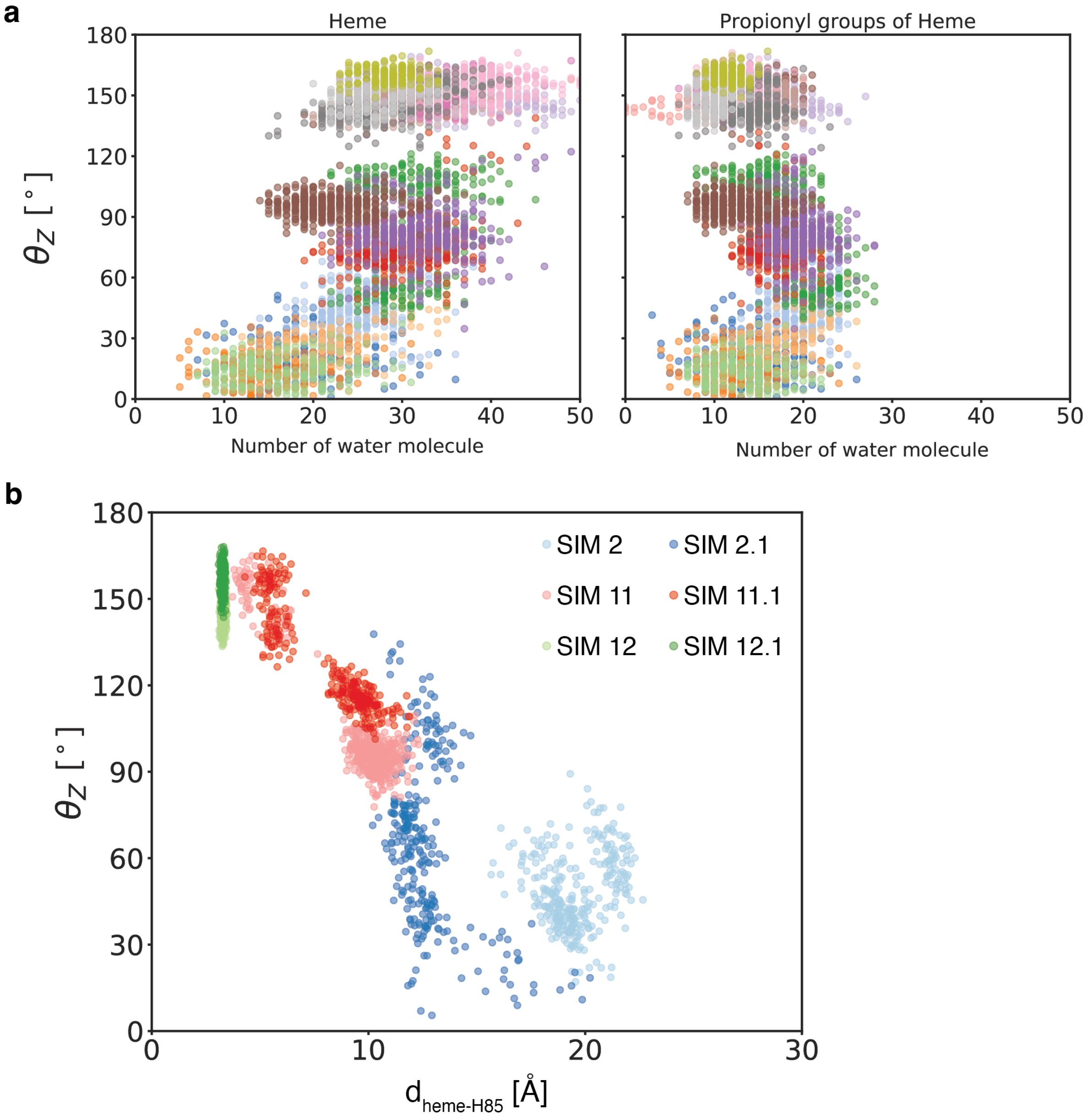

Supplementary Fig. 14 – Effect of hydration and site-specific mutations on the orientation of heme during the process of binding to CydDC. **(a)** Distribution of heme rotation angles (Θ_Z) versus number of water molecules within 4 Å of heme (left) or the propionyl groups of heme (right) in the different molecular dynamics simulation setups of CydDC. **(b)** Distribution of heme rotation angles (Θ_Z) versus heme distance from the binding site ($d_{heme-H85}$) in molecular dynamics simulations of three mutations of CydDC and their corresponding wt setups. $d_{heme-H85}$ is defined as the distance between the centers of mass of heme molecules and the side chain of H85^C. Θ_Z is defined as the angle between two vectors: vector 1 is axis normal to the membrane and vector 2 is the vector connecting CHA and CHC atoms in heme. SIM 11.1 and SIM 11 are the simulations of the mutation of R136^C and wild-type CydDC variants in the $\mathbf{IF}^{heme}_{confined}$ conformation in the absence of axial ligation of the central iron by nearby histidines, respectively. SIM 12.1 and SIM 12 are the simulations of the mutation of R77^C and R81^C in the $\mathbf{IF}^{heme}_{confined}$ and wild-type conformations with heme axially coordinated by histidines, respectively. SIM 2.1 and SIM 2 are the simulations of the alanine mutation at positions K3^C, K7^C, and K314^C in the $\mathbf{IF}^{apo}_{as\ isolated}$ conformation and its wild-type variant without heme near the lateral entry site. Please see Supplementary Table 4 for details of the simulation setups.

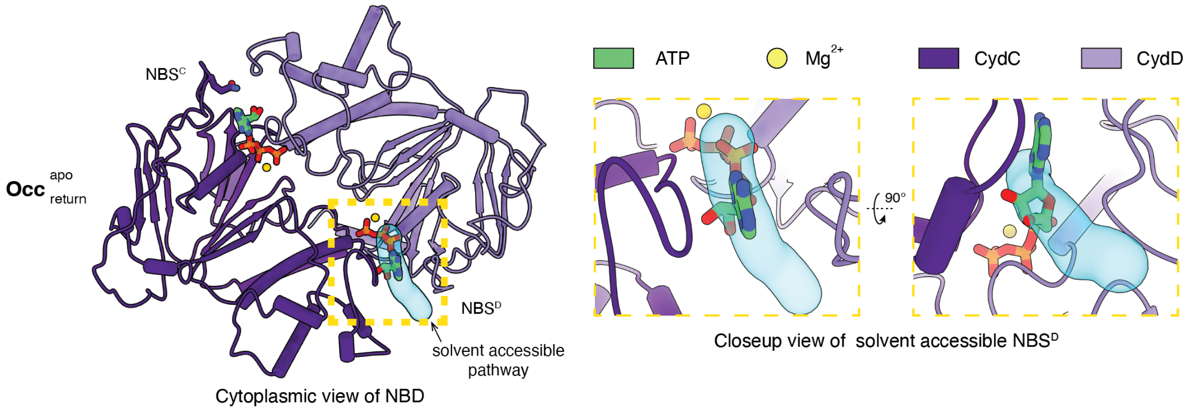

Supplementary Fig. 15 – Local environment and accessibility of NBS^{D} and NBS^{C}. Left: ribbon model of the interlocked NBD dimer in the $\mathbf{Occ}^{apo}_{return}$ state from turnover dataset of CydDC^{E500Q}. A predicted pathway between the aqueous environment and NBS^{D} is depicted in blue. Right: closeup views of NBS^{D} in side and top view orientation show that bound ATP is not shielded from the local environment. Weaker atomic interactions and exposure to the surrounding environment explain the observed reduced occupancy and possibility of exchange of nucleotide at NBS^{D} in the $\mathbf{Occ}^{apo}_{return}$ state. ATP, green; Mg^{2+}, yellow; CydC, light purple; CydD, dark purple.

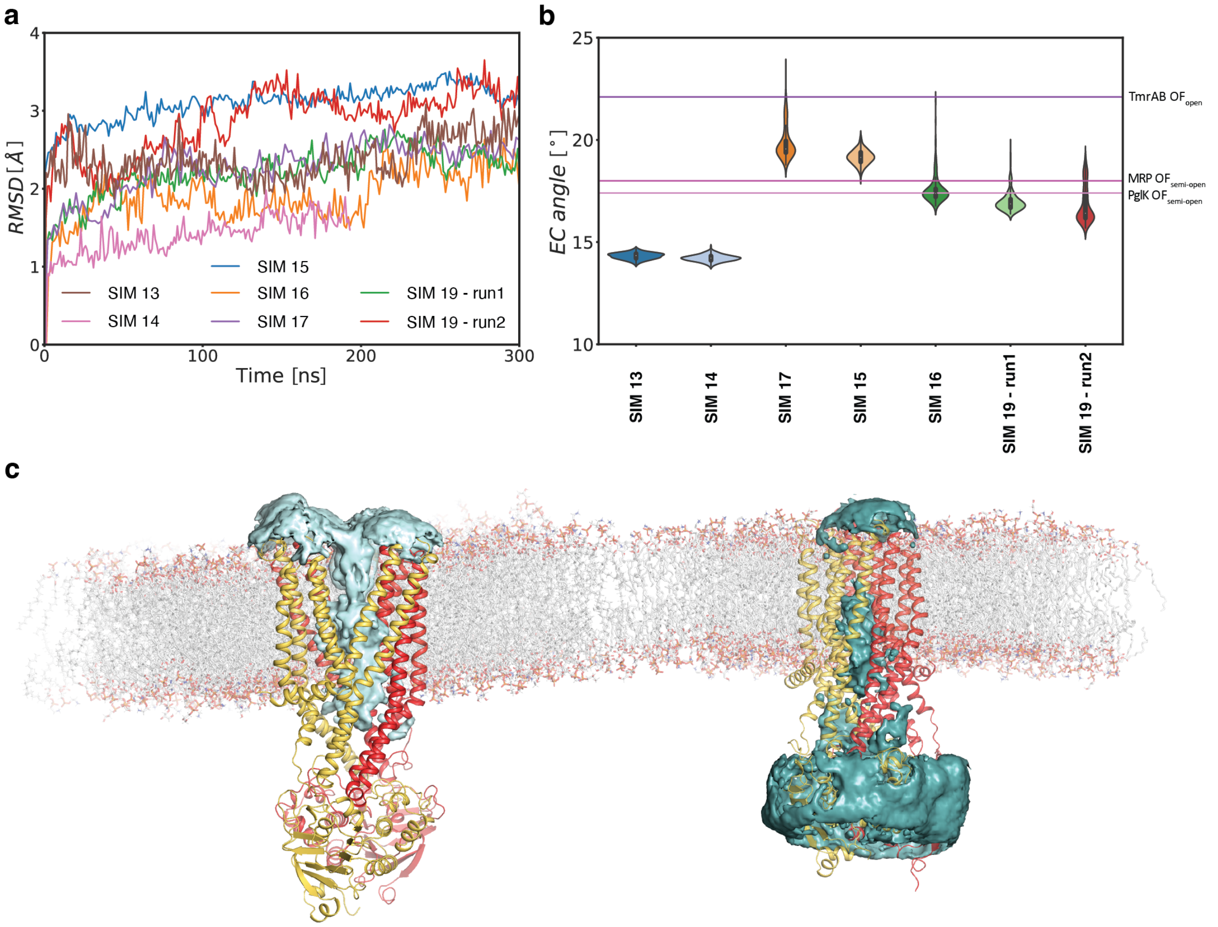

Supplementary Fig. 16 – Structural and conformational stability of different CydDC OF conformations. (a) Time trace of the Cα RMSD of the CydDC complex. The frames were first superimposed into the corresponding model obtained from the slow growth method and the steered MD simulations using the Cα atoms for $\mathbf{Occ}^{\text{heme}}_{\text{confined}}$ and $\mathbf{OF}^{\text{heme}}_{\text{confined}}$ conformations, respectively. Then the RMSD was calculated. **(b)** The comparison of the extracellular gate opening based the extracellular gate angle (EC angle) for different outward and occluded states. We define the EC angle as the angle between two axes: axis 3 between the centers of mass of the NBDs and the extracellular parts $TM1^C$, $TM2^C$, $TM3^C$, $TM4^C$, $TM5^D$, and $TM6^D$; and axis 4 between the center of mass of the NBDs and of the extracellular parts $TM1^D$, $TM2^D$, $TM3^D$, $TM4^D$, $TM5^C$, and $TM6^C$. The NBD distance is between the centers of mass of two NBDs (N-terminal loops and C-terminal helices were excluded). Three horizontal lines are marking the degree of the extracellular gate opening in outward-facing conformations of three other ABC exporters namely TmrAB (PDB ID 6RAJ), MRP1 (PDB ID 6UY0), PglK (PDB ID 5C73). The data is shown as a violin plot which is a hybrid of a box plot and a kernel density plot. The boxplot in the middle shows the median (the small white dot in the middle) and the interquartile range (first and third quartile). The thin gray line represents the rest of the distribution ending at the minimum and maximum on the two ends. The kernel density estimation shows the distribution shape of the data. **(c)** Representative view of the solvent/membrane-exposed cavity of outward-facing CydDC ($\mathbf{OF}^{\text{heme}}_{\text{confined}}$) (left) and the sealed cavity of occluded CydDC ($\mathbf{Occ}^{\text{heme}}_{\text{confined}}$) as observed during the MD simulations (300 ns). During the simulations of the OF states often one or more lipid molecules were intruding into the cavity, therefore they were included in calculating the volume map of the exposed cavity. Please see Supplementary Table 4 for details of the simulation setups.

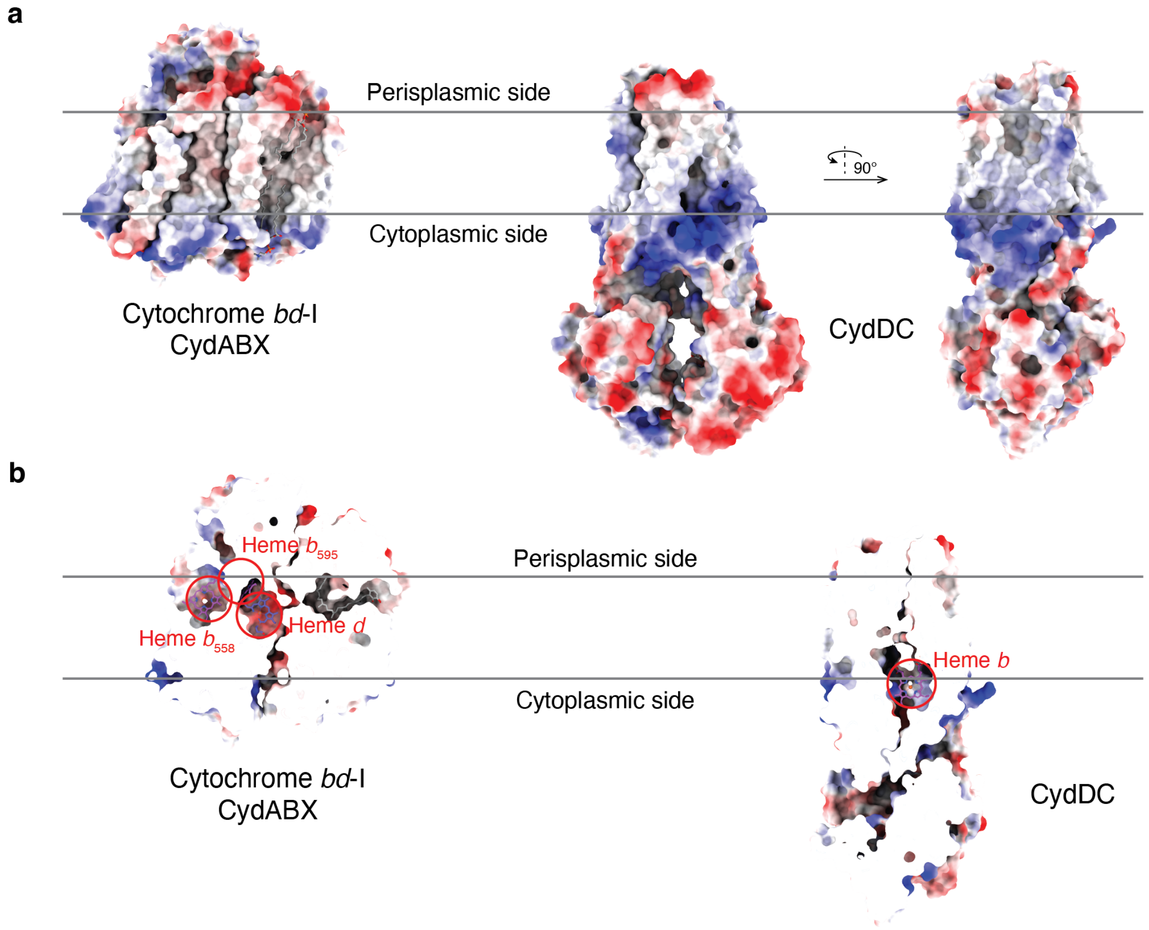

Supplementary Fig. 17 – Predicted localization of *E. coli* cytochrome *bd*-I and CydDC heme groups within membranes. (a) Electrostatic surface models of cytochrome *bd*-I and CydDC of *E. coli*. The electrostatic surface potential was calculated by ChimeraX (APBS) and shown from -10 kT/e (red, most anionic) to +10 kT/e (blue, most cationic), uncharged (white). **(b)** Relative positions of heme molecules in cytochrome *bd*-I and CydDC based on predicted membrane embedded regions of the proteins. Side views relative to the membrane at the levels of heme show that heme molecules rather cluster near the periplasmic membrane surface in cytochrome *bd*-I, while protoheme (heme *b*) locates near the surface of the cytoplasmic membrane leaflet in the inward-facing CydDC conformation.

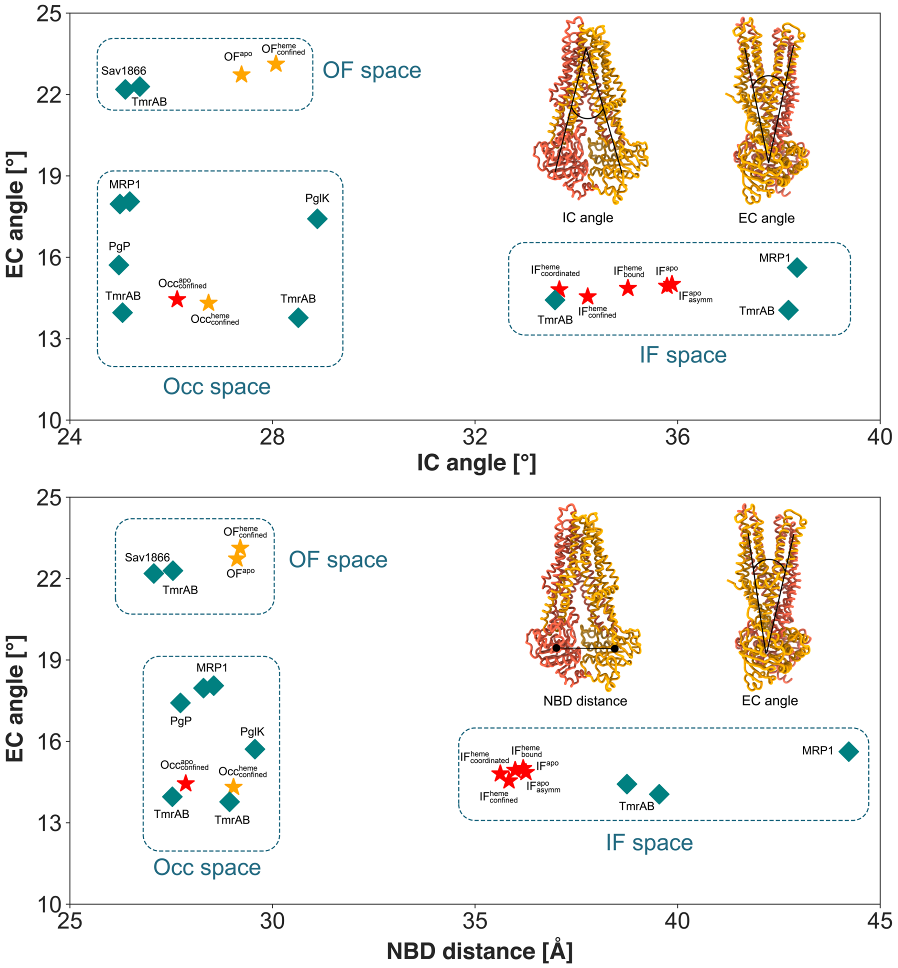

Supplementary Fig. 18 – Conformational space of ABC transporters. Conformation space of type IV ABC transporters defined based on three parameters: the intracellular gate angle (IC angle), the extracellular gate angle (EC angle), and the distance between two nucleotide-binding domains (NBD distance). Cryo-EM structures from this study are displayed as red stars, the three modeled conformations are shown as orange stars, previously published structures of ABC transporters are presented as teal diamonds. The clustering of structures in three major conformations [(inward-facing (IF), outward-facing (OF), and occluded (Occ)] is highlighted by dashed rectangles. The IC angle is described as the angle between two axes: axis 1 between the center of mass of the extracellular part of the TMH domain and the center of mass of the intracellular parts of TM1^{C}, TM2^{C}, TM3^{C}, TM6^{C}, TM4^{D} and TM5^{D}; and axis 2 between the center of mass of the whole extracellular part of the NBD region and the center of mass of the intracellular parts of TM1^{D}, TM2^{D}, TM3^{D}, TM6^{D}, TM4^{C}, and TM5^{C}. We define the EC angle as the angle between two axes: axis 3 between the centers of mass of the NBDs and the extracellular parts TM1^{C}, TM2^{C}, TM3^{C}, TM4^{C}, TM5^{D}, and TM6^{D}; and axis 4 between the center of mass of the NBDs and of the extracellular parts TM1^{D}, TM2^{D}, TM3^{D}, TM4^{D}, TM5^{C}, and TM6^{C}. The NBD distance is between the centers of mass of two NBDs (N-terminal loops and C-terminal helices were excluded). PDB IDs: TmrAB: 6RAG (IF wide), 6RAF (IF narrow), 6RAM (Occ), 6RAK (Occ), and 6RAJ (OF); MRP1, 5UJA (IF), 6BHU (Occ), and 6UY0 (Occ); Pgp, 6C0V (Occ); PglK, 5C73 (Occ); and Sav1866, 2ONJ (OF).
