# Supplementary material for: Dissecting the conformational complexity and mechanism of a bacterial heme transporter
Source: Nat Chem Biol. 2023 Apr 24;19(8):992–1003. doi: 10.1038/s41589-023-01314-5 (PMC10374445; doi:10.1038/s41589-023-01314-5)
Supplement: Supplementary file 2 — Reporting Summary [file 41589_2023_1314_MOESM2_ESM.pdf]

nature portfolio

Corresponding author(s): Dr. Schara Safarian

Last updated by author(s): Mar 2, 2023

Reporting Summary

Nature Portfolio wishes to improve the reproducibility of the work that we publish. This form provides structure for consistency and transparency in reporting. For further information on Nature Portfolio policies, see our Editorial Policies and the Editorial Policy Checklist.

Statistics

For all statistical analyses, confirm that the following items are present in the figure legend, table legend, main text, or Methods section.

n/a	Confirmed	
☐	☒	The exact sample size (n) for each experimental group/condition, given as a discrete number and unit of measurement
☐	☒	A statement on whether measurements were taken from distinct samples or whether the same sample was measured repeatedly
☐	☒	The statistical test(s) used AND whether they are one- or two-sided<br>*Only common tests should be described solely by name; describe more complex techniques in the Methods section.*
☒	☐	A description of all covariates tested
☒	☐	A description of any assumptions or corrections, such as tests of normality and adjustment for multiple comparisons
☐	☒	A full description of the statistical parameters including central tendency (e.g. means) or other basic estimates (e.g. regression coefficient) AND variation (e.g. standard deviation) or associated estimates of uncertainty (e.g. confidence intervals)
☐	☒	For null hypothesis testing, the test statistic (e.g. F, t, r) with confidence intervals, effect sizes, degrees of freedom and P value noted<br>*Give P values as exact values whenever suitable.*
☐	☒	For Bayesian analysis, information on the choice of priors and Markov chain Monte Carlo settings
☒	☐	For hierarchical and complex designs, identification of the appropriate level for tests and full reporting of outcomes
☒	☐	Estimates of effect sizes (e.g. Cohen's d, Pearson's r), indicating how they were calculated

Our web collection on statistics for biologists contains articles on many of the points above.

Software and code

Policy information about availability of computer code

Data collection	Titan Krios microscope operated at 300 kV and equipped with a BioQuantum energy filter and a K2/K3 camera (Gatan); Data collection quality was monitored through EPU v. 2.9-2.11 and CryoSparc Live v3.0. UV-Vis spectra were recorded on a Varian Cary 50 UV-Vis Spectrophotometer. Thermal stabilities of purified CydDC variants were investigated with a Prometheus NT.48. Kinetic turnover of ATPase activity via the PK/LDH assay was monitored at 340 nm using the SpectraMax M2 Microplate Reader. For the Malachite green based phosphate assay,absorbance change at 620 nm was measured via a SpectraMax M2 Microplate Reader. Cell growth was monitored via a SpectraMax Plus 384 Microplate Reader.
Data analysis	ChimeraX v.1.3, MotionCor2-2.1.2.6, Gctf v. 1.06, CLUSTAL Omega v.1.2.4 , RELION-3.1; CryoSPARC Live v3.0; COOT v. 0.8.9; Phenix (v. 1.14 & v. 1.18); MolProbity v. 4.5; CHARMM36m force field was used for protein, lipids, heme and ions, together with TIP3P water; Visual Molecular Dynamics (VMD) v. 1.9.2; GROMACS v. 2021.2; Jalview v. 2.11.2.0; TMalign v. 20210224; Spectroscopic data was analyzed via Origin Lab Pro 9.5, tunnels and cavities were mapped with MOLE 2.5; GraphPad v. 9.5.1 t-test calculator (https://www.graphpad.com/quickcalcs/ ttestl/?format=SD).

For manuscripts utilizing custom algorithms or software that are central to the research but not yet described in published literature, software must be made available to editors and reviewers. We strongly encourage code deposition in a community repository (e.g. GitHub). See the Nature Portfolio guidelines for submitting code & software for further information.

Data

Policy information about availability of data

All manuscripts must include a data availability statement. This statement should provide the following information, where applicable:

- Accession codes, unique identifiers, or web links for publicly available datasets
- A description of any restrictions on data availability
- For clinical datasets or third party data, please ensure that the statement adheres to our policy

Cryo-EM maps are deposited at the Electron Microscopy Data Bank under accession numbers: EMD-14636, EMD-14638, EMD-14639, EMD-14640, EMD-14641, EMD-14642, EMD-14643, EMD-14644, EMD-14645, EMD-14646, EMD-14647, EMD-14649, EMD-14652, EMD-14653, EMD-14654, EMD-14655, EMD-14656, EMD-14657, EMD-14659, EMD-14660, EMD-14662, EMD-14663, EMD-14665, EMD-14667, EMD-14668, EMD-14669, EMD-14670, EMD-14671, EMD-14672, EMD-14673, EMD-14674, EMD-14675, EMD-14676, EMD-14684, EMD-14689, EMD-15264, EMD-15265. Atomic models of CydDC have been deposited to the Protein Data Bank under accession numbers: 7ZD5, 7ZDA, 7ZDB, 7ZDC, 7ZDE, 7ZDF, 7ZDG, 7ZDK, 7ZDL, 7ZDR, 7ZDS, 7ZDT, 7ZDU, 7ZDV, 7ZDW, 7ZE5, 7ZEC. PDB models 6RKO and 7YO2 were used to generate figure 1f.

Field-specific reporting

Please select the one below that is the best fit for your research. If you are not sure, read the appropriate sections before making your selection.

☒ Life sciences ☐ Behavioural & social sciences ☐ Ecological, evolutionary & environmental sciences

For a reference copy of the document with all sections, see nature.com/documents/nr-reporting-summary-flat.pdf

Life sciences study design

All studies must disclose on these points even when the disclosure is negative.

Sample size	Sample sizes (number of collected micrographs) of respective cryo-EM datasets were chosen based on instrument availability and experimental design. Datasets of > 2000 micrographs ensured a sufficient number of particles to achieve resolutions < 3.5 Å. The smallest collected dataset contained 2294 micrographs (dataset 21) while the largest dataset contained 24110 micrographs (dataset 2). Oxygen consumption measurements and ATPase assays were performed in technical replicates (n = 3). Technical replicates were chosen to determine standard deviation values for each data points and to validate data quality.
Data exclusions	No data were excluded.
Replication	Single particle cryo-EM is based on averaging protein particles of nearly identical orientation within a vitreous layer of ice. Therefore, replication is not per se required to ensure statistical robustness of structural data. In case of this work, we have determined three individual structures of CydDC under 23 different sample conditions. Hence these data can be considered as biological replicates of the presented structural data.<br><br>Experimental findings of cell grwoth complementation, ATPase measurements, oxygen consumption measurements and thermal stability analyses were reproduced reliably.
Randomization	Generally, no randomization was required for the experimental design of this study. However, it is to note that particles are randomized during data processing steps in Relion-3.1 (randomization during 2D classification, randomized half sets during Refine3D). Randomized half sets of particles are used in final reconstruction steps in order to determined gold-standard Fourier shell correlations based on the 0.143 level.
Blinding	For all studies in this manuscript, such as 3D reconstruction, ATPase assays, or growth complementation, there was no awareness of group assignment that caused biased results, so blinding was was relevant for data reliability.

Reporting for specific materials, systems and methods

We require information from authors about some types of materials, experimental systems and methods used in many studies. Here, indicate whether each material, system or method listed is relevant to your study. If you are not sure if a list item applies to your research, read the appropriate section before selecting a response.

Materials & experimental systems

n/a	Involved in the study	
☒	☐	Antibodies
☒	☐	Eukaryotic cell lines
☒	☐	Palaeontology and archaeology
☒	☐	Animals and other organisms
☒	☐	Human research participants
☒	☐	Clinical data
☒	☐	Dual use research of concern

Methods

n/a	Involved in the study	
☒	☐	ChIP-seq
☒	☐	Flow cytometry
☒	☐	MRI-based neuroimaging
